# Novel 5-Substituted 2-(Aylmethylthio)-4-chloro-*N*-(5-aryl-1,2,4-triazin-3-yl)benzenesulfonamides: Synthesis, Molecular Structure, Anticancer Activity, Apoptosis-Inducing Activity and Metabolic Stability

**DOI:** 10.3390/molecules21060808

**Published:** 2016-06-22

**Authors:** Beata Żołnowska, Jarosław Sławiński, Aneta Pogorzelska, Krzysztof Szafrański, Anna Kawiak, Grzegorz Stasiłojć, Mariusz Belka, Szymon Ulenberg, Tomasz Bączek, Jarosław Chojnacki

**Affiliations:** 1 Department of Organic Chemistry, Medical University of Gdańsk, Al. Gen. J. Hallera 107, Gdańsk 80-416, Poland; anetapogorzelska@gumed.edu.pl (A.P.); k.szafranski@gumed.edu.pl (K.S.); 2 Department of Biotechnology, Intercollegiate Faculty of Biotechnology, University of Gdańsk and Medical University of Gdańsk, ul. Abrahama 58, Gdańsk 80-307, Poland; kawiak@biotech.ug.edu.pl; 3 Laboratory of Human Physiology, Medical University of Gdańsk, ul. Tuwima 15, Gdańsk 80-210, Poland; 4 Laboratory of Cell Biology, Department of Medical Biotechnology, Intercollegiate Faculty of Biotechnology UG-MUG, Medical University of Gdańsk, ul. Dębinki 1, Gdańsk 80-211, Poland; gstasilojc@gumed.edu.pl; 5 Department of Pharmaceutical Chemistry, Medical University of Gdańsk, Al. Gen. J. Hallera 107, Gdańsk 80-416, Poland; mariusz.belka@gumed.edu.pl (M.B.); szymon.ulenberg@gumed.edu.pl (S.U.); tbaczek@gumed.edu.pl (T.B.); 6 Department of Inorganic Chemistry, Gdańsk University of Technology, Narutowicza 11/12, Gdańsk 80-233, Poland; jarekch@pg.gda.pl

**Keywords:** sulfonamide, synthesis, metabolic stability, anticancer, apoptosis, mitochondrial membrane potential

## Abstract

A series of novel 5-substituted 2-(arylmethylthio)-4-chloro-*N*-(5-aryl-1,2,4-triazin-3-yl) benzenesulfonamide derivatives **27**–**60** have been synthesized by the reaction of aminoguanidines with an appropriate phenylglyoxal hydrate in glacial acetic acid. A majority of the compounds showed cytotoxic activity toward the human cancer cell lines HCT-116, HeLa and MCF-7, with IC_50_ values below 100 μM. It was found that for the analogues **36**–**38** the naphthyl moiety contributed significantly to the anticancer activity. Cytometric analysis of translocation of phosphatidylserine as well as mitochondrial membrane potential and cell cycle revealed that the most active compounds **37** (HCT-116 and HeLa) and **46** (MCF-7) inhibited the proliferation of cells by increasing the number of apoptotic cells. Apoptotic-like, dose dependent changes in morphology of cell lines were also noticed after treatment with **37** and **46**. Moreover, triazines **37** and **46** induced caspase activity in the HCT-116, HeLa and MCF-7 cell lines. Selected compounds were tested for metabolic stability in the presence of pooled human liver microsomes and NADPH, both R^2^ and Ar = 4-CF_3_-C_6_H_4_ moiety in 2-(R^2^-methylthio)-*N*-(5-aryl-1,2,4-triazin-3-yl)benzenesulfonamides simultaneously increased metabolic stability. The results pointed to **37** as a hit compound with a good cytotoxicity against HCT-116 (IC_50_ = 36 μM), HeLa (IC_50_ = 34 μM) cell lines, apoptosis-inducing activity and moderate metabolic stability.

## 1. Introduction

For decades chemotherapeutics have played the most important role in the fight against cancer. Unfortunately, the serious toxicities of conventional cytotoxic medicines have impelled and are still impelling researchers to focus on the development of new potent and selective anticancer drugs.

**Figure 1:**
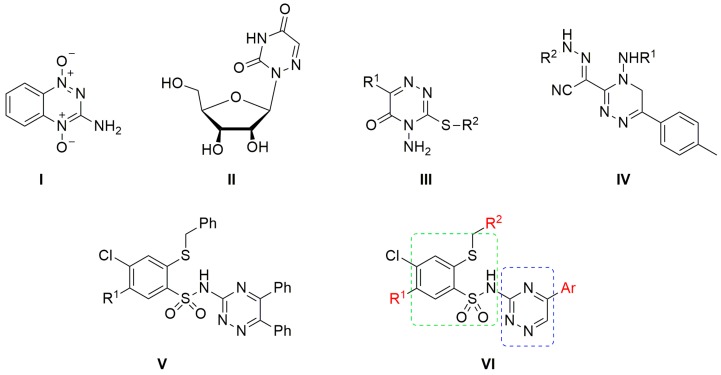
Structures of known anticancer 1,2,4-triazines (**I**–**V**) and novel 2-(arylmethylthio)-4-chloro-*N*-(5-aryl-1,2,4-triazin-3-yl)benzenesulfonamide derivatives (**VI**).

Heterocyclic scaffolds, especially nitrogen-containing heterocyclic compounds, play an important role in the design of novel drugs because of their utility for various biological receptors with a high degree of binding affinity. Among the heterocycles, the triazines, with their wide biological profile, occupy a prominent position [1]. The 1,2,4-triazine ring as one of the most ubiquitous heterocycles in Nature, and it has been reported to possess a broad spectrum of biological properties, including anticonvulsant [2], neuroprotective [3], sedative [4], anxiolytic [5], benzodiazepine receptor inhibitory activity [6], antiparkinson [7], antidepressant [8], anti-inflammatory [9], antimicrobial [10], antiparasitic [11] activities. Some 1,2,4-triazines have become well-known drugs, such as the antiviral azaribine [12], the anticonvulsant lamotrigine [13], the anti-inflammatory and analgesic apazone [14], the antibacterial ceftriaxone [15], the insecticide pymetrozine [16] or vardenafil, useful in the treatment of male erectile dysfunction [17]. In addition, there are also many reports indicating significant anticancer properties for the 1,2,4-triazine fragment. For example, tirapazamine (**I**, Figure 1) is currently in various clinical trial phases for the treatment of human non-small cell, cervical, ovarian, head and neck cancers. Tirapazamine works by inducing DNA damage in poorly oxygenated tumor cells [18]. The 6-azauridine **II**, known as an inhibitor of *de novo* pyrimidine biosynthesis, is an effective agent for remission induction of acute myelocytic leukemia in children [19]. Close analogs of 6-azauridine, *S*-alkyl derivatives of 1,2,4-triazinone **III**, show cytotoxic activities against human breast cancer (MCF-7), colon carcinoma (HCT-116) and hepatocellular carcinoma (Hep-G2) cell lines [20]. Some 1,2,4-triazine analogs **IV** have distinct antiptoliferative activities against non-small cell lung cancer (NCI-H460), breast cancer (MCF-7), CNS cancer (SF-268), with a low cytotoxicity [21].

Our systematic studies on arylsulfonamides has resulted in promising anticancer agents [22,23,24,25,26,27,28,29], and among them we have reported that some trisubstituted 1,2,4-triazines **V** (Figure 1), exhibited anticancer activity against colon, CNS, melanoma, ovarian, breast, renal, leukemia cell lines [29]. Searching for innovative low-molecular chemotherapeutics, a structure-based molecular hybridization strategy has been applied as a common approach to develop multi-targeted compounds [30]. Thus, we designed and synthesized a series of new 4-chloro-2-mercapto-*N*-(5-aryl-1,2,4-triazin-3-yl)benzenesulfonamides **VI** (Figure 1), bearing a disubstituted 1,2,4-triazine and a 2-mercaptobenzenesulfonamide moiety in the molecular skeleton, (Figure 1) as potential anticancer agents. Various substituent groups were introduced to the triazine ring as well as the benzenesulfonamide fragment in positions 2 and 5 for structure-activity relationship (SAR) analysis. The prepared compounds were evaluated for their antiproliferative activities against three human cancer cell lines, namely, HCT-116 (colon cancer), HeLa (cervical cancer) and MCF-7 (breast cancer). For the most active compounds apoptosis- inducing activities on HCT-116, HeLa and MCF-7 cell lines were further investigated.

*In vitro* tests for metabolic stability, which is one of the important parameters characterizing pharmacokinetic properties of a molecule, were performed on selected compounds. The experiments were run in the presence of human liver microsomes and NADPH.

## 2. Results and Discussion

### 2.1. Chemistry

**Scheme 1:**
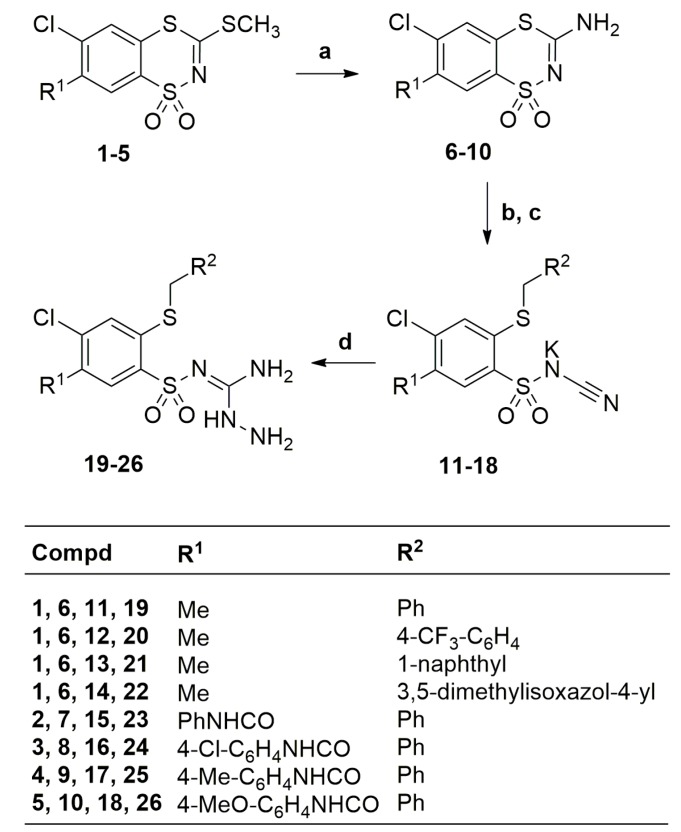
Synthesis of 1-amino-2-[4-chloro-5-R^1^-2-(R^2^-methylthio)benzenesulfonyl]guanidine (**19**–**26**). *Reagents and Conditions*: (**a**) 25% NH_3_ aq/EtOH, r.t., 144 h; (**b**) Excess K_2_CO_3_, THF, reflux, 24 h; (**c**) R^1^CH_2_Cl, ethanol or water, r.t. or 0 °C, 1–4 h; (**d**) NH_2_-NH_2_ × HCl, dry toluene, reflux 3–9 h.

**Scheme 2:**
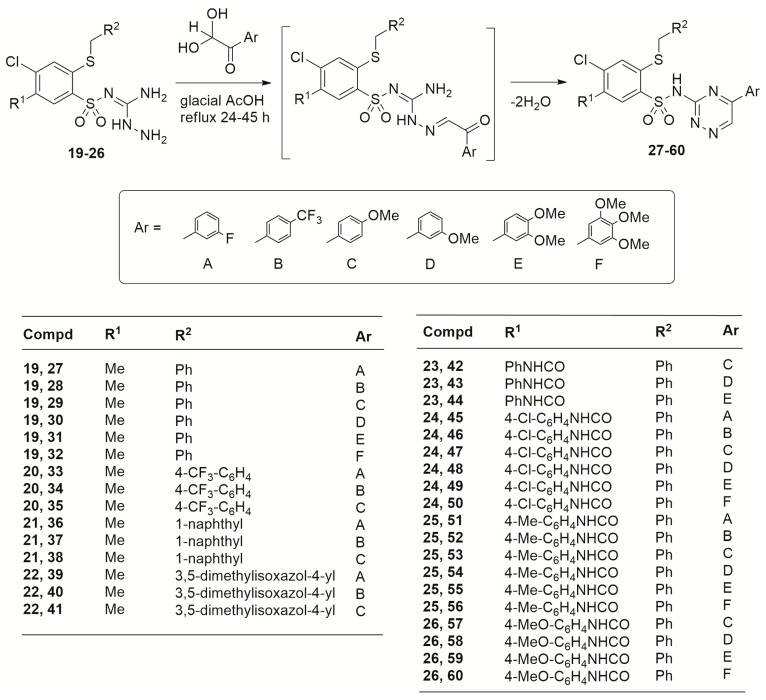
Synthesis of 4-chloro-2-(R^2^-methylthio)-5-R^1^-*N*-(5-aryl-1,2,4-triazin-3-yl)benzenesulfonamides **27**–**60**.

The starting substrates, 6-chloro-3-methylthio-7-R^1^-1,1-dioxo-1,4,2-benzodithiazines **1**–**5** [31,32], 3-amino-6-chloro-7-R^1^-1,1-dioxo-1,4,2-benzodithiazines **6**–**10** [33,34], *N*-(benzenesulfonyl)cyanamide potassium salts **11**–**18** [22,25,33,35] as well as aminoguanidines **19**–**20** and **23**–**25** [29,33,35,36] were prepared according to the respective known methods. Novel substrates **21**–**22** and **26** were synthesized analogously by the reaction of hydrazine monohydrochloride with the potassium salts **13**–**14** as was shown in Scheme 1. Finally, 4-chloro-2-(R^2^-methylthio)-5-R^1^-*N*-(5-aryl-1,2,4-triazin-3-yl)benzenesulfonamides **27**–**60** were readily obtained by treatment of aminoguanidines **19**–**26** with the appropriate phenylglyoxal hydrate in glacial acetic acid at reflux for 24–45 h as outlined in Scheme 2.

The structures of the final compounds **27**–**60** were confirmed by IR, ^1^H-NMR and ^13^C-NMR spectroscopy. ^1^H-NMR spectra showed the absence of characteristic of aminoguanidine NH signals at 4 and 6 ppm, confirming the success of the condensation reactions between the amine and carbonyl groups of the reagents leading to the heterocyclic ring formation. In addition, the presence in the ^1^H-NMR spectra of a singlet at about 9 ppm corresponding to the H-6 proton in the 1,2,4-triazine fragment was observed. Moreover, X-ray analysis was done to confirm the proposed structures using the representative compound **59**.

Compound **59** crystallizes in the triclinic system in the space group $P\bar{1}$. The asymmetric unit contains two sulfonamide molecules and four solvating dimethylformamide (DMF) molecules. The unit cell of the crystal contains two asymmetric parts (Z = 2). The two independent sulfonamide molecules have the same topological atom connectivity (constitution) but cannot be superimposed on each another (they are almost related by a mirror plane passing through SO_2_ group and bisecting the S–N–C and S–C–C planes). The solvent DMF molecules are not only filling the voids in the structure, but also participate in hydrogen bonding—two of carbonyl groups are acceptors of hydrogen from N-H of 1,2,4-triazine residue (O14-N4, O16-N9, see Table 1). The other two DMF molecules use their CO groups as acceptors for amidic NH groups (O13-N6, O15-N1, Table 1). Other intermolecular interactions responsible for crystal packing are π-π stacking operating between R1 (N3, C22, N4, N5, C23, C24) and R5 (C25–C30) with the distance between ring geometry centers (centroids) of 3.8252(3)Å and R6 (N8, C54, N9, N10, C55, C56) and R10 (C57–C52) 3.8952(3)Å. All other rings are separated by more than 4.4Å and interactions among them were neglected.

**Figure 2:**
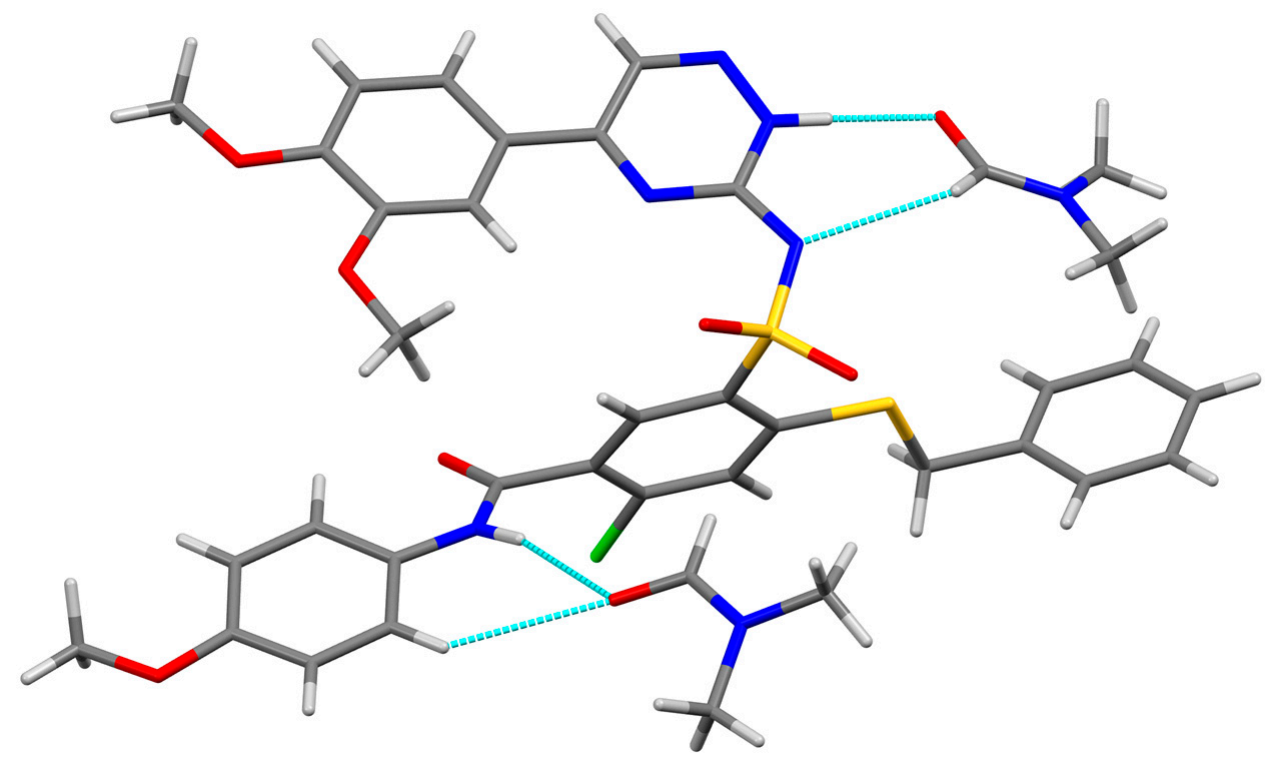
View of half of the independent unit (one sulfonamide and two DMF molecules) showing the general arrangement of atoms in **59** and N–H···O type hydrogen bonds.

The sulfonamide group seems to be deprotonated in the solid state, as there is a C–H bond from a DMF molecule directed to the sulfonamidic N (Figure 2) which would not be beneficial without assumed ionization of the –SO_2_NH– fragment. Additionally, protonation of the nitrogen atom in position 2 of the 1,2,4-triazine is required by electrical neutrality and by formation of hydrogen bond to the carbonyl group from the neighbor DMF molecule. Thus, two charge-assisted hydrogen bonds are created: C–H···N(−)–S and (+)N–H···O between the sulfonamide and the solvating DMF.

Bond lengths within the triazine ring are not helpful to attribute double bonds in the substructure. The 1,2,4-triazine residue is flat and almost coplanar with 3,4-dimethoxyphenyl which may be a consequence of beneficial π-π interactions with the neighbor molecule in crystal. The second solvent DMF molecule forms hydrogen bond with the amide N–H proton donor in the other branch of the main molecule. The other functional groups are not unusual and their geometry will therefore not be further analyzed.

### 2.2. Biological Evaluations

#### 2.2.1. Cytotoxic Activity

Compounds **27**–**60** were evaluated *in vitro* for their effects on the viability of three human cancer cell lines: HCT-116 (colon cancer), HeLa (cervical cancer) and MCF-7 (breast cancer). The concentration required for 50% inhibition of cell viability IC_50_ was calculated and compared with the reference drug cisplatin, the results were shown in Table 2. To describe of cytotoxic potency, the following scale was applied: IC_50_ < 25 μM—very strong, 25 ≤ IC_50_ ≤ 50 μM—strong, 50 < IC_50_ < 75 μM—moderate, 75 ≤ IC_50_ ≤ 100 μM—weak, IC_50_ >100 μM—inactive compounds.

The most active compounds **37** and **38** belonged to the 1-naphthyl series (R^2^ = 1-naphthyl) and showed outstanding average cytotoxic activity (47 μM and 50 μM, respectively) against all tested cell lines, HCT-116, HeLa and MCF-7. Moreover, compounds **34**–**36** and **46** moderately inhibited all cell lines viability, exhibiting average IC_50_ values below 71 μM. Among tested analogues only six compounds (17.6%) were completely inactive with average IC_50_ > 100 μM.

As shown in Table 2, the HCT-116 cell line presented the relatively highest susceptibility and was affected by eleven compounds (**33**–**38**, **46**, **47**, **52**, **56** and **57**) in the range of IC_50_ values 36–75 μM. Meanwhile, the HeLa cell line was susceptible toward six compounds (**34**–**38** and **53**) with IC_50_; 34–74 μM, and the MCF-7 by six compounds (**30**, **34**, **37**, **38**, **46** and **52**) with IC_50_ values ranging between 59 and 70 μM.

We found that, among 4-chloro-2-(R^2^-methylthio)-5-R^1^-*N*-(5-aryl-1,2,4-triazin-3-yl)benzene-sulfonamides with R^2^ = 1-naphthyl, presence of Ar = 4-CF_3_-C_6_H_4_ group provided strong cytotoxicity of **37** toward the HeLa (IC_50_ = 34 μM) as well as HCT-116 (IC_50_ = 36 μM) cells and replacement of 1-naphthyl substituent in this compound by the smaller aromatic group, *i.e.*, **34** (R^2^ = 4-CF_3_-C_6_H_4_), **40** (R^2^ = 3,5-dimethylisoxazol-4-yl) caused decrease in activity to IC_50_; 51–85 μM or the loss of activity when R^2^ = Ph (compd **28**, see Table 2). It should be noted, that replacement of Ar = 4-CF_3_-C_6_H_4_ group (**37**) by Ar = 4-MeO-C_6_H_4_ (**38**) or 3-F-C_6_H_4_ (**36**) did not contribute to improving the anticancer activity. The presence of Ar = 4-CF_3_-C_6_H_4_ also influenced the strongest cytotoxic activity of **46** (R^1^ = 4-Cl-C_6_H_4_NHCO, R^2^ = Ph, IC_50_ = 59 μM) toward the MCF-7 cell line. Nevertheless, the exchange of R^1^ = 4-Cl-C_6_H_4_NHCO in compd **46** to small methyl group strongly inactivate the compound **28** (R^1^ = Me, R^2^ = Ph) but introduction of 4-Me-C_6_H_4_NHCO as the R^1^ substituent in compd **52** decreased its cytotoxic activity to IC_50_ = 69 μM.

#### 2.2.2. Investigation of Apoptotic Activity

The ability to induce apoptosis in cancer cells is a desired feature of a potential chemotherapeutic agent. Thus, the apoptosis-inducing activity was studied through biochemical markers, such as: DNA fragmentation, loss of mitochondrial membrane potential (Δψ_m_), phosphatidylserine translocation and caspase activation. Apoptotic-like changes in morphology of tested cell lines were also evaluated. The experiments were performed with the most active compounds (**37** and **46**) exhibited the highest activity in MTT tests.

##### Cell Morphology

**Figure 3:**
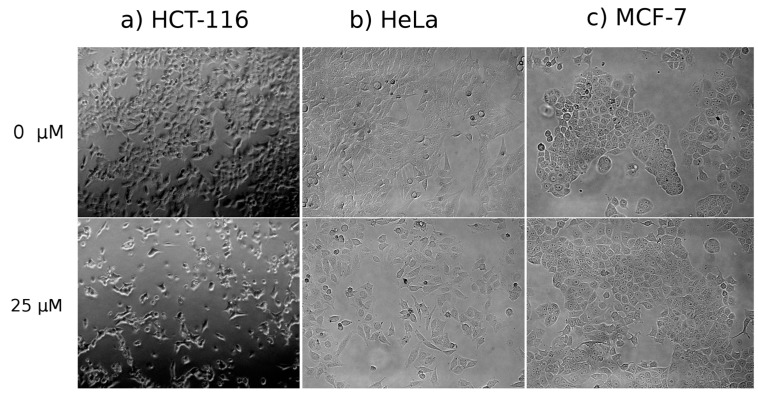

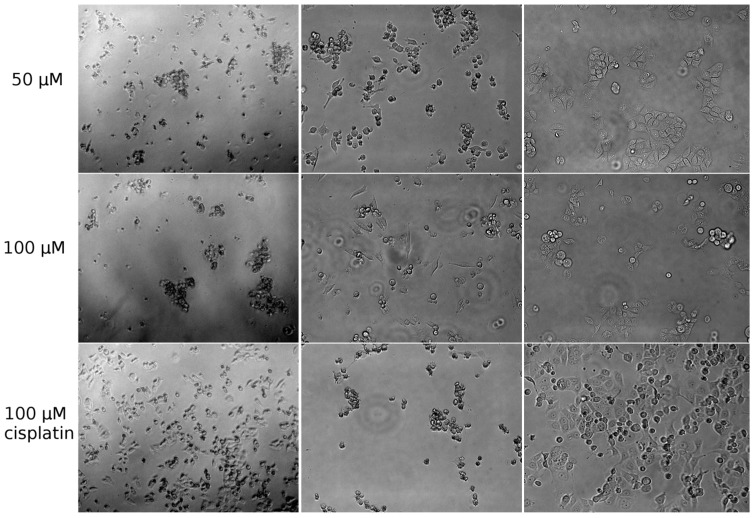
Morphology of cell lines: HCT-116 (**a**); HeLa (**b**) treated with **37** (0–100 μM) and cisplatin (100 μM); MCF-7 (**c**) treated with **46** (0–100 μM) and cisplatin (100 μM). Light microscopy photographs, with 10× objective, of cells treated for 24 h.

To evaluate the changes in morphology of the treated cells, they were incubated with increasing concentrations of **37** (HCT-116, HeLa) or **46** (MCF-7) for 24 h and observed using light microscope. The most characteristic morphological changes (shrinkage of the cells, detachment from the surface) occurred in the HCT-116 and HeLa cells treated with **37** (Figure 3).

##### Cell Cycle Analysis

One of the most common mechanisms in anticancer drug treatment is changes in cell cycle, which can be measured by DNA content [37]. Cell cycle distribution in HCT-116, HeLa and MCF-7 cells was examined to determine cycle arrest and/or presence of sub-G1 after treatment with compounds **37** and **46**. Cells were coincubated with trazines and a reference drug (cisplatin) for 24 h, respectively. Cell populations data are expressed as the mean ± SD of at least three independent experiments.

**Figure 4:**
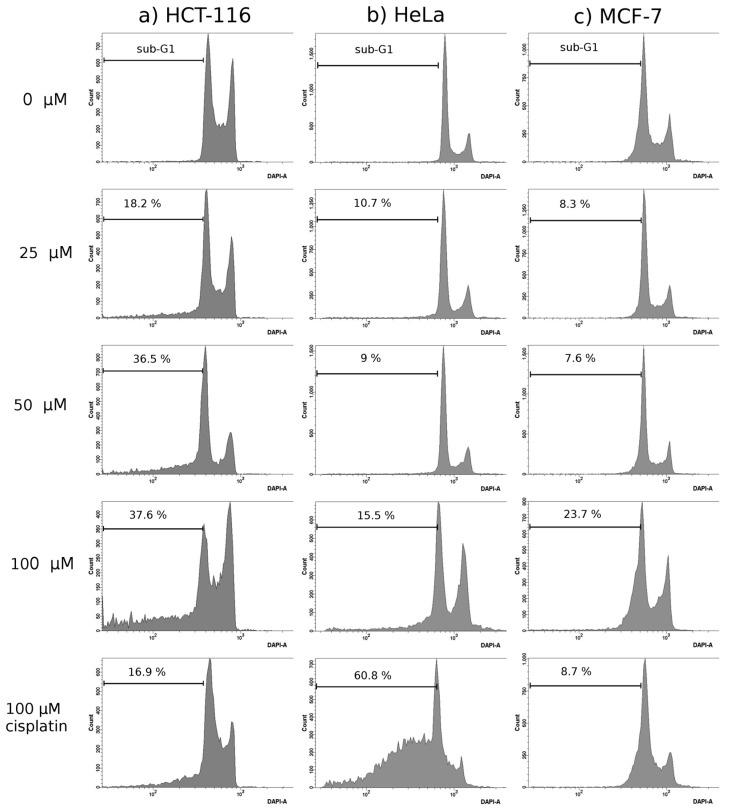
HCT-116, HeLa and MCF-7 cell cycle 24 h induction with 1,2,4-triazine derivatives (representative results). HCT-116 (**a**); HeLa (**b**) treated with **37** (0–100 μM) and cisplatin (100 μM); MCF-7 (**c**) treated with **46** (0–100 μM) and cisplatin (100 μM).

Sub-G1 cell population increased in dose dependent manner (HCT-116 from 3.6% ± 1.66% (control) to 48.6% ± 13.5%; HeLa from 5.7% ± 3.4% (control) to 21% ± 3.4%) after treatment with compound **37** for 24 h. Figure 4 shows one representative experiment. Similar results were obtained for MCF-7 cells coincubated with compound **46** for 24 h (from 5.0% ± 3.8% (control) to 20% ± 5.6%) (Figure 4).

Coincubation of HCT-116, HeLa and MCF-7 with appropriate triazines (**37** and **46**) caused appearance of sub-G1 stage and high concentrations led to fragmentation of DNA, resulting in a *sub*-G0/G1 peak of cell cycle. A significant accumulation of the HCT-116, HeLa and MCF-7 population in the sub-G1 phase indicated that the cells had undergone programmed cell death (apoptosis) for all of the analyzed compounds. Triazine coincubation did not cause significant differences in the cell cycle phases of the analyzed cell lines.

##### Mitochondrial Membrane Potential (Δψ_m_) Analysis

Loss of the mitochondrial membrane potential (Δψ_m_) is one of the earliest indicators of an apoptosis [38]. It can be detected using fluorescent dye JC-1 (5,5′,6,6′-tetrachloro-1,1′,3,3′-tetraethylbenzimidazolylcarbocyanine iodide) exited with blue laser. JC-1 spontaneously forms dimer complexes (J aggregates) with red fluorescent or remain as a monomers with green fluorescence in cells with high and low Δψ_m_, respectively.

**Figure 5:**
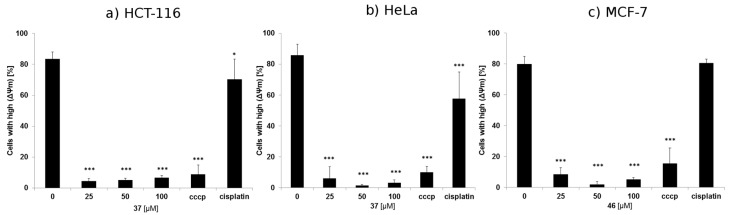
Mitochondrial membrane potential (Δψ_m_). Cytometric analysis of Δψ_m_ with 2.5 μg/mL JC-1 in: HCT-116 (**a**), HeLa (**b**) treated with **37** (0–100 μM) 24 h, carbonyl cyanide *m*-chlorophenylhydrazone (CCCP) (100 μM) for 15 min or cisplatin (100 μM) 24 h; MCF-7 (**c**) treated with **46** (0–100 μM) 24 h, CCCP (100 μM) for 15 min or cisplatin (100 μM) 24 h. Results represent the mean of number of cells with high Δψ_m_ (high JC-1 fluorescence) ± SD (*n* = 8). Statistically significant differences between treated and control cells are indicated (* *p* < 0.05; *** *p* < 0.005).

As shown in Figure 5 HCT-116, HeLa and MCF-7 cells in the control group, high red fluorescence (J aggregates) were observed. However, exposure of cells even to low and moderate concentrations of compounds **37** and **46** remarkably decreased Δψ_m_ which is the earliest indicators of an apoptotic cell death.

##### Translocation of Phosphatidylserine to Outer Leaflet of Cell Membrane

The quantification of cell death was evaluated by measuring the exposure of phosphatidylserine on the outer leaflet of plasma membrane. Four subpopulations were identified according to their fluorescence: PI-low/FITC-low (live cells), PI-high/FITC-low (necrotic cells), PI-low/FITC-high (early apoptotic cells), PI-high/FITC-high (late apoptotic cells).

**Figure 6:**
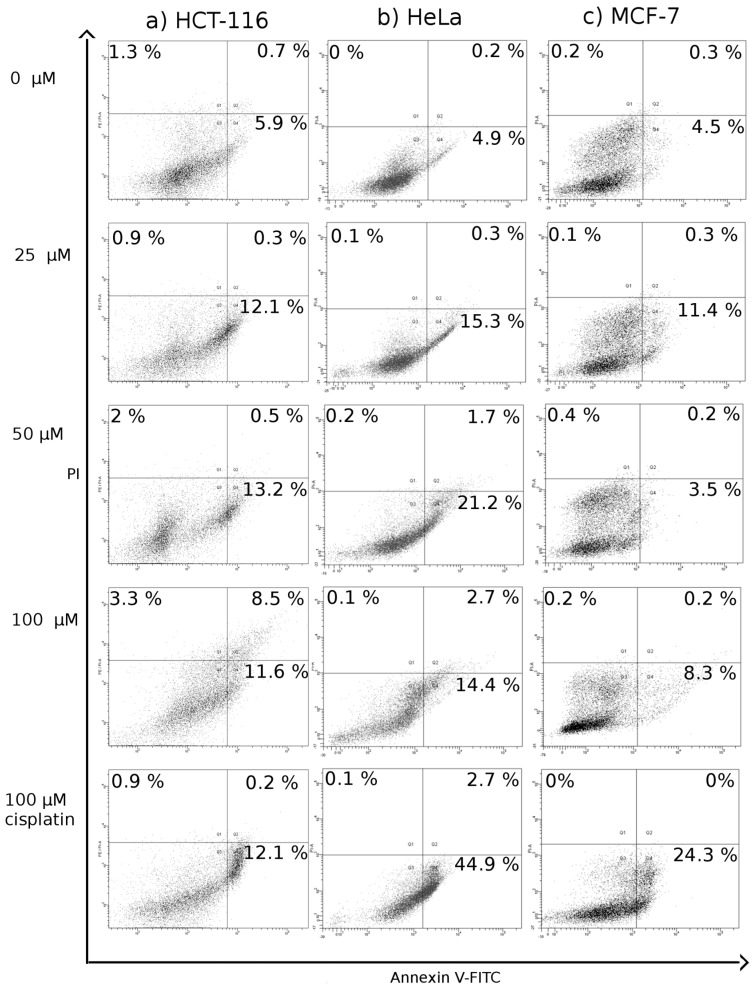
Features of dying process of HCT-116 (**a**); HeLa (**b**) and MCF-7 (**c**) cell lines (representative results). Dotblots show cells stained with Annexin V-FITC Apoptosis Kit. HCT-116, HeLa cells treated with **37** (0–100 μM) and cisplatin (100 μM); MCF-7 cells treated with **46** (0–100 μM) and cisplatin (100 μM) for 24 h. Results show the mean of number of cells in each quadrant Q1, Q2, Q3, Q4 (necrotic cells, late apoptotic cells, alive cells, apoptotic cells, respectively).

Appearance of increased population of PI-low/FITC-high (early apoptotic cells), PI-high/FITC-high (late apoptotic cells) gates was noticed for HCT-116 and HeLa cells coincubated with compound **37** (Figure 6). The increased levels of apoptotic cells were concentration dependent. For MCF-7 cells treated with compound **46** the results were not conclusive, as the differences between treated cells and control cells were statistically significant only for low 25 μM of **46** (Figure 6).

##### Caspase Activation

Caspase activation plays a central role in the execution of apoptosis and is required for the occurrence of its biochemical and morphological hallmarks, such as DNA fragmentation, formation of apoptotic bodies and chromatin condensation. The ability of the examined compounds to induce caspase activity was determined with the use of a fluorescent labeled caspase inhibitor, a carboxyfluorescein (FAM) derivative of valylalanylaspartic acid (VAD) fluoromethyl ketone (FMK).

**Figure 7:**
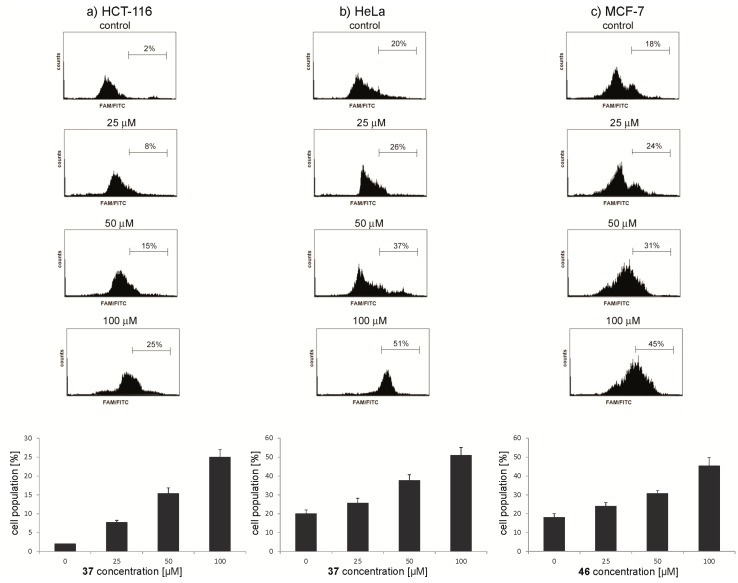
Induction of caspase activity in HCT-116, HeLa and MCF-7 cells by 1,2,4-triazine derivatives. HCT-116 (**a**), HeLa (**b**) and MCF-7 (**c**) cells were treated with the indicated concentrations of compounds **37** and **46** for 24 h (**a**), 72 h (**b**) and 72 h (**c**), respectively. Enzyme activity was determined by flow cytometry with the use of a caspase inhibitor, FAM-VAD-FMK. Values represent mean ± SD of three independent experiments.

FAM-VAD-FMK contains a target sequence recognized by active caspases (caspases 1 through 9). Binding to this sequence inhibits the enzymatic activity of caspases and allows for the determination of their activity through the direct measurement of fluorescent intensity of the bound inhibitor. The results of the research showed that the tested compounds **37** and **46** induced caspase activity in the examined cells lines, as shown by an increase in FAM-VAD-FMK fluorescence in the cell population with activated caspases (Figure 7). The strongest influence on caspase activation was noticed for the HeLa cell line, where that cell population increased by 31% in the presence of **37** at a concentration of 100 μM.

#### 2.2.3. Metabolic Stability

Metabolism as one of the drugs’ pharmacokinetic characteristics can be evaluated during the early preclinical stage to assessment of degree of drug candidate conversion into a set of metabolites. Metabolic stability can be assessed by incubation of a potent drug in a presence of liver microsomes and NADPH to give an insight to metabolic properties [39,40,41].

For further evaluation of metabolic stability the compounds with outstanding activities and varied structural features including R^1^, R^2^ and Ar substituents (**30**, **31**, **34**–**38**, **46**, **47**, **52**) were selected. Human liver microsomes were chosen as a model enzymatic system and *in vitro* metabolic half-life was assessed (Table 3).

Based on the enzymatic test results some structure-metabolic stability relationships can be noted. The most stable compound **34** bears a 4-CF_3_-C_6_H_4_ moiety in both the R^1^ and Ar positions. Replacement of Ar = 4-CF_3_-C_6_H_4_ (**34**) by 4-MeO-C_6_H_4_ (compound **35**) resulted in decrease of the t_1/2_ value from >60 to 42.4 min, while an analogous change between **46** and **47** caused a significant decrease of t_1/2_ from >60 to 17.5 min. On the other hand, taking into account the results for **30** (Ar = 3-MeO-C_6_H_4_) and **31** (Ar = 3,4-diMeO-C_6_H_3_), it was found that an additional methoxy (MeO) group in the Ar substituent slightly decreased the metabolic stability. These facts suggest that the methoxy substituent is a major soft spot in the described series of compounds and moreover, its undesirable influence can be diminished by incorporation of CF_3_ instead of a MeO substituent.

Comparison of t_1/2_ for **34** (R^1^ = 4-CF_3_-C_6_H_4_) and **37** (R^1^ = 1-naphthyl) indicated that 1-naphthyl (R^1^) also seems to decrease metabolic stability. We assumed that the undesirable influence of the 1-naphthyl (R^1^) and 4-MeO-C_6_H_4_ (Ar) substituents accelerated in the case of compound **38**, which was characterized as the least stable derivative. Further synthesis in this group of compounds should avoid incorporation of the two abovementioned substituents. Although a 4-CF_3_-C_6_H_4_ (R^1^) substituent is preferable for metabolic stability in comparison with a 1-naphthyl moiety, the high biological response observed for **37** predisposes it to be a lead compound, as it shows a good balance between stability and cytotoxic properties.

**Figure 8:**
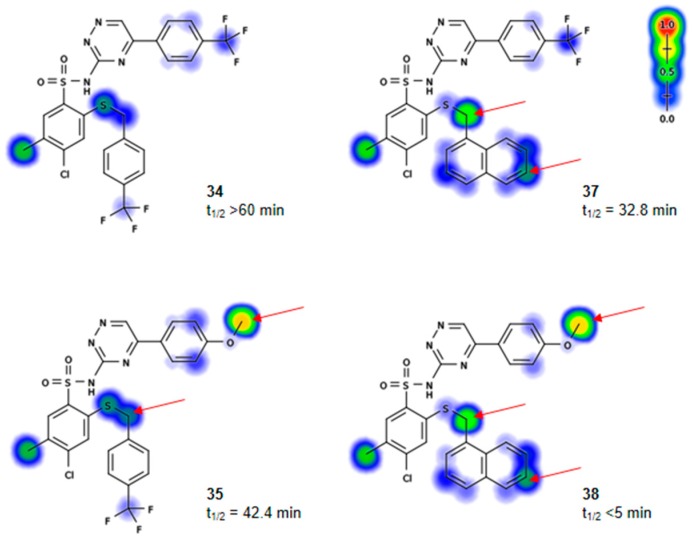
Sites of metabolism predicted for **34**, **35**, **37** and **38** by XenoSite software [42]. Yellow color indicates more vulnerability to biotransformation than blue. Some significant differences are additionally pointed out by red arrows.

In order to explain the differences between the metabolic stability of distinctive compounds (**34**–**35**, **37**–**38**) we applied a tool accessible on-line for accurate prediction of xenobiotic metabolism sites, called XenoSite Cytochrome P450 Prediction Models [42]. Among the available models, one is able to predict which atoms on a molecule are likely to be oxidized by human liver microsomes. *In silico* results showed that the least stable derivative **38** has three more sites vulnerable for metabolic biotransformation than **34** (see Figure 8). Moreover, a slight decrease of metabolic stability of **35** and **37** compared to **34** may resulted from presence of additional sites of oxidation on methoxy group and methylene linker (**35**, Figure 8) as well as on methylene linker and naphthalene ring (**37**, Figure 8).

## 3. Materials and Methods

### 3.1. General Information

The melting points were uncorrected and measured using Boethius PHMK apparatus (Veb Analytic, Dresden, Germany). IR spectra were measured on Thermo Mattson Satellite FTIR spectrometer (Thermo Mattson, Madison, WI, USA) in KBr pellets; an absorption range was 400–4000 cm^−1^. ^1^H-NMR and ^13^C-NMR spectra were recorded on a Varian Gemini 200 apparatus (Varian, Palo Alto, CA, USA) or a Varian Unity Plus 500 apparatus. Chemical shifts are expressed at δ values relative to Me_4_Si (TMS) as an internal standard. The apparent resonance multiplicity is described as: s (singlet), d (doublet), dd (doublet of doublets), t (triplet), m (multiplet), and br (broad) signal. Elemental analyses were performed on 2400 Series II CHN Elemental Analyzer (PerkinElmer, Shelton, CT, USA) and the results were within ±0.4% of the theoretical values. Thin-layer chromatography (TLC) was performed on Kieselgel 60 F254 plates (Merck, Darmstadt, Germany) and visualized by UV. The commercially unavailable substrates were obtained according to the following methods described previously: 6-chloro-3-methylthio-7-R^1^-1,1-dioxo-1,4,2-benzo-dithiazines **1**–**5** [31,32], 3-amino-6-chloro-7-R^1^-1,1-dioxo-1,4,2-benzodithiazines **6**–**10** [33,34], *N*-(2-alkylthio-4-chloro-5-R^1^-benzenesulfonyl)cyanamide potassium salts **11**–**18** [22,25,33,35], 1-amino-2-(4-chloro-5-R^1^-2-alkylthiobenzenesulfonyl)guanidines **19**–**20** and **23**–**25** [25,29,33,35,36]. The NMR spectra of newly synthesized compounds **21**–**22**, **26**–**60** are given in the Supplementary Materials.

### 3.2. Synthesis

#### 3.2.1. Procedures for the Preparation of Aminoguanidines **21**–**22** and **26**

A mixture of the appropriate cyanamide potassium salt **13**, **14** or **18** (7 mmol) and hydrazine monohydrochloride (0.48 g, 7 mmol) in anhydrous toluene (14 mL) was refluxed with stirring for 3–9 h. After cooling to room temperature, the reaction mixture was left in the freezer overnight. The precipitate was collected by filtration, dried and suspended in water (9 mL). The solid was separated and crystallized from ethanol (**21**–**22**) or *p*-dioxane (**26**). In this manner, the following aminoguanidines were obtained.

*1-Amino-2-{4-chloro-5-methyl-2-[(naphthalen-1-ylmethyl)thio]benzenesulfonyl}guanidine* (**21**). Starting from *N*-{4-chloro-5-methyl-2-[(naphthalen-1-ylmethyl)thio]benzenesulfonyl}cyanamide monopotassium salt **13** (3.09 g), the title compound **21** was obtained (2.55 g, 80%) mp 217–219 °C; IR (KBr)ν_max_ 3453, 3348, 3217 (NH), 2925, 2853 (C-H), 1385, 1133 (SO_2_) cm^−1^; ^1^H-NMR (500 MHz, DMSO-*d*_6_) δ 2.31 (s, 3H, CH_3_), 4.44 (s, 2H, C-NH_2_), 4.74 (s, 2H, SCH_2_), 6.93 (s, 2H, NNH_2_), 7.46 (t, 1H, arom), 7.51 (s, 1H, H-3, arom), 7.57–7.59 (m, 3H, arom), 7.84 (s, 1H, H-6, arom), 7.87 (d, *J* = 8.3 Hz, 1H, arom), 7.95 (d, *J* = 7.8 Hz, 1H, arom), 8.23 (d, *J* = 8.3 Hz, 1H, arom), 8.38 (s, 1H, NH) ppm; ^13^C-NMR (125 MHz, DMSO-*d*_6_) δ 19.7, 35.2, 124.9, 126.1, 126.6, 126.9, 128.6, 128.8, 129.2, 131.1, 132.1, 132.3, 132.8, 134.1, 136.6, 137.3, 140.4, 155.9, 157.6 ppm; anal. C 52.20, H 4.30, N 12.61% calcd. for C_19_H_19_ClN_4_O_2_S_2_, C 52.47, H 4.40, N 12.88%.

*1-Amino-2-{4-chloro-5-methyl-2-[(3,5-dimethylisoxazol-4-ylmethyl)thio]benzenesulfonyl}guanidine* (**22**). Starting from *N*-{4-chloro-5-methyl-2-[(3,5-dimethylisoxazol-4-ylmethyl)thio]benzenesulfonyl}cyanamide monopotassium salt **14** (2.87 g), the title compound **22** was obtained (2.34 g, 84%) mp 204–206 °C; IR (KBr)ν_max_ 3479, 3345, 3341 (NH); 2926, 2851 (C–H); 1268, 1131 (SO_2_) cm^−1^; ^1^H-NMR (500 MHz, DMSO-*d*_6_) δ 2.21 (s, 3H, CH_3_), 2.30–2.31 (m, 6H, 2×CH_3_), 4.08 (s, 2H, SCH_2_), 4.49 (s, 2H, CNH_2_), 6.97 (s, 2H, NNH_2_), 7.43 (s, 1H, H-3, arom), 7.82 (s,1H, H-6, arom), 8.39 (s, 1H, NH) ppm; ^13^C-NMR (125 MHz, DMSO-*d*_6_) δ 10.4, 11.3, 19.7, 25.3, 109.7, 129.7, 131.0, 133.0, 135.1, 136.6, 141.8, 159.4, 160.3, 167.3 ppm; anal. C 41.88, H 4.56, N 17.62% calcd. for C_14_H_18_ClN_5_O_3_S_2_, C 41.63, H 4.49, N 17.34%.

*1-Amino-2-(2-benzylthio-4-chloro-5-methoxyphenylcarbamoylbenzenesulfonyl)guanidine* (**26**). Starting from *N*-(2-benzylthio-4-chloro-5-methoxyphenylcarbamoylbenzenesulfonyl)cyanamide monopotassium salt **18** (3.68 g), the title compound **26** was obtained (2.52 g, 70%) mp 238–240 °C; IR (KBr) ν_max_ 3312, 3212 (NH), 2921, 2851 (C–H), 1678 (C=O), 1541, 1510 (C=C, C=N), 1312, 1134 (SO_2_) cm^−1^; ^1^H-NMR (500 MHz, DMSO-*d*_6_) δ 3.75 (s, 3H, OCH_3_), 4.41 (s, 2H, SCH_2_), 4.54 (s, 2H, NH_2_), 6.92–7.03 (m, 4H, arom), 7.29–7.41 (m, 3H, arom), 7.48–7.64 (m, 5H, arom), 7.97 (m, 1H, arom), 8.52 (s, 1H, NH), 10.43 (s, 1H, NHCO) ppm; anal. C 50.56, H 4.15, N 13.15 calcd. for C_22_H_22_ClN_5_O_4_S_2_, C 50.81, H 4.26, N 13.47%.

#### 3.2.2. Procedures for the Preparation of 4-Chloro-2-(R^2^-methylthio)-5-R^1^-*N*-(5-aryl-1,2,4-triazin-3-yl)benzenesulfonamides **27**–**60**

A mixture of the appropriate aminoguanidine **19**–**26** (1 mmol) and phenylglyoxal hydrate (1 mmol) in glacial acetic acid (2.5 mL) was refluxed with stirring for 24–45 h. The solid of the compound was obtained either after stirring overnight at room temperature, or after additional stirring on an ice bath for 12 h (compds. **30**–**31**, **42**–**44**, **47**). The formed precipitate was collected by filtration, washed with glacial acetic acid (2 × 1 mL) and dried. The final products **27**–**60** were purified as described below.

*2-Benzylthio-4-chloro-N-[5-(3-fluorophenyl)-1,2,4-triazin-3-yl]-5-methylbenzenesulfonamide* (**27**). Starting from **19** (0.385 g) and 3-fluorophenylglyoxal hydrate (0.170 g) after stirring for 33 h, the crude **27** (0.431 g) was obtained. The crude **27** was crystallized twice from EtOH and the title compound **27** (0.290 g, 58%) was isolated mp 216–218 °C; IR (KBr) ν_max_ 3430, 3196 (NH), 3074, 2921 (C–H), 1585, 1556 (C=C, C=N), 1302, 1159 (SO_2_) cm^−1^; ^1^H-NMR (200 MHz, DMSO-*d*_6_) δ 2.29 (s, 3H, CH_3_), 4.31 (s, 2H, SCH_2_), 7.09–7.10 (m, 3H, arom), 7.26–7.37 (m, 2H, arom), 7.50–7.67 (m, 4H, arom), 7.82–7.86 (m, 1H, arom), 8.13 (s, 1H, H-6, arom), 9.31 (s, 1H, triazine), 14.25 (brs, 1 H, SO_2_NH) ppm; ^13^C-NMR (50 MHz, DMSO-*d*_6_) δ 19.1, 36.2, 114.8, 115.3, 121.1, 125.1, 127.3, 128.3, 128.4, 129.1, 131.6, 131.7, 132.5, 132.7, 134.9, 135.1, 135.4, 136.5, 137.6, 160.1, 165.0 ppm; anal. C 55.32, H 3.36, N 11.36% calcd. for C_23_H_18_ClFN_4_O_2_S_2_, C 55.14, H 3.29, N 11.18%.

*2-Benzylthio-4-chloro-5-methyl-N-[5-(4-trifluoromethylphenyl)-1,2,4-triazin-3-yl]benzenesulfonamide* (**28**). Starting from **19** (0.385 g) and 4-trifluoromethylphenylglyoxal hydrate (0.220 g) after stirring for 33 h, the title compound **28** (0.298 g, 72%) was obtained without further purification mp 287–289 °C; IR (KBr) ν_max_ 3457, 3182 (NH), 3089, 2929 (C–H), 1585, 1560 (C=C, C=N), 1320, 1123 (SO_2_) cm^−1^; ^1^H-NMR (200 MHz, DMSO-*d*_6_) δ 2.37 (s, 3H, CH_3_), 4.32 (s, 2H, SCH_2_), 7.10–7.13 (m, 3H, arom), 7.28–7.30 (m, 2H, arom), 7.57 (s, 1H, H-3, arom), 7.92–7.96 (m, 2H, H-6, arom), 8.15–8.17 (m, 3H, arom), 9.42 (s, 1H, triazine), 14.10 (brs, 1 H, SO_2_NH) ppm; ^13^C-NMR (50 MHz, DMSO-*d*_6_) δ 19.1, 36.2, 121.3, 126.1, 126.2, 126.3, 127.3, 128.2, 128.5, 129.2, 129.4, 129.5, 132.3, 133.2, 135.7, 136.4, 136.7, 137.7, 137.8, 137.9 ppm; anal. C 52.45, H 3.41, N 10.28% calcd. for C_23_H_18_ClFN_4_O_2_S_2_, C 52.32, H 3.29, N 10.17%.

*2-Benzylthio-4-chloro-N-[5-(4-methoxyphenyl)-1,2,4-triazin-3-yl]-5-methylbenzenesulfonamide* (**29**). Starting from **19** (0.385 g) and 4-methoxyphenylglyoxal hydrate (0.182 g) after stirring for 31 h, the crude **29** (0.381 g) was obtained. The crude **29** was crystallized from MeCN and the title compound **29** (0.324 g, 63%) was isolated mp 232–234 °C; IR (KBr) ν_max_ 3188 (NH), 2918, 2831 (C–H), 1606, 1575 (C=C, C=N), 1533 (NH_def._), 1298, 1180 (SO_2_) cm^−1^; ^1^H-NMR (200 MHz, DMSO-*d*_6_) δ 2.40 (s, 3H, CH_3_), 3.88 (s, 3H, OCH_3_), 4.27 (s, 2H, SCH_2_), 7.05–7.17 (m, 4H, arom), 7.24–7.31 (m, 3H, arom), 7.48 (s, 1H, H-3, arom), 7.95 (d, 2H, *J* = 8.93 Hz, arom), 8.12 (s, 1H, H-6), 9.08 (s, 1H, triazine) ppm; anal. C 56.02, H 3.96, N 10.91% calcd. for C_24_H_21_ClN_4_O_3_S_2_, C 56.19, H 4.13, N 10.92%.

*2-Benzylthio-4-chloro-N-[5-(3-methoxyphenyl)-1,2,4-triazin-3-yl]5-methylbenzenesulfonamide* (**30**). Starting from **19** (0.385 g) and 3-methoxyphenylglyoxal hydrate (0.182 g) after stirring for 33 h, the title compound **30** (0.400 g, 78%) was obtained without further purification mp 198–200 °C; IR (KBr) ν_max_ 3199 (NH), 2934 (C–H), 1631, 1582 (C=C, C=N), 1561 (NH_def._), 1283, 1132 (SO_2_) cm^−1^; ^1^H-NMR (200 MHz, DMSO-*d*_6_) δ 2.28 (s, 3H, CH_3_), 3.81 (s, 3H, OCH_3_), 4.29 (s, 2H, SCH_2_), 7.10–7.13 (m, 3H, arom), 7.26–7.32 (m, 3H, arom), 7.42–7.52 (m, 4H, H-3, arom.), 8.08 (s, 1H, H-6, arom), 9.27 (s, 1H, triazine) ppm; anal. C 56.13, H 4.01, N 10.96% calcd. for C_24_H_21_ClN_4_O_3_S_2_, C 56.19, H 4.13, N 10.92%.

*2-Benzylthio-4-chloro-N-[5-(3,4-dimethoxyphenyl)-1,2,4-triazin-3-yl]-5-methylbenzenesulfonamide* (**31**). Starting from **19** (0.385 g) and 3,4-dimethoxyphenylglyoxal hydrate (0.212 g) after stirring for 32 h, the title compound **31** (0.443 g, 82%) was obtained without further purification mp 198–200 °C; IR (KBr) ν_max_ 3447, 3125 (NH), 3000, 2936 (C–H), 1615, 1554, 1519 (C=C, C=N), 1316, 1133 (SO_2_) cm^−1^; ^1^H-NMR (200 MHz, DMSO-*d*_6_) δ 2.35 (s, 3H, CH_3_), 3.80 (s, 3H, OCH_3_), 3.88 (s, 3H, OCH_3_), 4.28 (s, 2H, SCH_2_), 7.08–7.19 (m, 4H, arom), 7.28–7.32 (m, 2H, arom), 7.47 (s, 1H, H-3, arom), 7.56 (d, *J* = 1.91 Hz, 1H, arom), 7.67 (dd, *J_1_* = 8.50 Hz, *J_2_* = 2.00 Hz, 1H, arom), 8.08 (s, 1H, H-6, arom), 9.20 (s, 1H, triazine) ppm; anal. C 55.08, H 4.13, N 10.11% calcd. for C_25_H_23_ClN_4_O_4_S_2_, C 55.29, H 4.27, N 10.32%.

*2-Benzylthio-4-chloro-5-methyl-N-[5-(3,4,5-trimethoxyphenyl)-1,2,4-triazin-3-yl]benzenesulfonamide* (**32**). Starting from **19** (0.385 g) and 3,4,5-trimethoxyphenylglyoxal hydrate (0.242 g) after stirring for 33 h, the title compound **32** (0.525 g, 92%) was obtained without further purification mp 163–164 °C; IR (KBr) ν_max_ 3436 (NH), 3073, 2936 (C–H), 1594, 1550 (C=N, C=C), 1332, 1129 (SO_2_) cm^−1^; ^1^H-NMR (200 MHz, DMSO-*d*_6_) δ 2.28 (s, 3H_,_ CH_3_), 3.79 (s, 3H, OCH_3_), 3.85 (s, 6H, 2×OCH_3_), 4.32 (s, 2H, SCH_2_), 7.17–7.19 (m, 3H, arom), 7.31–7.35 (m, 4H, arom), 7.52 (s, 1H, H-3, arom), 8.04 (s, 1H, H-6, arom), 9.34 (brs, 1H, triazine), 14.25 (brs, 1H, SO_2_NH) ppm; ^13^C-NMR (50 MHz, DMSO-*d*_6_) δ: 19.1, 36.3, 56.7, 60.7, 107.1, 127.4, 127.7, 127.9, 128.5, 129.2, 132.0, 132.1, 135.4, 136.4, 137.3, 146.2, 153.6 ppm; anal. C 54.82, H 4.56, N 10.07% calcd. for C_26_H_25_ClN_4_O_5_S_2_, C 54.49, H 4.39, N 9.78%.

*4-Chloro-N-[5-(3-fluorophenyl)-1,2,4-triazin-3-yl]-5-methyl-2-[4-(trifluoromethyl)benzylthio]benzenesulfonamide* (**33**). Starting from **20** (0.451 g) and 3-fluorophenylglyoxal hydrate (0.170 g) after stirring for 33 h, the crude **33** (0.446 g) was obtained. The crude **33** was purified by heating under reflux with EtOH and the title compound **33** (0.330 g, 58%) was isolated mp 212–214 °C; IR (KBr) ν_max_ 3436, 3186 (NH), 3092, 2923, 2851 (C–H), 1589, 1554 (C=C, C=N), 1326, 1126 (SO_2_) cm^−1^; ^1^H-NMR (500 MHz, DMSO-*d*_6_) δ 2.35 (s, 3H, CH_3_), 4.42 (s, 2H, SCH_2_), 7.39 (d, *J* = 8.30 Hz, 2H, arom), 7.43–7.53 (m, 4H, arom), 7.54–7.65 (m, 2H, arom), 7.76 (d, *J* = 7.80 Hz, 1H, arom), 8.08 (s, 1H, H-6, arom), 9.28 (brs, 1H, triazine) ppm; anal. C 50.71, H 3.11, N 9.89% calcd. for C_24_H_17_ClF_4_N_4_O_2_S_2_, C 50.66, H 3.01, N 9.85%.

*4-Chloro-5-methyl-2-[4-(trifluoromethyl)benzylthio]-N-[5-(4-trifluoromethylphenyl)-1,2,4-triazin-3-yl]-benzenesulfonamide* (**34**). Starting from **20** (0.451 g) and 4-trifluoromethylphenylglyoxal hydrate (0.220 g) after stirring for 33 h, the crude **34** (0.447 g) was obtained. The crude **34** was purified by heating under reflux with EtOH and the title compound **34** (0.331 g, 53%) was isolated mp 256–259 °C; IR (KBr) ν_max_ 3444 (NH), 3078, 2925 (C–H), 1581, 1556 (C=C, C=N), 1325, 1131 (SO_2_) cm^−1^; ^1^H-NMR (500 MHz, DMSO-*d*_6_) δ 2.33 (s, 3H, CH_3_), 4.42 (s, 2H, SCH_2_), 7.40 (d, 2H, *J* = 7.80 Hz, arom), 7.49 (d, 2H, *J* = 8.30 Hz, arom), 7.60 (s, 1H, H-3, arom), 7.89 (d, 2H, *J* = 8.30 Hz, arom), 8.08 (d, *J* = 8.30 Hz, 2H, arom), 8.13 (s, 1H, H-6, arom), 9.39 (brs, 1H, triazine) ppm; anal. C 48.66, H 3.00, N 9.12% calcd. for C_25_H_17_ClF_6_N_4_O_2_S_2_, C 48.51, H 2.77, N 9.05%.

*4-Chloro-N-[5-(4-methoxyphenyl)-1,2,4-triazin-3-yl]-5-methyl-2-[4-(trifluoromethyl)benzylthio]benzene-sulfonamide* (**35**). Starting from **20** (0.451 g) and 4-methoxyphenylglyoxal hydrate (0.182 g) after stirring for 29 h, the crude **35** (0.462 g) was obtained. The crude **35** was purified by heating under reflux with EtOH and the title compound **35** (0.387 g, 66%) was isolated mp 255–258 °C; IR (KBr) ν_max_ 3435 (NH), 3084, 2931 (C–H), 1579, 1536 (C=C, C=N), 1299, 1125 (SO_2_) cm^−1^; ^1^H-NMR (500 MHz, DMSO-*d*_6_) δ 2.37 (s, 3H, CH_3_), 3.86 (s, 3H, OCH_3_), 4.39 (s, 2H, SCH_2_), 7.04 (d, 2H, *J* = 8.8 Hz, arom), 7.44 (d, 2H, arom), 7.48-7.58 (m, 3H, arom), 7.89 (d, 2H, *J* = 7.8 Hz, arom), 8.10 (s, 1H, H-6, arom), 9.04 (brs, 1H, triazine), 14.51 (brs, 1H, SO_2_NH) ppm; ^13^C-NMR (125 MHz, DMSO-*d*_6_) δ 19.7, 35.8, 56.5, 88.9, 115.4, 123.6, 124.9, 125.6, 128.1, 128.3, 130.3, 132.9, 134.8, 142.4 ppm; anal. C 51.75, H 3.49, N 9.68% calcd. for C_25_H_20_ClF_3_N_4_O_3_S_2_, C 51.68, H 3.47, N 9.64%.

*4-Chloro-N-[5-(3-fluorophenyl)-1,2,4-triazin-3-yl]-5-methyl-2-[(naphthalene-1-yl)methylthio]benzene-sulfonamide* (**36**). Starting from **21** (0.435 g) and 3-fluorophenylglyoxal hydrate (0.170 g) after stirring for 43 h, the crude **36** (0.417 g) was obtained. The crude **36** was purified by heating under reflux with EtOH and the title compound **36** (0.277 g, 50%) was isolated mp 234–237 °C; IR (KBr) ν_max_ 3432 (NH), 3052, 2922 (C–H), 1552, 1524 (C=C, C=N), 1442, 1168 (SO_2_) cm^−1^; ^1^H-NMR (500 MHz, DMSO-*d*_6_) δ 2.40 (s, 3H, CH_3_), 4.75 (s, 2H, SCH_2_), 7.26 (t, 1H, arom), 7.42–7.57 (m, 5H, arom), 7.58–7.64 (m, 1H, arom), 7.68 (s, 1H, H-3, arom), 7.74 (d, *J* = 8.30 Hz, 1H, arom), 7.81–7.82 (m, 1H, arom), 7.86–7.89 (m, 1H, arom), 8.08–8.12 (m, 2H, arom), 9.20 (brs, 1H, triazine) ppm; anal. C 58.53, H 3.45, N 9.95% calcd. for C_27_H_20_ClFN_4_O_2_S_2_, C 58.85, H 3.66, N 10.17%.

*4-Chloro-5-methyl-2-[(naphthalene-1-yl)methylthio]-N-[5-(4-trifluoromethylphenyl)-1,2,4-triazin-3-yl]-benzenesulfonamide* (**37**). Starting from **21** (0.435 g) and 4-trifluoromethylphenylglyoxal hydrate (0.220 g) after stirring for 30 h, the crude **37** (0.526 g) was obtained. The crude **37** was purified by heating under reflux with EtOH and the title compound **37** (0.417 g, 69%) was isolated: mp 247–251 °C; IR (KBr) ν_max_ 3438 (NH), 3080, 2927 (C–H), 1580, 1554 (C=C, C=N), 1323, 1162 (SO_2_) cm^−1^; ^1^H-NMR (500 MHz, DMSO-*d*_6_) δ 2.38 (s, 3H, CH_3_), 4.76 (s, 2H, SCH_2_), 7.28 (t, 1H, arom), 7.39–7.57 (m, 3H, arom), 7.67 (s, 1H, H-3, arom), 7.76 (d, *J* = 7.8 Hz,1H, arom), 7.82–8.02 (m, 3H, arom), 8.02–8.28 (m, 4H, arom), 9.30 (brs, 1H, triazine) ppm; ^13^C-NMR (125 MHz, DMSO-*d*_6_) δ 19.6, 34.9, 124.8, 125.9, 126.6, 126.9, 128.6, 128.8, 129.1, 129.1, 130.0, 131.9, 132.2, 132.7, 133.5, 133.9, 137.0 ppm; anal. C 55.63, H 3.20, N 8.99% calcd. for C_28_H_20_ClF_3_N_4_O_2_S_2_, C 55.95, H 3.35, N 9.32%.

*4-Chloro-N-[5-(4-methoxyphenyl)-1,2,4-triazin-3-yl]-5-methyl-2-[(naphthalene-1-yl)methylthio]benzene-sulfonamide* (**38**). Starting from **21** (0.435 g) and 4-methoxyphenylglyoxal hydrate (0.182 g) after stirring for 40 h, the crude **38** (0.461 g) was obtained. The crude **38** was purified by heating under reflux with EtOH and the title compound **38** (0.389 g, 75%) was isolated mp 262–265 °C; IR (KBr) ν_max_ 3450 (NH), 3060, 2925 (C–H), 1607, 1541 (C=C, C=N), 1264, 1176 (SO_2_) cm^−1^; ^1^H-NMR (500 MHz, DMSO-*d*_6_) δ 2.42 (s, 3H, CH_3_), 3.88 (s, 3H, OCH_3_), 4.73 (s, 2H, SCH_2_), 7.06 (d, *J* = 8.8 Hz, 2H, arom), 7.30 (t, 1H, arom), 7.44–7.56 (m, 3H, arom), 7.60 (s, 1H, H-3, arom), 7.78 (d, *J* = 8.3 Hz, 1H, arom), 7.84–8.02 (m, 3H, arom), 8.09 (d, *J* = 7.8 Hz, 1H, arom), 8.13 (s, 1H, H-6 arom), 9.00 (brs, 1H, triazine), 14.39 (brs, 1H, SO_2_NH) ppm; ^13^C-NMR (125 MHz, DMSO-*d_6_*): δ 18.2, 37.1, 55.2, 110.9, 113.7, 115.4, 115.8, 117.7, 122.0, 122.7, 124.9, 125.9, 126.6, 128.4, 128.7, 130.3, 131.5, 132.7, 133.1, 133.4, 136.3, 136.5, 137.8, 142.7, 150.9, 168.8 ppm; anal. C 59.56, H 4.09, N 9.89% calcd. for C_28_H_23_ClN_4_O_3_S_2_, C 59.72, H 4.12, N 9.95%.

*4-Chloro-2-[(3,5-dimethylizoxazol-4-yl)methylthio]-N-[5-(3-fluorophenyl)-1,2,4-triazin-3-yl]-5-methyl-benzenesulfonamide* (**39**). Starting from **22** (0.404 g) and 3-fluorophenylglyoxal hydrate (0.170 g) after stirring for 25 h, the crude **39** (0.380 g) was obtained. The crude **39** was purified by heating under reflux with EtOH and the title compound **39** (0.178 g, 33%) was isolated mp 208–211 °C; IR (KBr) ν_max_ 3435 (NH), 3071, 2963, 2925, 2854, 2737 (C–H), 1551, 1514 (C=C, C=N), 1326, 1156 (SO_2_) cm^−1^; ^1^H-NMR (500 MHz, DMSO-*d*_6_) δ 2.06 (s, 3H, CH_3_), 2.11 (s, 3H, CH_3_), 2.41 (s, 3H, CH_3_), 4.10 (s, 2H, SCH_2_), 7.34–7.46 (s, 1H, arom), 7.48–7.66 (m, 2H, arom), 7.72 (s, 1H, H-3, arom), 7.77 (d, *J* = 7.8 Hz, 1H, arom), 8.09 (s, 1H, H-6, arom), 9.24 (bs, 1H, triazine) ppm; ^13^C-NMR (125 MHz, DMSO-*d*_6_) δ 10.3, 11.2, 19.6, 25.6, 110.3, 115.3, 115.5, 125.6, 130.7, 131.9, 132.0, 133.9, 135.3, 135.3, 135.4, 159.9, 161.9, 163.9, 166.7 ppm; anal. C 51.05, H 3.75, N 13.70% calcd. for C_22_H_19_ClFN_5_O_3_S_2_, C 50.81, H 3.68, N 13.47%.

*4-Chloro-2-[(3,5-dimethylizoxazol-4-yl)methylthio]-5-methyl-N-[5-(4-trifluoromethylphenyl)-1,2,4-triazin-3-yl]benzenesulfonamide* (**40**). Starting from **22** (0.404 g) and 4-trifluoromethylphenylglyoxal hydrate (0.220 g) after stirring for 45 h, the crude **40** (0.516 g) was obtained. The crude **40** was purified by heating under reflux with EtOH and the title compound **40** (0.250 g, 43%) was isolated mp 218–221 °C; IR (KBr) ν_max_ 3434 (NH), 3072, 2754 (C-H), 1550, 1519 (C=C,C=N), 1325, 1156 (SO_2_) cm^−1^; ^1^H-NMR (500 MHz, DMSO-*d*_6_) δ 2.08 (s, 3H, CH_3_), 2.14 (s, 3H, CH_3_), 2.39 (s, 3H, CH_3_), 4.11 (s, 2H, SCH_2_), 7.67 (s, 1H, H-6, arom), 7.90 (d, *J* = 8.3 Hz, 2H, arom), 8.12 (d, 2H, arom), 8.15 (s, 1H, H-6, arom), 9.36 (brs, 1H, triazine) ppm; ^13^C-NMR (125 MHz, DMSO-*d*_6_) δ 10.3, 11.2, 19.6, 25.6, 110.0, 121.1, 123.3, 125.4, 126.5, 126.6, 127.6, 129.9, 130.4, 133.7, 135.2, 137.0, 160.0, 166.9 ppm; anal. C 51.15, H 3.55, N 10.10% calcd. for C_23_H_19_ClF_3_N_5_O_3_S_2_ , C 50.84, H 3.20, N 9.88%.

*4-Chloro-2-[(3,5-dimethylizoxazol-4-yl)methylthio]-N-[5-(4-methoxyphenyl)-1,2,4-triazin-3-yl]-5-methyl-benzenesulfonamide* (**41**). Starting from **22** (0.404 g) and 4-methoxyphenylglyoxal hydrate (0.182 g) after stirring for 24 h, the crude **41** (0.435 g) was obtained. The crude **41** was purified by heating under reflux with EtOH and the title compound **41** (0.364 g, 68%) was isolated mp 224–227 °C; IR (KBr) ν_max_ 3418, 3218 (NH), 3071, 2925, 2847, (C–H), 1577, 1541 (C=C, C=N), 1267, 1157 (SO_2_) cm^−1^; ^1^H-NMR (500 MHz, DMSO-*d*_6_) δ 2.08 (s, 3H, CH_3_), 2.13 (s, 3H, CH_3_), 2.43 (s, 3H, CH_3_), 3.87 (s, 3H, OCH_3_), 4.08 (s, 2H, SCH_2_), 7.03 (d, *J* = 8.8 Hz, 2H, arom), 7.62 (s, 1H, H-3, arom), 7.86 (d, *J* = 8.30 Hz, 2H, arom), 8.10 (s, 1H, H-6, arom), 9.03 (brs, 1H, triazine), 14.40 (brs, 1H, SO_2_NH) ppm; ^13^C-NMR (125 MHz, DMSO-*d*_6_) δ 10.3, 11.2, 19.7, 25.5, 56.5, 110.2, 115.4, 125.0, 130.1, 133.5, 160.0, 166.8 ppm; anal. C 52.03, H 4.20, N 13.39% calcd. for C_23_H_22_ClN_5_O_4_S_2_, C 51.92, H 4.17, N 13.16%.

*4-Benzylthio-2-chloro-5-{N-[5-(4-methoxyphenyl)-1,2,4-triazin-3-yl]sulfamoyl}-N-phenylbenzamide* (**42**). Starting from **23** (0.451 g) and 4-methoxyphenylglyoxal hydrate (0.182 g) after stirring for 31 h, the crude **42** (0.486 g) was obtained. The crude **42** was crystallized from MeCN and the title compound **42** (0.383 g, 67%) was isolated mp 283–286 °C; IR (KBr) ν_max_ 3409, 3323 (NH), 2923, 2843 (C–H), 1664 (C=O), 1604, 1576 (C=C, C=N), 1535 (NH_def._), 1315, 1174 (SO_2_) cm^−1^; ^1^H-NMR (200 MHz, DMSO-*d*_6_) δ 3.77 (s, 3H, OCH_3_), 4.38 (s, 2H, SCH_2_), 7.04 (d, *J* = 9.09 Hz, 2H, arom), 7.14–7.22 (m, 4H, arom), 7.32–7.42 (m, 4H, arom), 7.63 (s, 1H, H-3, arom), 7.73 (d, *J* = 7.67 Hz, 2H, arom), 7.94 (d, *J* = 8.92 Hz, 2H, arom), 8.26 (s, 1H, H-6, arom), 9.09 (s, 1H, triazine), 10.63 (s, 1H, NHCO), 14.50 (s, 1H, SO_2_NH) ppm; anal. C 58.03, H 2.99, N 11.18% calcd. for C_30_H_24_ClN_5_O_4_S_2_, C 58.29, H 3.19, N 11.33%.

*4-Benzylthio-2-chloro-5-{N-[5-(3-methoxyphenyl)-1,2,4-triazin-3-yl]sulfamoyl}-N-phenylbenzamide* (**43**). Starting from **23** (0.451 g) and 3-methoxyphenylglyoxal hydrate (0.182 g) after stirring for 33 h, the crude **43** (0.453 g) was obtained. The crude **43** was purified by heating under reflux with EtOH and the title compound **43** (0.399 g, 71%) was isolated mp 270–272 °C; IR (KBr) ν_max_ 3436, 3326 (NH), 2935 (C–H), 1681 (C=O), 1603, 1554 (C=N, C=C), 1314, 1137 (SO_2_) cm^−1^; ^1^H-NMR (500 MHz, DMSO-*d*_6_) δ 3.73 (s, 3H, OCH_3_), 4.39 (s, 2H, SCH_2_), 7.11–7.20 (m, 5H, arom), 7.34–7.42 (m, 6H, arom), 7.63–7.68 (m, 4H, H-3, arom), 8.21 (s, 1H, H-6, arom), 9.23 (s, 1H, triazine), 10.54 (s, 1H, NHCO) ppm; anal. C 58.49, H 3.30, N 11.42% calcd. for C_30_H_24_ClN_5_O_4_S_2_, C 58.29, H 3.19, N 11.33%.

*4-Benzylthio-2-chloro-5-{N-[5-(3,4-dimethoxyphenyl)-1,2,4-triazin-3-yl]sulfamoyl}-N-phenylbenzamide* (**44**). Starting from **23** (0.451 g) and 3,4-dimethoxyphenylglyoxal hydrate (0.212 g) after stirring for 32 h, the crude **44** (0.500 g) was obtained. The crude **44** was crystallized from EtOH and the title compound **44** (0.407 g, 68%) was isolated mp 252–253 °C; IR (KBr) ν_max_ 3419, 3328 (NH), 2933 (C–H), 1651 (C=O), 1600, 1540 (C=N, C=C), 1318, 1133 (SO_2_) cm^−1^; ^1^H-NMR (200 MHz, DMSO-*d*_6_) δ 3.72 (s, 3H, OCH_3_), 3.78 (s, 3H, OCH_3_), 4.40 (s, 2H, SCH_2_), 6.99–7.24 (m, 6H, arom), 7.32–7,39 (m, 3H, arom), 7.61–7.71 (m, 5H, arom), 8.25 (s, 1H, arom), 9.17 (s, 1H, triazine), 10.57 (s, 1H, NHCO) ppm; anal. C 57.50, H 4.03, N 10.81% calcd. for C_31_H_26_ClN_5_O_5_S_2_, C 57.45, H 4.04, N 10.81%.

*4-Benzylthio-2-chloro-N-(4-chlorophenyl)-5-{N-[5-(3-fluorophenyl)-1,2,4-triazin-3-yl]sulfamoyl}benzamide* (**45**). Starting from **24** (0.524 g) and 3-fluorophenylglyoxal hydrate (0.170 g) after stirring for 33 h, the title compound **45** (0.470 g, 73%) was obtained without further purification mp 280–282 °C; IR (KBr) ν_max_ 3429, 3276 (NH), 3065, 2923 (C–H), 1657 (C=O), 1558, 1527 (C=N, C=C), 1310, 1166 (SO_2_) cm^−1^; ^1^H-NMR (200 MHz, DMSO-*d*_6_) δ 4.42 (s, 2H, SCH_2_), 7.17–7.36 (m, 3H, arom), 7.43–7.63 (m, 6H, arom), 7.66–7.75 (m, 4H, arom), 7.93 (d, *J* = 9.61 Hz, 1H, arom), 8.25 (s, 1H, H-6, arom), 9.28 (s, 1H, triazine), 10.72 (s, 1H, CONH), 14.45 (brs, 1H, SO_2_NH) ppm; anal. C 54.58, H 3.22, N 11.01% calcd. for C_29_H_20_Cl_2_FN_5_O_3_S_2_, C 54.36, H 3.15, N 10.93%.

*4-Benzylthio-2-chloro-N-(4-chlorophenyl)-5-{N-[5-(4-trifluoromethylphenyl)-1,2,4-triazin-3-yl]sulfamoyl}-benzamide* (**46**). Starting from **24** (0.524 g) and 4-trifluoromethylphenylglyoxal hydrate (0.220 g) after stirring for 33 h, the crude **46** (0.561 g) was obtained. The crude **46** was purified by heating under reflux with MeCN and the title compound **46** (0.553 g, 80%) was isolated mp 273–275 °C; IR (KBr) ν_max_ 3290 (NH), 3062, 2922 (C–H), 1658 (C=O), 1560, 1529 (C=C, C=N), 1325, 1165 (SO_2_) cm^−1^; ^1^H-NMR (200 MHz, DMSO-*d*_6_) δ 4.43 (s, 2H, SCH_2_), 7.16–7.28 (m, 3H, arom), 7.34–7.46 (m, 6H, H-3, arom), 7.73–7.77 (m, 3H, arom), 7.85–7.89 (m, 2H, arom), 8.09–8.13 (m, 2H, arom), 8.30 (s, 1H, H-6, arom), 9.36 (s, 1H, triazine), 10.80 (s, 1H, NHCO) ppm; ^13^C-NMR (50 MHz, DMSO-*d*_6_) δ 35.9, 121.4, 126.3, 126.4, 126.6, 127.5, 128.0, 128.3, 128.6, 129.0, 129.2, 129.3, 129.6, 130.9, 132.2, 132.8, 133.4, 134.3, 136.2, 136.4, 137.9, 140.8, 155.1, 163.9 ppm; anal. C 52.36, H 3.15, N 10.23% calcd. for C_30_H_20_Cl_2_F_3_N_5_O_3_S_2_, C 52.18, H 2.92, N 10.14%.

*4-Benzylthio-2-chloro-N-(4-chlorophenyl)-5-{N-[5-(4-methoxyphenyl)-1,2,4-triazin-3-yl]sulfamoyl}benzamide* (**47**). Starting from **24** (0.524 g) and 4-methoxyphenylglyoxal hydrate (0.182 g) after stirring for 30 h, the crude **47** (0.502 g) was obtained. The crude **47** was purified by heating under reflux with MeCN and the title compound **47** (0.363 g, 56%) was isolated mp 282–284 °C; IR (KBr) ν_max_ 3411 (NH), 2925, 2843 (C–H), 1663 (C=O), 1604, 1577 (C=N, C=C), 1537 (NH_def._), 1314, 1174 (SO_2_) cm^−1^; ^1^H-NMR (200 MHz, DMSO-*d*_6_) δ 3.79 (s, 3H, OCH_3_), 4.39 (s, 2H, SCH_2_), 7.03 (d, *J* = 8.97 Hz, 2H, arom), 7.17–7.23 (m, 3H, arom), 7.32–7.37 (m, 2H, arom), 7.44 (d, *J* = 8.88 Hz, 2H, arom), 7.64 (s, 1H, H-3, arom), 7.75 (d, *J* = 8.92 Hz, 2H, arom), 7.94 (d, 2H, arom), 8.27 (s, 1H, H-6, arom), 9.09 (s, 1H, triazine), 10.74 (s, 1H, NHCO) ppm; anal. C 55.01, H 3.46, N 10.58% calcd. for C_30_H_23_Cl_2_N_5_O_4_S_2_, C 55.22, H 3.55, N 10.87%.

*4-Benzylthio-2-chloro-N-(4-chlorophenyl)-5-{N-[5-(3-methoxyphenyl)-1,2,4-triazin-3-yl]sulfamoyl}-benzamide* (**48**). Starting from **24** (0.524 g) and 3-methoxyphenylglyoxal hydrate (0.182 g) after stirring for 33 h, the crude **48** (0.358 g) was obtained. The crude **48** was purified by heating under reflux with MeCN and the title compound **48** (0.356 g, 56%) was isolated mp 256–258 °C; IR (KBr) ν_max_ 3318 (NH), 3088, 2921 (C–H), 1684 (C=O), 1597, 1553 (C=C, C=N), 1311, 1162 (SO_2_) cm^−1^; ^1^H-NMR (200 MHz, DMSO-*d*_6_) δ 3.76 (s, 3H, OCH_3_), 4.42 (s, 2H, SCH_2_), 7.20–7.38 (m, 4H, arom), 7.43–7.46 (m, 6H, arom), 7.67–7.75 (m, 4H, arom), 8.26 (s, 1H, H-6, arom), 9.27 (s, 1H, triazine), 10.70 (s, 1H, NHCO) ppm; anal. C 55.36, H 3.60, N 10.80% calcd. for C_30_H_23_Cl_2_N_5_O_4_S_2_, C 55.22, H 3.55, N 10.87%.

*4-Benzylthio-2-chloro-N-(4-chlorophenyl)-5-{N-[5-(3,4-dimethoxyphenyl)-1,2,4-triazin-3-yl]sulfamoyl}-benzamide* (**49**). Starting from **24** (0.524 g) and 3,4-dimethoxyphenylglyoxal hydrate (0.212 g) after stirring for 33 h, the crude **49** (0.570 g) was obtained. The crude **49** was crystallized from dry *p*-dioxane and the title compound **49** (0.264 g, 39%) was isolated: mp: 257–259 °C; IR (KBr) ν_max_ 3435, 3327 (NH), 2921 (C–H), 1656 (C=O), 1600, 1544 (C=C, C=N), 1318, 1132 (SO_2_) cm^−1^; ^1^H-NMR (200 MHz, DMSO-*d*_6_) δ 3.76 (s, 3H, OCH_3_), 3.78 (s, 3H, OCH_3_), 4.41 (s, 2H, SCH_2_), 6.99–7.04 (m, 1H, arom), 7.23–7.26 (m, 3H, arom), 7.39–7.45 (m, 4H, arom), 7.63–7.72 (m, 3H, arom), 8.27 (s, 1H, H-6, arom), 9.19 (s, 1H, triazine), 10.69 (s, 1H, CONH) ppm; anal. C 54.60, H 3.75, N 10.27% calcd. for C_31_H_25_Cl_2_N_5_O_5_S_2_, C 54.55, H 3.69, N 10.26%.

*4-Benzylthio-2-chloro-N-(4-chlorophenyl)-5-{N-[5-(3,4,5-trimethoxyphenyl)-1,2,4-triazin-3-yl]sulfamoyl}-benzamide* (**50**). Starting from **24** (0.524 g) and 3,4,5-trimethoxyphenylglyoxal hydrate (0.242 g) after stirring for 33 h, the crude **50** (0.385 g) was obtained. The crude **50** was crystallized from dry *p*-dioxane and the title compound **50** (0.182 g, 47%) was isolated mp 260–262 °C; IR (KBr) ν_max_ 3301, 3102 (NH), 2925, 2853 (C–H), 1682 (C=O), 1596, 1552 (C=C, C=N), 1333, 1131 (SO_2_) cm^−1^; ^1^H-NMR (200 MHz, DMSO-*d*_6_) δ 3.69 (s, 3H, OCH_3_), 3.79 (s, 6H, 2×OCH_3_), 4.43 (s, 2H, SCH_2_), 7.24–7.46 (m, 8H, arom), 7.64–7.73 (m, 4H, arom), 8.22 (s, 1H, H-6, arom), 9.31 (s, 1H, triazine), 10.66 (s, 1H, CONH), 14.5 (brs, 1H, SO_2_NH) ppm; anal. C 53.58, H 3.56, N 9.49% calcd. for C_32_H_27_Cl_2_N_5_O_6_S_2_, C 53.93, H 3.82, N 9.83%.

*4-Benzylthio-2-chloro-5-{N-[5-(3-fluorophenyl)-1,2,4-triazin-3-yl]sulfamoyl}-N-(4-methylphenyl)benzamide* (**51**). Starting from **25** (0.504 g) and 3-fluorophenylglyoxal hydrate (0.170 g) after stirring for 33 h, the crude **51** (0.470 g) was obtained. The crude **51** was purified by heating under reflux with EtOH and the title compound **51** (0.322 g, 52%) was isolated mp 266–268 °C; IR (KBr) ν_max_ 3427, 3278 (NH), 3064, 2921 (C–H), 1654, 1528 (C=C, C=N), 1316, 1165 (SO_2_) cm^−1^; ^1^H-NMR (200 MHz, DMSO-*d*_6_) δ 2.30 (s, 3H, CH_3_), 4.41 (s, 2H, SCH_2_), 7.17–7.20 (m, 4H, arom), 7.33–7.35 (m, 2H, H-3, arom), 7.45–7.68 (m, 7H, arom), 7.95 (d, *J* = 9.9 Hz, 1H, arom), 8.23 (s, 1H, H-6, arom), 9.28 (s, 1H, triazine), 10.49 (s, 1H, NHCO), 14.5 (brs, 1H, SO_2_NH) ppm; anal. C 58.22, H 3.80, N 11.31% calcd. for C_30_H_23_ClFN_5_O_3_S_2_, C 58.09, H 3.74, N 11.29%.

*4-Benzylthio-2-chloro-N-(4-methylphenyl)-5-{N-[5-(4-trifluoromethylphenyl)-1,2,4-triazin-3-yl]sulfamoyl}-benzamide* (**52**). Starting from **25** (0.504 g) and 4-trifluoromethylphenylglyoxal hydrate (0.220 g) after stirring for 33 h, the crude **52** (0.560 g) was obtained. The crude **52** was purified by heating under reflux with EtOH and the title compound **52** (0.370 g, 55%) was isolated mp 277–279 °C; IR (KBr) ν_max_ 3343, 3347 (NH), 3091, 2920 (C–H), 1668 (C=O)_,_ 1558, 1517 (C=C, C=N), 1316, 1120 (SO_2_) cm^−1^; ^1^H-NMR (200 MHz, DMSO-*d*_6_) δ 2.31 (s, 3H, CH_3_), 4.43 (s, 2H, SCH_2_), 7.16–7.21 (m, 5H, arom), 7.34–7.36 (m, 2H, arom), 7.62 (d, *J* = 8.0 Hz, 2H, arom), 7.72 (s, 1H, H-3, arom), 7.89 (d, *J* = 8.2 Hz, 2H, arom), 8.11 (d, 2H, arom), 8.28 (s, 1H, H-6, arom), 9.36 (s, 1H, triazine), 10.61 (s, 1H, NHCO), 14.13 (brs, 1H, SO_2_NH) ppm; ^13^C-NMR (50 MHz, DMSO-*d*_6_) δ 20.8, 35.9, 119.8, 126.3, 126.4, 127.5, 128.3, 128.5, 128.6, 129.2, 129.5, 129.6, 130.9, 132.6, 133.4, 134.4, 136.2, 136.4, 136.5, 136.9, 140.5, 155.0, 163.6 ppm; anal. C 55.28, H 3.10, N 10.09% calcd. for C_31_H_23_ClF_3_N_5_O_3_S_2_, C 55.56, H 3.46, N 10.45%.

*4-Benzylthio-2-chloro-5-{N-[5-(4-methoxyphenyl)-1,2,4-triazin-3-yl]sulfamoyl}-N-(4-methylphenyl)-benzamide* (**53**). Starting from **25** (0.504 g) and 4-methoxyphenylglyoxal hydrate (0.182 g) after stirring for 33 h, the title compound **53** (0.490 g, 77%) was obtained without further purification mp 255–258 °C; IR (KBr) ν_max_ 3315 (NH), 3087, 2921, 2851 (C–H), 1675 (C=O), 1577, 1540 (C=C, C=N), 1315, 1162 (SO_2_) cm^−1^; ^1^H-NMR (200 MHz, DMSO-*d*_6_) δ 2.31 (s, 3H, CH_3_), 3.78 (s, 3H, OCH_3_), 4.40 (s, 2H, SCH_2_), 7.05 (d, *J* = 8.3 Hz, 2H, arom), 7.18–7.21 (m, 5H, arom), 7.34–7.36 (m, 2H, arom), 7.62–7.65 (m, 3H, H-3, arom), 7.95 (d, *J* = 8.3 Hz, 2H, arom), 8.27 (s, 1H, H-6, arom), 9.10 (s, 1H, triazine), 10.57 (s, 1H, NHCO), 14.5 (brs, 1H, SO_2_NH ) ppm; ^13^C-NMR (50 MHz, DMSO-*d*_6_) δ 20.8, 35.9, 55.7, 115.2, 119.9, 124.5, 127.5, 127.9, 128.6, 129.1, 129.5, 130.5, 131.7, 132.4, 133.3, 133.7, 136.3, 133.7, 136.6, 140.2, 154.2, 163.7, 164.6 ppm; anal. C 58.72, H 4.03, N 10.98% calcd. for C_31_H_26_ClN_5_O_4_S_2_, C 58.90, H 4.15, N 11.08%.

*4-Benzylthio-2-chloro-5-{N-[5-(3-methoxyphenyl)-1,2,4-triazin-3-yl]sulfamoyl}-N-(4-methylphenyl)benzamide* (**54**). Starting from **25** (0.504 g) and 3-methoxyphenylglyoxal hydrate (0.182 g) after stirring for 33 h, the title compound **54** (0.480 g, 76%) was obtained without further purification mp 237–239 °C; IR (KBr) ν_max_ 3328 (NH), 3029, 2918 (C–H), 1678 (C=O), 1554, 1519 (C=C, C=N), 1314, 1162 (SO_2_) cm^−1^; ^1^H-NMR (200 MHz, DMSO-*d*_6_) δ 2.31 (s, 3H, CH_3_), 3.77 (s, 3H, OCH_3_), 4.42 (s, 2H, SCH_2_), 7.19–7.21 (m, 6H, arom), 7.36–7.46 (m, 4H, arom), 7.58–7.69 (m, 4H, H-3, arom), 8.24 (s, 1H, H-6), 9.28 (s, 1H, triazine), 10.50 (s, 1H, NHCO) ppm; ^13^C-NMR (50 MHz, DMSO-*d*_6_) δ 20.8, 35.9, 55.7, 114.2, 119.9, 120.1, 121.3, 127.5, 127.9, 128.6, 129.2, 129.4, 130.5, 130.9, 132.5, 133.2, 133.7, 136.2, 136.5, 140.3, 154.2, 159.9, 163.5 ppm; anal. C 59.06, H 4.25, N 11.12% calcd. for C_31_H_26_ClN_5_O_4_S_2_, C 58.90, H 4.15, N 11.08%.

*4-Benzylthio-2-chloro-5-{N-[5-(3,4-dimethoxyphenyl)-1,2,4-triazin-3-yl]sulfamoyl}-N-(4-methylphenyl)-benzamide* (**55**). Starting from **25** (0.504 g) and 3,4-dimethoxyphenylglyoxal hydrate (0.212 g) after stirring for 33 h, the crude **55** (0.570 g) was obtained. The crude **55** was purified by heating under reflux with EtOH and the title compound **55** (0.480 g, 72%) was isolated mp 250–252 °C; IR (KBr) ν_max_ 3329, 3093 (NH), 2921, 2852 (C–H), 1640 (C=O), 154, 1518 (C=N, C=C), 1318, 1132 (SO_2_) cm^−1^; ^1^H-NMR (200 MHz, DMSO-*d*_6_) δ 2.30 (s, 3H, CH_3_), 3.74 (s, 3H, OCH_3_), 3.79 (s, 3H, OCH_3_), 4.41 (s, 2H, SCH_2_), 7.00–7.04 (m, 1H, arom), 7.15–7.25 (m, 5H, arom), 7.37–7.42 (m, 2H, arom), 7.56–7.68 (m, 5H, arom), 8.25 (s, 1H, H-6, arom), 9.19 (s, 1H, triazine), 10.49 (s, 1H, NHCO) ppm; anal. C 56.03, H 4.19, N 10.36% calcd. for C_32_H_28_ClN_5_O_5_S_2_, C 56.23, H 4.26, N 10.58%.

*4-Benzylthio-2-chloro-N-(4-methylphenyl)-5-{N-[5-(3,4,5-trimethoxyphenyl)-1,2,4-triazin-3-yl]sulfamoyl}-benzamide* (**56**). Starting from **24** (0.504 g) and 3,4,5-trimethoxyphenylglyoxal hydrate (0.242 g) after stirring for 33 h, the crude **56** (0.516 g) was obtained. The crude **56** was purified by heating under reflux with EtOH and the title compound **56** (0.464 g, 67%) was isolated mp 259–261 °C; IR (KBr) ν_max_ 3435, 3308 (NH), 3033, 2924 (C–H), 1640 (C=O), 1558, 1522 (C=C, C=N), 1342, 1129 (SO_2_) cm^−1^; ^1^H-NMR (200 MHz, DMSO-*d*_6_) δ 2.29 (s, 3H, CH_3_), 3.71 (s, 3H, OCH_3_), 3.80 (s, 6H, 2×OCH_3_), 4.43 (s, 2H, SCH_2_), 7.14–7.26 (m, 5H, arom), 7.40–7.63 (m, 7H, H-3, arom), 8.20 (s, 1H, H-6, arom), 9.32 (s, 1H, triazine), 10.45 (s, 1H, NHCO), 14.20 (brs, 1H, SO_2_NH) ppm; ^13^C-NMR (50 MHz, DMSO-*d*_6_) δ 20.8, 35.9, 56.2, 60.5, 106.6, 119.8, 127.4, 127.6, 127.7, 128.7, 129.3, 129.4, 130.0, 132.3, 133.2, 133.9, 136.2, 136.4, 140.4, 143.5, 153.5, 154.5, 163.4 ppm; anal. C 56.89, H 4.02, N 9.95% calcd. for C_33_H_30_ClN_5_O_6_S_2_, C 57.26, H 4.37, N 10.12%.

*4-Benzylthio-2-chloro-N-(4-methoxyphenyl)-5-{N-[5-(4-methoxyphenyl)-1,2,4-triazin-3-yl]sulfamoyl}benzamide* (**57**). Starting from **26** (0.520 g) and 4-methoxyphenylglyoxal hydrate (0.182 g) after stirring for 33 h, the title compound **57** (0.571 g, 88%) was obtained without further purification mp 270–272 °C; IR (KBr) ν_max_ 3331 (NH), 3092, 2932, 2835 (CH_2_, CH_3_), 1676 (C=O), 1608, 1543 (C=N, C=C), 1313, 1162 (SO_2_) cm^−1^; ^1^H-NMR (200 MHz, DMSO-*d*_6_) δ 3.77 (s, 3H, OCH_3_), 3.79 (s, 3H, OCH_3_), 4.39 (s, 2H, SCH_2_), 6.96 (d, *J* = 9.0 Hz, 2H, arom), 7.05 (d, *J* = 9.00 Hz, 2H, arom), 7.17–7.20 (m, 3H, arom), 7.33–7.35 (m, 2H, arom), 7.63–7.68 (m, 3H, arom), 7.95 (d, *J* = 8.8 Hz, 2H, arom), 8.26 (s, 1H, H-6, arom), 9.10 (s, 1H, triazine), 10.49 (s, 1H, NHCO) ppm; ^13^C-NMR (50 MHz, DMSO-*d*_6_) δ 35.8, 55.5, 55.9, 114.3, 115.3, 121.4, 124.5, 127.5, 127.9, 128.6, 129.1, 130.5, 131.7, 132.2, 132.5, 133.8, 136.3, 140.1, 156.0, 163.5, 164.6 ppm; anal. C 57.50, H 4.09, N 10.85% calcd. for C_31_H_26_ClN_5_O_5_S_2_, C 57.44, H 4.04, N 10.81%.

*4-Benzylthio-2-chloro-N-(4-methoxyphenyl)-5-{N-[5-(3-methoxyphenyl)-1,2,4-triazin-3-yl]sulfamoyl}benzamide* (**58**). Starting from **26** (0.520 g) and 3-methoxyphenylglyoxal hydrate (0.182 g) after stirring for 33 h, the crude **58** (0.550 g) was obtained. The crude **58** was purified by heating under reflux with EtOH and the title compound **58** (0.470 g, 72%) was isolated mp 242–244 °C; IR (KBr) ν_max_ 3333 (NH), 3083, 2922 (CH_2_, CH_3_), 2360 (OCH_3_), 1679 (C=O), 1600, 1552, 1510 (C=N, C=C), 1307, 1162 (SO_2_) cm^−1^; ^1^H-NMR (200 MHz, DMSO-*d*_6_) δ 3.77 (s, 6H, 2×OCH_3_), 4.41 (m, 2H, SCH_2_), 6.96 (d, *J* = 8.8 Hz, 2H, arom), 7.19–7.21 (m, 4H, arom), 7.35–7.45 (m, 4H, arom), 7.59–7.68 (m, 4H, arom), 8.23 (s, 1H, arom), 9.27 (s, 1H, triazine), 10.43 (s, 1H, NHCO) ppm; ^13^C-NMR (50 MHz, DMSO-*d*_6_) δ 35.9, 55.5, 55.7, 114.2, 120.0, 121.3, 121.4, 127.5, 128.0, 128.6, 129.2, 130.5, 130.9, 132.2, 132.5, 133.7, 136.2, 140.2, 156.0, 160.0, 163.2 ppm; anal. C 57.32, H 3.88, N 10.70% calcd. for C_31_H_26_ClN_5_O_5_S_2_, C 57.44, H 4.04, N 10.81%.

*4-Benzylthio-2-chloro-5-{N-[5-(3,4-dimethoxyphenyl)-1,2,4-triazin-3-yl]sulfamoyl}-N-(4-methoxyphenyl)-benzamide* (**59**). Starting from **26** (0.520 g) and 3,4-dimethoxyphenylglyoxal hydrate (0.212 g) after stirring for 33 h, the title compound **59** (0.625 g, 92%) was obtained without further purification mp 269–271 °C; IR (KBr) ν_max_ 3393, 3262 (NH), 2924, 2851 (C–H), 1679 (C=O), 1543, 1514 (C=C, C=N), 1312, 1126 (SO_2_) cm^−1^; ^1^H-NMR (200 MHz, DMSO-*d*_6_) δ 3.76-3.78 (m, 9H, 3×OCH_3_), 4.41 (s, 2H, SCH_2_), 6.92–7.06 (m, 3H, arom), 7.22–7.25 (m, 3H, H-3, arom), 7.38–7.49 (m, 2H, arom), 7.59–7.67 (m, 5H, arom), 8.26 (s, 1H, H-6, arom), 9.19 (s, 1H, triazine), 10.43 (s, 1H, NHCO) ppm; ^13^C-NMR (50 MHz. DMSO-*d*_6_) δ 35.9, 55.5, 55.9, 111.5, 112.3, 114.2, 121.3, 124.5, 127.6, 127.7, 128.7, 129.2, 132.2, 132.3, 133.8, 136.1, 136.2, 140.4, 149.3, 154.5, 156.0, 163.3 ppm; anal. C 56.52, H 4.05, N 10.22% calcd. for C_32_H_28_ClN_5_O_6_S_2_, C 56.68, H 4.17, N 10.33%.

*4-Benzylthio-2-chloro-N-(4-methoxyphenyl)-5-{N-[5-(3,4,5-trimethoxyphenyl)-1,2,4-triazin-3-yl]sulfamoyl}-benzamide* (**60**). Starting from **26** (0.520 g) and 3,4,5-trimethoxyphenylglyoxal hydrate (0.242 g) after stirring for 33 h, the crude **60** (0.610 g) was obtained. The crude **60** was purified by heating under reflux with EtOH and the title compound **60** (0.519 g, 73%) was isolated mp 263–265 °C; IR (KBr) ν_max_ 3309 (NH), 2924, 2834 (C–H), 1639 (C=O), 1558, 1513 (C=N, C=C), 1334, 1132 (SO_2_) cm^−1^; ^1^H-NMR (200 MHz, DMSO-*d*_6_) δ 3.71-3.79 (m, 12H, 4×OCH_3_), 4.42 (s, 2H, SCH_2_), 6.92 (d, *J* = 8.9 Hz, 2H, arom), 7.23–7.26 (m, 3H, arom), 7.40–7.46 (m, 4H, arom), 7.53–7.62 (m, 3H, arom), 8.19 (s, 1H, H-6, arom), 9.32 (s, 1H, triazine), 10.38 (s, 1H, NHCO) ppm; anal. C 55.84, H 3.88, N 9.55% calcd. for C_33_H_30_ClN_5_O_7_S_2_, C 55.97, H 4.27, N 9.89%.

### 3.3. X-ray Structure Determination

Crystals of **59** were grown from DMF solution. All specimens obtained were in form of very thin needles, having poor diffraction power. The copper radiation (almost no diffraction was observed with the Mo lamp) and long exposure times (4 min) were applied. The obtained signal to noise ratio was still smaller than usually, so final *R*_int_ and *R*_1_ indices are above the regular standards for small molecules. Nevertheless, structure solution is with no doubts generally correct. Diffraction intensity data were collected on an IPDS 2T dual-beam diffractometer (STOE & Cie GmbH, Darmstadt, Germany) at 120.(2) K with Cu-Kα radiation of a microfocus X-ray source (50 kV, 0.6 mA, λ = 154.186 pm, GeniX 3D Cu High Flux, Xenocs, Sassenage, France), The crystal was thermostated in nitrogen stream at 120 K using CryoStream-800 device (Oxford CryoSystem, Oxford, UK) during the entire experiment. Data collection and data reduction were controlled by X-Area 1.75 program [43]. An absorption correction was performed on the integrated reflections by a combination of frame scaling, reflection scaling and a spherical absorption correction. Outliers have been rejected according to Blessing’s method [44].

The structure was solved using direct methods with SHELXS-13 program and refined by SHELXL-2013 [45] program run under control of WinGx [46]. All C-H type hydrogen atoms were attached at their geometrically expected positions and refined as riding on heavier atoms with the usual constraints. The N-H hydrogen atoms were found in the differential Fourier electron density map and were refined without constraints.

Crystallographic data for the analysis have been deposited with the Cambridge Crystallographic data Centre, CCDC reference number is 1471508. Copies of this information may be obtained free of charge from CCDC, 12 Union Road, Cambridge, CB21EZ, UK (Fax: +44-1223-336033; E-mail: deposit@ccdc.cam.ac.uk or www: http://www.ccdc.cam.ac.uk).

### 3.4. Cell Culture and Cell Viability Assay

All chemicals, if not stated otherwise, were obtained from Sigma-Aldrich (St. Louis, MO, USA). The MCF-7 cell line was purchased from Cell Lines Services (Eppelheim, Germany), the HeLa and HCT-116 cell lines were obtained from the Department of Microbiology, Tumor and Cell Biology, Karolinska Institute (Stockholm, Sweden). Cells were cultured in Dulbecco’s modified Eagle’s medium (DMEM) supplemented with 10% fetal bovine serum, 2 mM glutamine, 100 units/mL penicillin, and 100 μg/mL streptomycin. Cultures were maintained in a humidified atmosphere with 5% CO_2_ at 37 °C in an incubator (HeraCell, Heraeus, Langenselbold, Germany).

Cell viability was determined using the MTT (3-(4,5-dimethylthiazol-2-yl)-2,5-diphenyl-tetrazoliumbromide) assay. Stock solutions of the studied compounds were prepared in 100% DMSO. Working solutions were prepared by diluting the stock solutions with DMEM medium, the final concentration of DMSO did not exceed 0.5% in the treated samples. Cells were seeded in 96-well plates at a density of 5 × 10^3^ cells/well and treated for 72 h with the examined compounds in the concentration range 1–100 μM (1, 10, 25, 50 and 100 μM). Following treatment, MTT (0.5 mg/mL) was added to the medium and cells were further incubated for 2 h at 37 °C. Cells were lysed with DMSO and the absorbance of the formazan solution was measured at 550 nm with a plate reader (1420 multilabel counter, Victor, Jügesheim, Germany). The optical density of the formazan solution was measured at 550 nm with a plate reader (Victor 1420 multilabel counter). The experiment was performed in triplicate. Values are expressed as the mean ± SD of at least three independent experiments.

#### 3.4.1. Cell Morphology

The HCT-116, HeLa and MCF-7cells were seeded on 24-well plates (5 × 10^4^/per well) in 300 μL of medium and incubated with **37** (HCT-116, HeLa) or **46** (MCF-7) for 24 h. Cells incubated with increasing concentrations of compounds (25–100 μM) were observed after 24 h using light microscope Axiovert 200 (Zeiss, Oberkochen, Germany).

#### 3.4.2. Cell Cycle Analysis

Cells were coincubated with compounds and a reference drug (cisplatin) for 24 h, respectively. Following treatment, cells were fixed in cold 70% ethanol for 30 min. Fixed cells were stained for 15 min in buffer containing 500 μg/mL RNAse A (EURx, Gdansk, Poland) and 2.5 μg/mL of DAPI (Sigma-Aldrich, Munich, Germany). The fluorescence of DAPI was measured at 440 ± 40 nm with a flow cytometer (LSR II, BD Biosciences, Mountain View, CA, USA). Each experiment was performed at least in triplicate. Data were analyzed off-line using the BD FACSDiva (BD Biosciences).

#### 3.4.3. Mitochondrial Membrane Potential (Δψ_m_) Analysis

The HCT-116, HeLa nad MCF-7 cells were coincubated with trazines and a reference drug (cisplatin) for 24 h, respectively. Half an hour before the end of 24 h-treatment 2.5 μg/mL of JC-1 (5,5′,6,6′-tetrachloro-1,1′,3,3′-tetraethylbenzimidazolylcarbocyanine iodide) was added to the cell medium. As a negative control 100 μM CCCP (carbonyl cyanide 3-chlorophenylhydrazone) was used 15 min before JC-1 treatment. Following treatment cells were twice washed with PBS and analyzed with flow cytometer (LSR II, BD Biosciences).

Green and red fluorescence were observed using FL-1 (530 ± 30 nm) and FL-2 (585 ± 42 nm) channels, respectively. Percentage of cell population in red or green fluorescence gate indicated the level of Δψ_m_. Decreasing red fluorescence cell population indicated lower Δψ_m_.

#### 3.4.4. Translocation of Phosphatidylserine to Outer Leaflet of Cell Membrane

Analysis was performed using FITC-conjugated annexin-V (Annexin V-FITC Apoptosis Kit I, BD Biosciences) according to the manufacturer’s instructions (BD Biosciences) as described previously [47]. HCT-116, HeLa and MCF-7 cells were coincubated for 24 h with traizines **37** and **46**, respectively. Following treatment cells were washed in PBS, centrifuged and then resuspended in binding buffer. Afterwards, the cells were incubated for 15 min at 37 °C with FITC-conjugated annexin-V and propidium iodide. The samples were then analyzed using a LSR II flow cytometer (BD Biosciences) using 525 ± 20 nm (Annexin V-FITC) and 570 ± 26 nm (PI). The subpopulations were identified according to their fluorescence: PI-low/FITC-low (live cells), PI-high/FITC-low (necrotic cells), PI-low/FITC-high (early apoptotic cells), PI-high/FITC-high (late apoptotic cells).

#### 3.4.5. Caspase Activity Determination

Caspase activity was determined with the FLICA Apoptosis Detection Kit (Immunochemistry Technologies, Bloomington, MN, USA) according to the manufacturer’s instructions. Cells were treated with the examined compounds for the indicated periods of time after which cells were collected and suspended in a buffer containing the caspase inhibitor—a carboxyfluorescein-labeled fluoromethyl ketone peptide. Cells were subsequently incubated for 1 h 37 °C under 5% CO_2_ cells and next were washed with washing buffer. The fluorescence intensity of fluorescein was determined with flow cytometry (BD FACSCalibur) and caspase activity was determined as the amount of fluorescence emitted from caspase inhibitors bound to the caspases.

#### 3.4.6. Statistical Analysis

Statistical differences between control and triazine coincubated cells were determined using the t-student test, after checking the normal distribution of data. The analyses were performed using 6 to 15 replicates run in at least three independent experiments.

### 3.5. Metabolic Stability

Stock solutions of studied compounds were prepared at concentration of 10 mM in DMSO. Working solutions were prepared by dilution of stock with reaction buffer or acetonitrile, final concentration of organic solvent did not exceed 1%. Incubation mixture contained 10 μM of a studied derivative, 1 mM of NADPH (Sigma-Aldrich) and 0.5 mg/mL of human liver microsomes (HLM, Sigma-Aldrich) in potassium phosphate buffer (0.1 M, pH 7.4). Incubation was carried out in thermostat at 37 °C and started by addition of studied compound. 50 μL samples were taken after 5, 15, 30, 45 and 60 min. Enzymatic reaction was terminated by the addition of the equal volume of ice-cold acetonitrile containing 10 μM of alprazolam (Sigma-Aldrich) serving as internal standard (IS). Control incubations were performed without NADPH to assess chemical instability. After collection, samples were immediately centrifuged (10 min, 10,000 rpm) and resulted supernatant was directly analyzed or kept in −80 °C until LC-MS analysis. Natural logarithm of a compound over IS peak area ratio was plotted *versus* incubation time. Metabolic half-time (t_1/2_) was calculated from the slope of the linear regression, as reported in [48].

LC-MS analysis was performed on an Agilent 1260 system coupled to SingleQuad 6120 mass spectrometer (Agilent Technologies, Santa Clara, CA, USA). Ascentis Express ES-CN (2.1 mm × 100 mm, 2.7 μm, Supelco Analytical, Bellefonte, PA, USA) was used in reversed-phase mode with gradient elution starting with 50% of phase A (10 mM ammonium formate in water) and 50% of phase B (10 mM ammonium formate in acetonitrile-water mixture, 95:5 *v*/*v*). The amount of phase B was linearly increased to 100% in 5 min. Total analysis time was 10 min at 25 °C, flow rate was 0.4 mL/min and the injection volume was 20 μL. The mass spectrometer was equipped with electrospray ion source and operated in positive ionization. Mass analyzer was set individually to each compound to detect [M + H]^+^ pseudomolecular ions. MSD parameters of the ESI source were as follows: nebulizer pressure 50 psig (N_2_), drying gas 13 mL/min (N_2_), drying gas temperature 300 °C, capillary voltage 3.5 kV, fragmentor voltage 100 V.

## 4. Conclusions

We have synthesized a series of novel 4-chloro-2-(R^2^-methylthio)-5-R^1^-*N*-(5-aryl-1,2,4-triazin-3-yl)benzenesulfonamide derivatives using aminoguanidines and appropriate phenylglyoxal hydrates in glacial acetic acid. The molecular structures of novel compounds were confirmed by NMR, IR, and also by single crystal X-ray structure analysis for representative compound **59**. All *N*-(5-aryl-1,2,4-triazin-3-yl)benzenesulfonamides were tested for their *in vitro* cytotoxic activity against the HCT-116, HeLa and MCF-7 cell lines. The most active compounds **37** and **38** belonged to the 1-naphthyl series (R^2^ = 1-naphthyl) and showed outstanding average cytotoxic activity against all tested cell lines with special regard to the HCT-116 (IC_50_: 36–38 μM) and HeLa (IC_50_: 34–42 μM) cell lines. The MCF-7 cell line occurred less sensitive to 1,2,4-triazine derivatives, the strongest cytotoxic potency showed **46**. The ability to induce apoptosis in cancer cells was studied through biochemical markers, such as: DNA fragmentation, loss of mitochondrial membrane potential, phosphatidylserine translocation and caspase activation. Apoptotic-like changes in morphology of HCT-116, HeLa, MCF-7 cell lines, significant accumulation of the HCT-116, HeLa and MCF-7 population in the sub-G1 phase and DNA fragmentation, loss of Δψ_m_, appearance of increased population of early and late apoptotic cells, induction of caspase activity indicated apoptosis of the cells in presence of **37** and **46**. *In vitro* tests for metabolic stability in the presence of human liver microsomes showed that both R^2^ and Ar substituents (4-CF_3_-C_6_H_4_) substantially increased the metabolic stability of 4-chloro-2-(R^2^-methylthio)-5-R^1^-*N*-(5-aryl-1,2,4-triazin-3-yl)benzenesulfonamides. The results pointed to **37** as a hit compound with a good biological response against HCT-116 (IC_50_ = 36 μM) and HeLa (IC_50_ = 34 μM) cell lines with apoptosis-inducing activity, and satisfactory metabolic stability.

## Figures and Tables

**Table 1:** Crystal data and structure refinement of **59**.

Empirical Formula	C_38_H_42_ClN_7_O_8_S_2_
Formula weight	824.35
Temperature	120(2) K
Wavelength	1.54186 Å
Crystal system	Triclinic
Space group	$P\bar{1}$
Unit cell dimensions	*a* = 7.8336(6) Å, α = 84.84(3)°
	*b* = 22.4284(8) Å, β = 86.95(4)°
	*c* = 22.2739(9) Å, γ = 85.13(5)°
Volume	3879.4(5) Å^3^
*Z*	4
Density (calculated)	1.411 Mg/m^3^
Absorption coefficient	2.399 mm^−1^
*F*(000)	1728
Crystal size	0.03 × 0.03 × 0.25 mm^3^
Theta range for data collection	1.984 to 64.981°
Index ranges	−8 ≤ *h* ≤ 8, −23 ≤ *k* ≤ 26, −25 ≤ *l* ≤ 23
Reflections collected	19645
Independent reflections	11676 [R(int) = 0.1757]
Completeness to *θ* = 67.686°	83.1%
Refinement method	Full-matrix least-squares on F^2^
Data/restraints/parameters	11,676/0/1014
Goodness-of-fit on F^2^	1.253
Final *R* indices [I > 2σ(I)]	R_1_ = 0.1476, wR^2^ = 0.3359
*R* indices (all data)	R_1_ = 0.2313, wR^2^ = 0.4260
Extinction coefficient	0.0114(11)

**Table 2:** Cytotoxicity of compounds **27**–**60** toward human cancer cell lines ^a^.

Compound	IC_50_ (μM)		
	HCT-116	HeLa	MCF-7
**27**	75 ± 1	84 ± 1	80 ± 2
**28**	200 ± 4	100 ± 6	120 ± 5
**29**	720 ± 36	190 ± 7	110 ± 2
**30**	85 ± 2	90 ± 4	67 ± 1
**31**	140 ± 7	150 ± 6	77 ± 2
**32**	88 ± 3	87 ± 4	87 ± 4
**33**	67 ± 1	76 ± 3	88 ± 1
**34**	51 ± 1	73 ± 1	68 ± 3
**35**	68 ± 3	69 ± 1	78 ± 2
**36**	49 ± 2	55 ± 2	96 ± 4
**37**	36 ± 1	34 ± 2	70 ± 3
**38**	38 ± 2	42 ± 1	69 ± 1
**39**	83 ± 2	93 ± 1	95 ± 3
**40**	77 ± 1	85 ± 2	98 ± 3
**41**	97 ± 2	85 ± 1	99 ± 1
**42**	77 ± 2	82 ± 1	83 ± 2
**43**	92 ± 3	130 ± 10	87 ± 2
**44**	160 ± 6	160 ± 8	90 ± 5
**45**	97 ± 2	82 ± 1	90 ± 1
**46**	64 ± 1	80 ± 2	59 ± 2
**47**	75 ± 5	79 ± 1	78 ± 3
**48**	80 ± 3	80 ± 2	87 ± 3
**49**	110 ± 2	120 ± 6	81 ± 5
**50**	115 ± 3	130 ± 8	120 ± 4
**51**	190 ± 13	*	95 ± 5
**52**	70 ± 2	*	69 ± 1
**53**	76 ± 2	74 ± 2	88 ± 2
**54**	84 ± 5	82 ± 1	96 ± 5
**55**	150 ± 3	105 ± 4	150 ± 6
**56**	75 ± 2	80 ± 2	89 ± 3
**57**	73 ± 1	80 ± 1	90 ± 1
**58**	98 ± 2	88 ± 6	92 ± 6
**59**	140 ± 5	150 ± 2	100 ± 5
**60**	230 ± 18	230 ± 5	270 ± 13
**Cisplatin**	3.8 ± 0.2	2.2 ± 0.2	3 ± 0.1

^a^ Analysis was performed using the MTT assay after 72 h of incubation. Values are expressed as the mean ± SD of at least three independent experiments; * Viability of cell lines at 100 μM of tested compounds were approximately 100%.

**Table 3:** Results of metabolic stability study.

Compound	*In Vitro* Metabolic Half-Life t_1/2_ (min)
**30**	24.9
**31**	17.6
**34**	>60 (154)
**35**	42.4
**36**	13.9
**37**	32.8
**38**	<5
**46**	>60 (78.1)
**47**	17.5
**52**	26.5

## Supplementary Materials

Supplementary materials can be accessed at: http://www.mdpi.com/1420-3049/21/6/808/s1.
