# Supplementary material for: Novel 5-Substituted 2-(Aylmethylthio)-4-chloro-N-(5-aryl-1,2,4-triazin-3-yl)benzenesulfonamides: Synthesis, Molecular Structure, Anticancer Activity, Apoptosis-Inducing Activity and Metabolic Stability
Source: Molecules. 2016 Jun 22;21(6):808. doi: 10.3390/molecules21060808 (PMC6273912; doi:10.3390/molecules21060808)
Supplement: Supplementary file 1 [file molecules-21-00808-s001.pdf]

Supplementary Materials: Novel 5-Substituted 2-(Aylmethylthio)-4-chloro-*N*-(5-aryl-1,2,4-triazin-3-yl)benzenesulfonamides: Synthesis, Molecular Structure, Anticancer Activity, Apoptosis-Inducing Activity and Metabolic Stability

Beata Żołnowska, Jarosław Sławiński, Aneta Pogorzelska, Krzysztof Szafrański, Anna Kawiak, Grzegorz Stasiłojć, Mariusz Belka, Szymon Ulenberg, Tomasz Bączek and Jarosław Chojnacki

No.	(ppm)	Hz
1	2.31	1156.6
2	4.44	2220.2
3	4.74	2367.6
4	6.93	3463.9
5	7.44	3720.7
6	7.46	3728.6
7	7.47	3735.9
8	7.51	3753.9
9	7.53	3763.2
10	7.54	3770.1
11	7.56	3777.9
12	7.57	3783.7
13	7.58	3788.1
14	7.59	3794.5
15	7.84	3918.0
16	7.86	3930.7
17	7.88	3939.0
18	7.94	3969.8
19	7.96	3977.6
20	8.22	4109.9
21	8.24	4118.2
22	8.38	4190.5

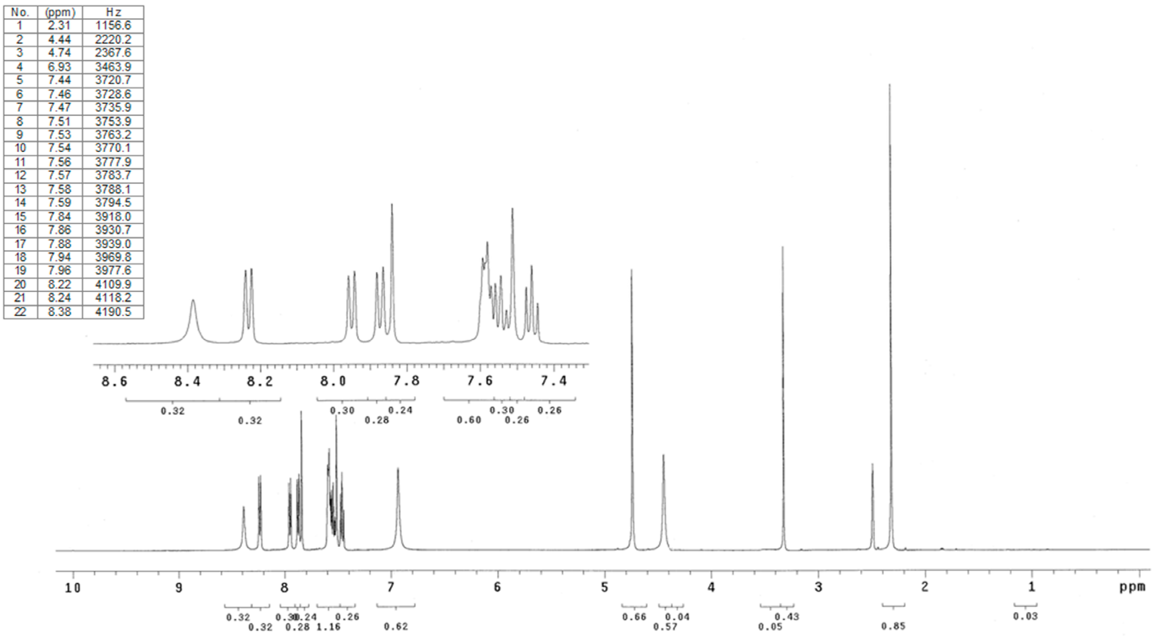

Figure S1. ^{1}H-NMR of compd **21** (500 MHz, DMSO-d_6).

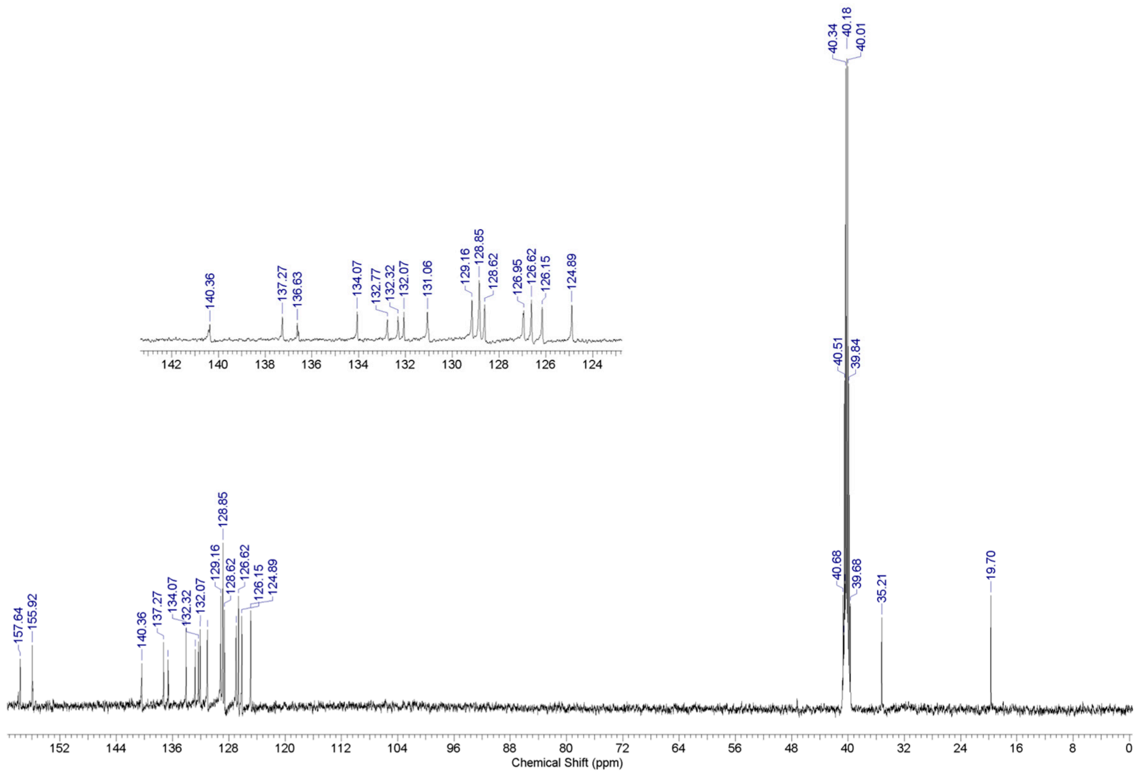

Figure S2. ^{13}C-NMR of compd **21** (125 MHz, DMSO-d_6).

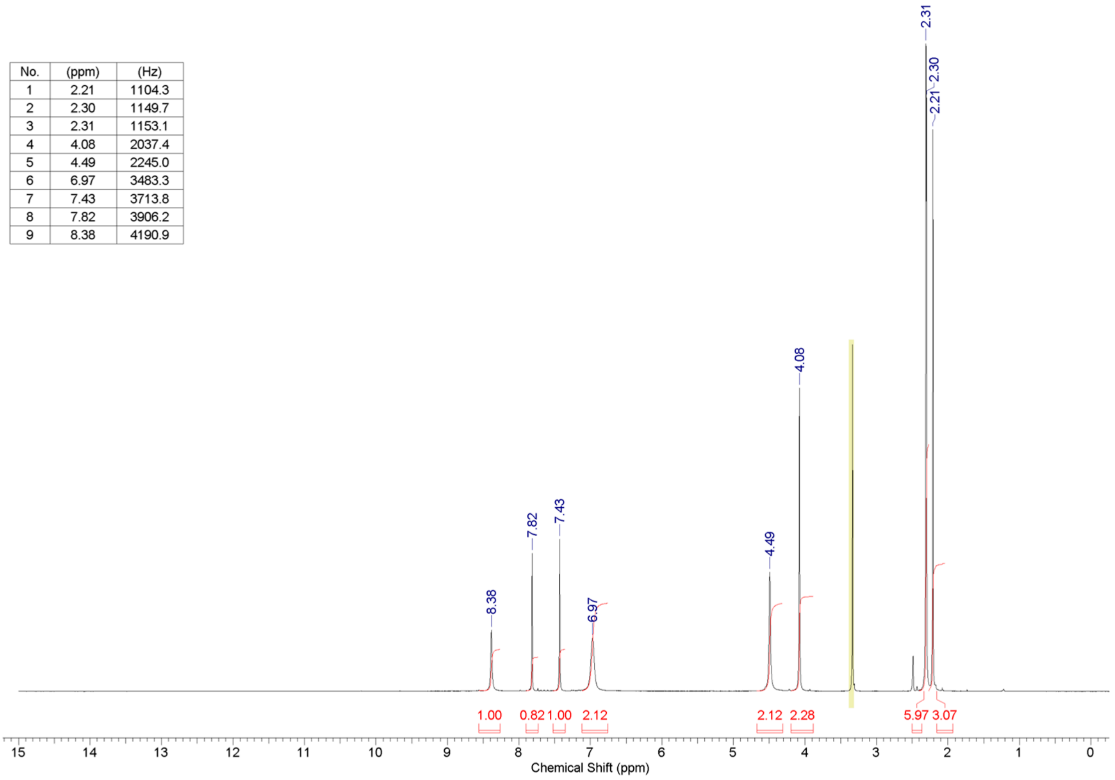

No.	(ppm)	(Hz)
1	2.21	1104.3
2	2.30	1149.7
3	2.31	1153.1
4	4.08	2037.4
5	4.49	2245.0
6	6.97	3483.3
7	7.43	3713.8
8	7.82	3906.2
9	8.38	4190.9

Figure S3. ^{1}H-NMR of compd **22** (500 MHz, DMSO-d_6).

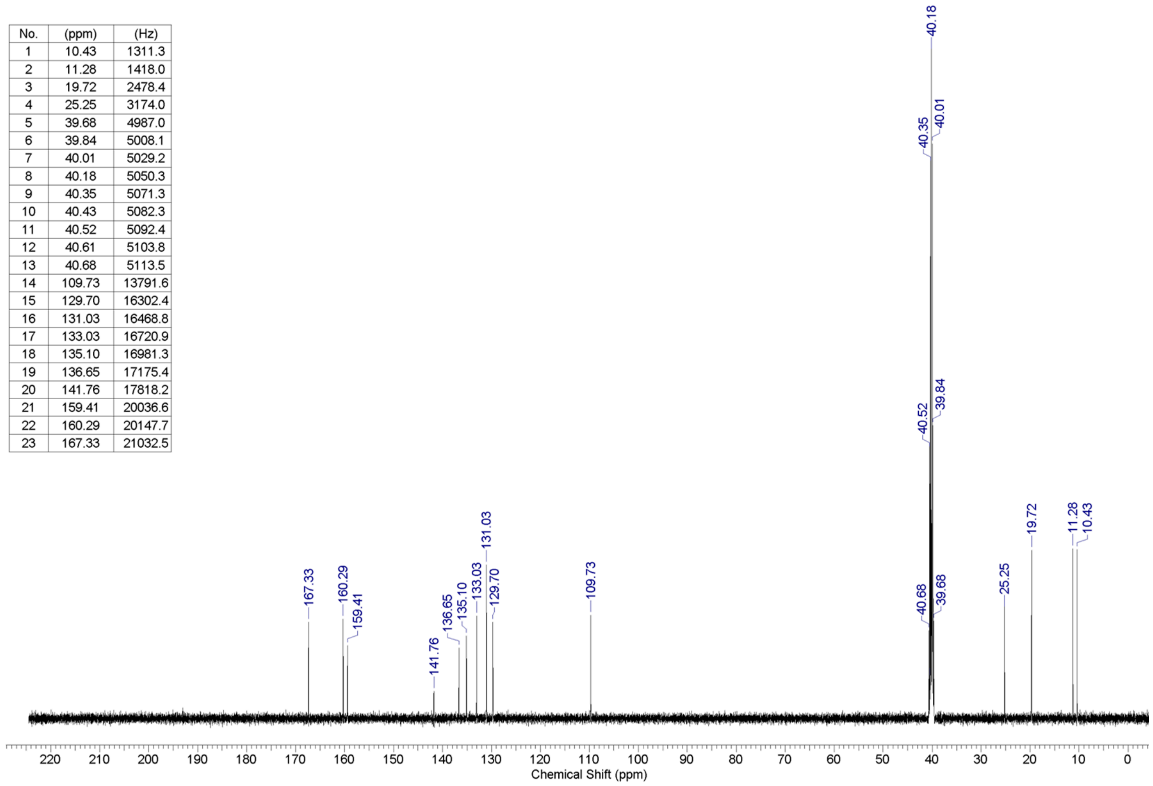

No.	(ppm)	(Hz)
1	10.43	1311.3
2	11.28	1418.0
3	19.72	2478.4
4	25.25	3174.0
5	39.68	4987.0
6	39.84	5008.1
7	40.01	5029.2
8	40.18	5050.3
9	40.35	5071.3
10	40.43	5082.3
11	40.52	5092.4
12	40.61	5103.8
13	40.68	5113.5
14	109.73	13791.6
15	129.70	16302.4
16	131.03	16468.8
17	133.03	16720.9
18	135.10	16981.3
19	136.65	17175.4
20	141.76	17818.2
21	159.41	20036.6
22	160.29	20147.7
23	167.33	21032.5

Figure S4. ^{13}C-NMR of compd **22** (125 MHz, DMSO-*d*$_6$).

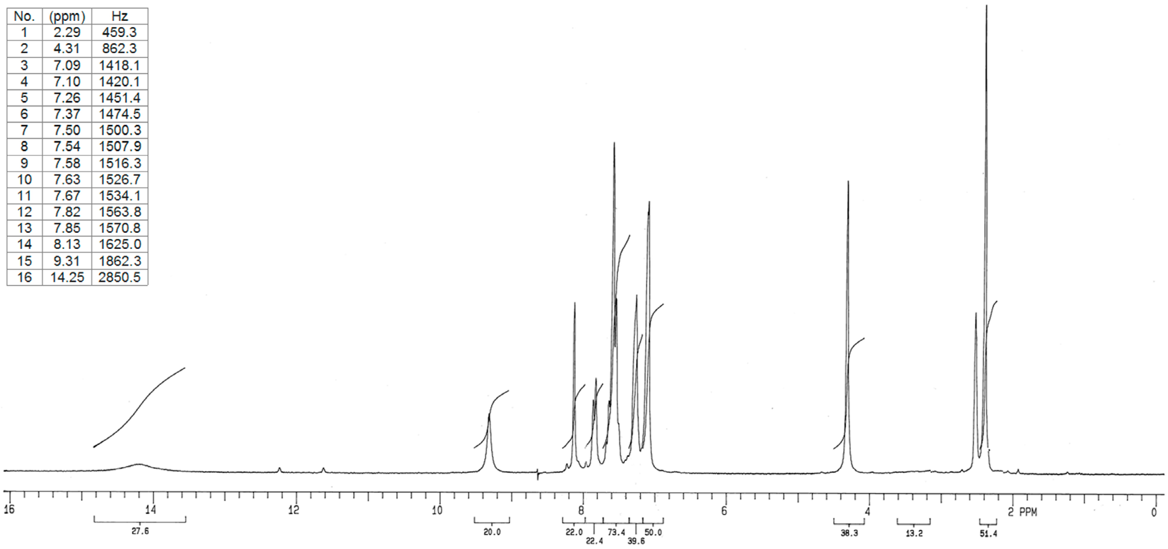

No.	(ppm)	Hz
1	2.29	459.3
2	4.31	862.3
3	7.09	1418.1
4	7.10	1420.1
5	7.26	1451.4
6	7.37	1474.5
7	7.50	1500.3
8	7.54	1507.9
9	7.58	1516.3
10	7.63	1526.7
11	7.67	1534.1
12	7.82	1563.8
13	7.85	1570.8
14	8.13	1625.0
15	9.31	1862.3
16	14.25	2850.5

Figure S5. ^{1}H-NMR of compd **27** (200 MHz, DMSO-*d*$_6$).

No.	(ppm)	Hz
1	19.1	960.5
2	36.2	1822.0
3	38.5	1938.4
4	38.9	1959.2
5	39.2	1972.0
6	39.4	1980.3
7	39.6	1993.1
8	39.8	2001.3
9	40.0	2013.4
10	40.2	2022.4
11	40.5	2035.7
12	40.6	2043.4
13	40.9	2056.4
14	41.0	2064.5
15	114.8	5774.6
16	115.3	5797.8
17	121.1	6090.1
18	125.1	6291.4
19	127.3	6401.0
20	128.3	6451.7
21	128.4	6459.1
22	129.1	6495.1
23	131.6	6616.5
24	131.7	6624.6
25	132.5	6661.1
26	132.7	6675.1
27	134.9	6784.2
28	135.1	6792.1
29	135.4	6807.6
30	136.5	6864.9
31	137.6	6919.5
32	160.1	8053.0
233	165.0	8298.3

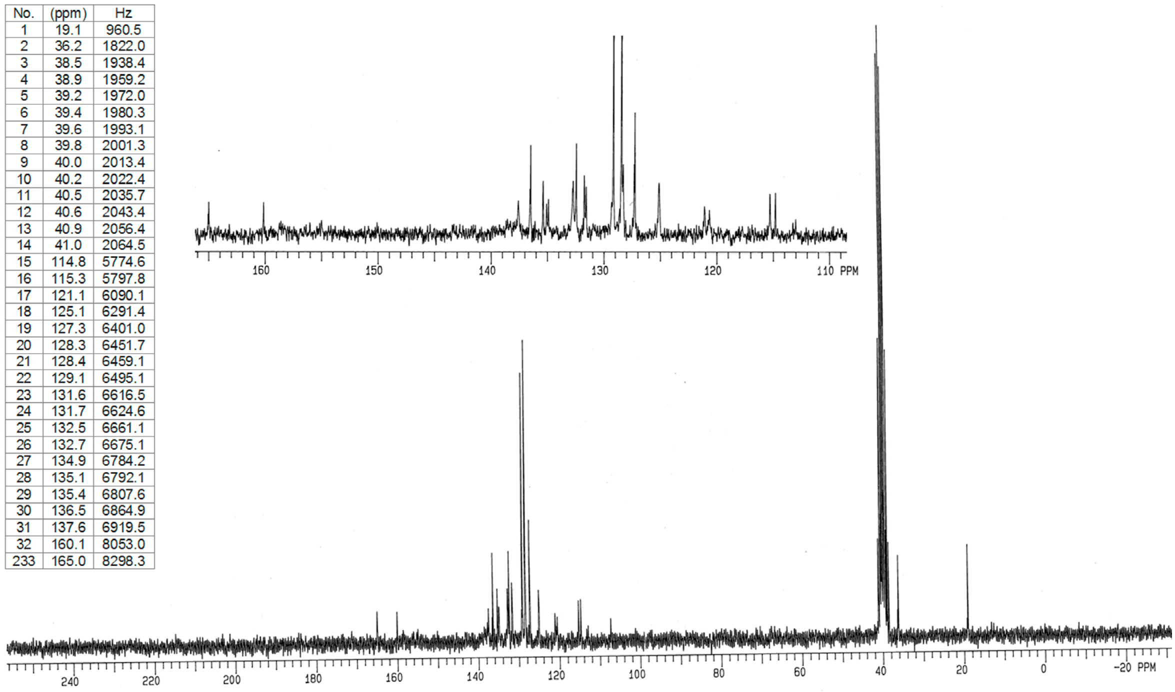

Figure S6. ^{13}C-NMR of compd **27** (50 MHz, DMSO-d_6).

No.	(ppm)	Hz
1	2.37	473.6
2	4.32	864.3
3	7.10	1420.6
4	7.11	1422.9
5	7.13	1426.1
6	7.28	1456.3
7	7.30	1459.6
8	7.57	1513.4
9	7.92	1583.4
10	7.96	1591.7
11	8.15	1630.6
12	8.17	1634.6
13	9.42	1883.0
14	14.10	2818.5

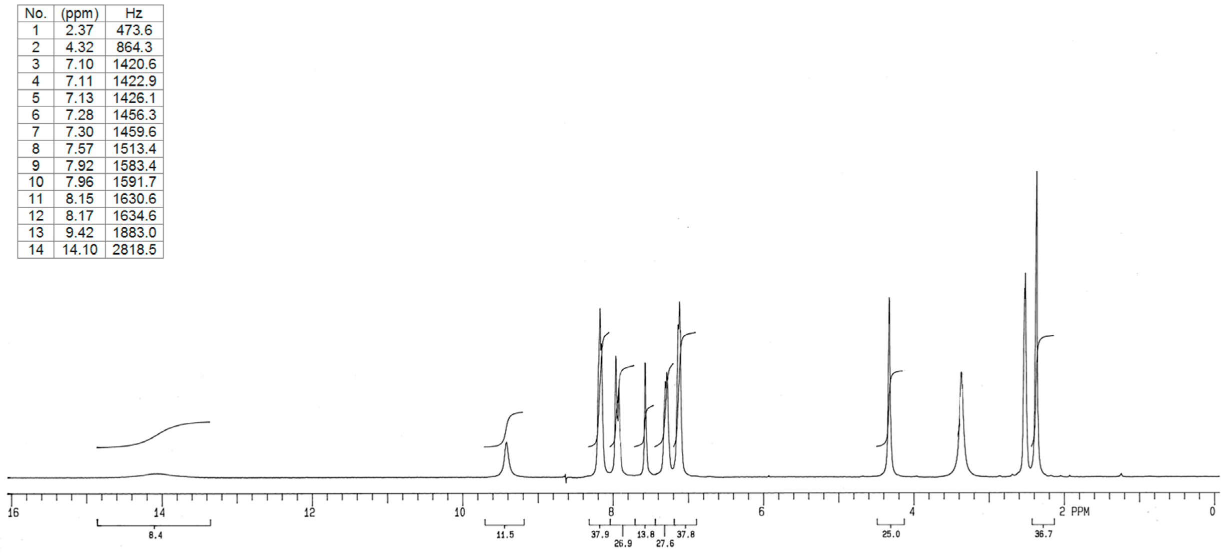

Figure S7. ^{1}H-NMR of compd **28** (200 MHz, DMSO-d_6).

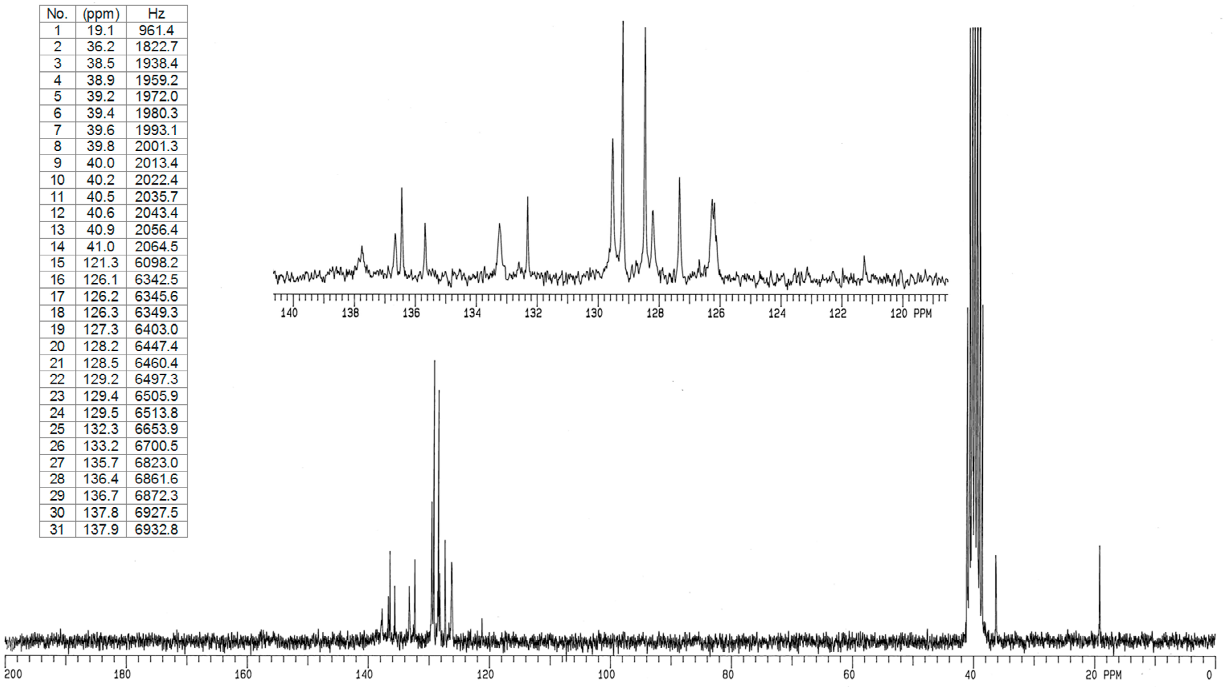

No.	(ppm)	Hz
1	19.1	961.4
2	36.2	1822.7
3	38.5	1938.4
4	38.9	1959.2
5	39.2	1972.0
6	39.4	1980.3
7	39.6	1993.1
8	39.8	2001.3
9	40.0	2013.4
10	40.2	2022.4
11	40.5	2035.7
12	40.6	2043.4
13	40.9	2056.4
14	41.0	2064.5
15	121.3	6098.2
16	126.1	6342.5
17	126.2	6345.6
18	126.3	6349.3
19	127.3	6403.0
20	128.2	6447.4
21	128.5	6460.4
22	129.2	6497.3
23	129.4	6505.9
24	129.5	6513.8
25	132.3	6653.9
26	133.2	6700.5
27	135.7	6823.0
28	136.4	6861.6
29	136.7	6872.3
30	137.8	6927.5
31	137.9	6932.8

Figure S8. ^{13}C-NMR of compd **28** (50 MHz, DMSO-d_6).

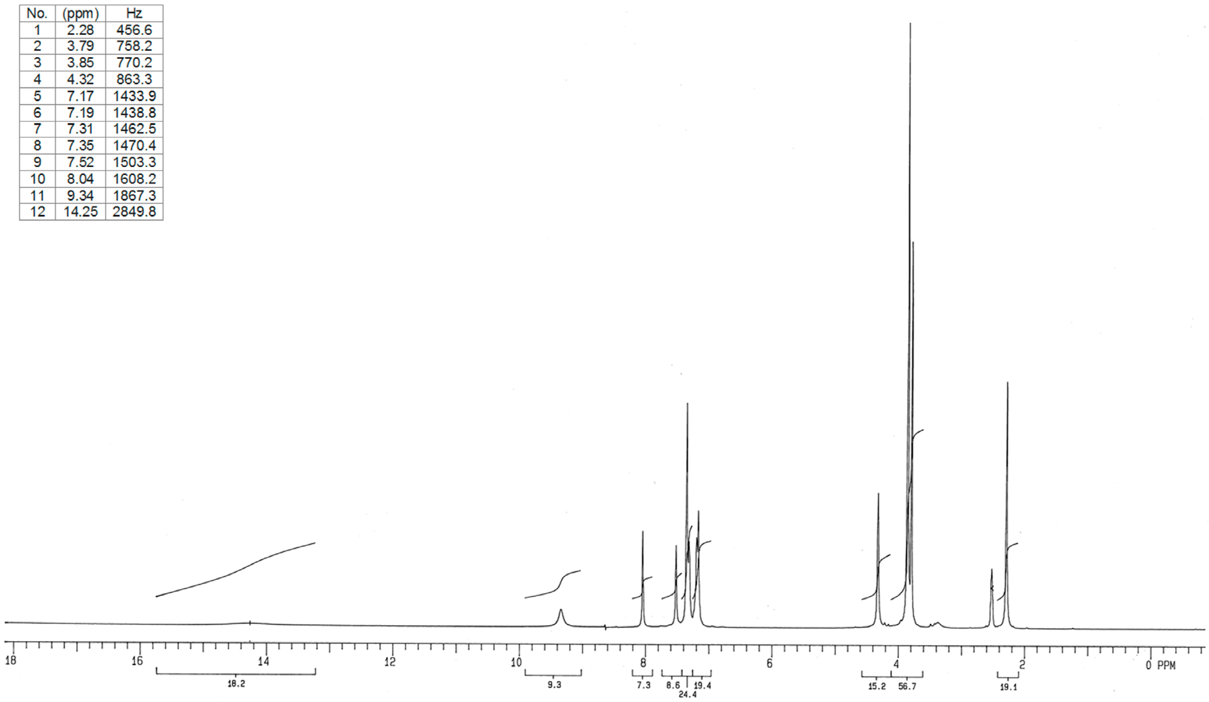

No.	(ppm)	Hz
1	2.28	456.6
2	3.79	758.2
3	3.85	770.2
4	4.32	863.3
5	7.17	1433.9
6	7.19	1438.8
7	7.31	1462.5
8	7.35	1470.4
9	7.52	1503.3
10	8.04	1608.2
11	9.34	1867.3
12	14.25	2849.8

Figure S9. ^{1}H-NMR of compd **32** (200 MHz, DMSO-d_6).

No.	(ppm)	Hz
1	19.1	960.8
2	36.3	1824.7
3	38.5	1938.4
4	38.9	1959.2
5	39.2	1972.0
6	39.4	1980.3
7	39.6	1993.1
8	39.8	2001.3
9	40.0	2013.4
10	40.2	2022.4
11	40.5	2035.7
12	40.6	2043.4
13	40.9	2056.4
14	41.0	2064.5
15	56.7	2853.6
16	60.7	3051.2
17	107.1	5384.4
18	127.4	6407.1
19	127.7	6419.7
20	127.9	6432.4
21	128.5	6464.9
22	129.2	6500.0
23	132.0	6637.3
24	132.1	6644.7
25	135.4	6808.8
26	136.4	6859.7
27	137.3	6905.0
28	146.2	7352.6
29	153.6	7723.7

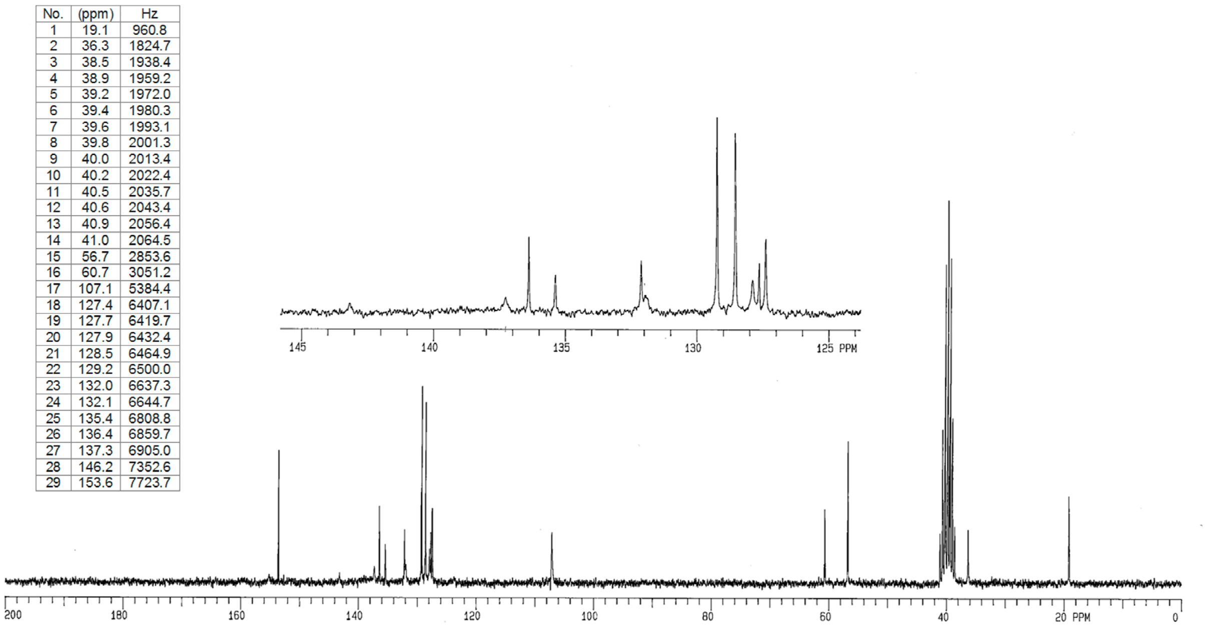

Figure S10. ^{13}C-NMR of compd **32** (50 MHz, DMSO-*d*$_6$).

No.	(ppm)	(Hz)
1	2.37	1186.3
2	3.86	1928.1
3	4.39	2194.2
4	7.03	3511.6
5	7.04	3520.4
6	7.43	3715.3
7	7.45	3722.6
8	7.49	3741.6
9	7.50	3749.5
10	7.52	3759.7
11	7.88	3940.9
12	7.90	3948.7
13	8.10	4048.8
14	9.04	4519.0
15	14.51	7251.6

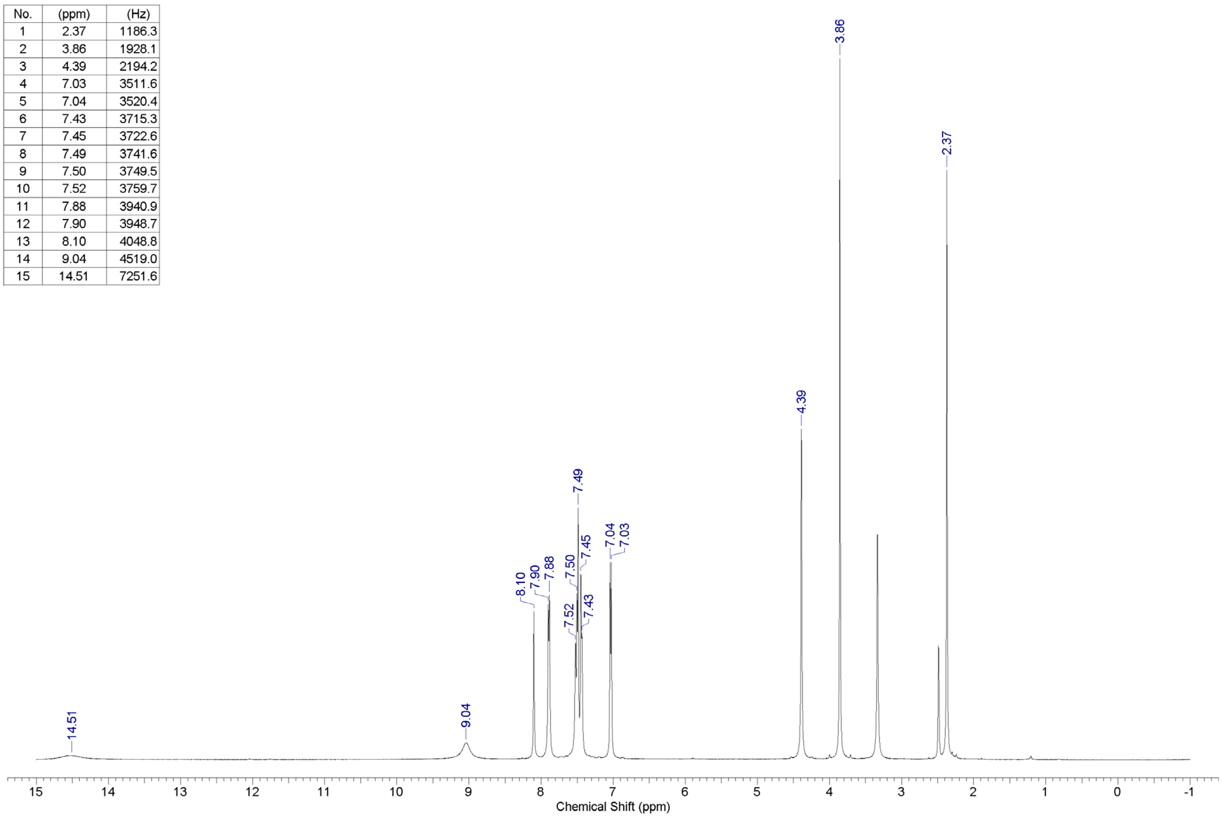

Figure S11. ^{1}H-NMR of compd **35** (500 MHz, DMSO-*d*$_6$).

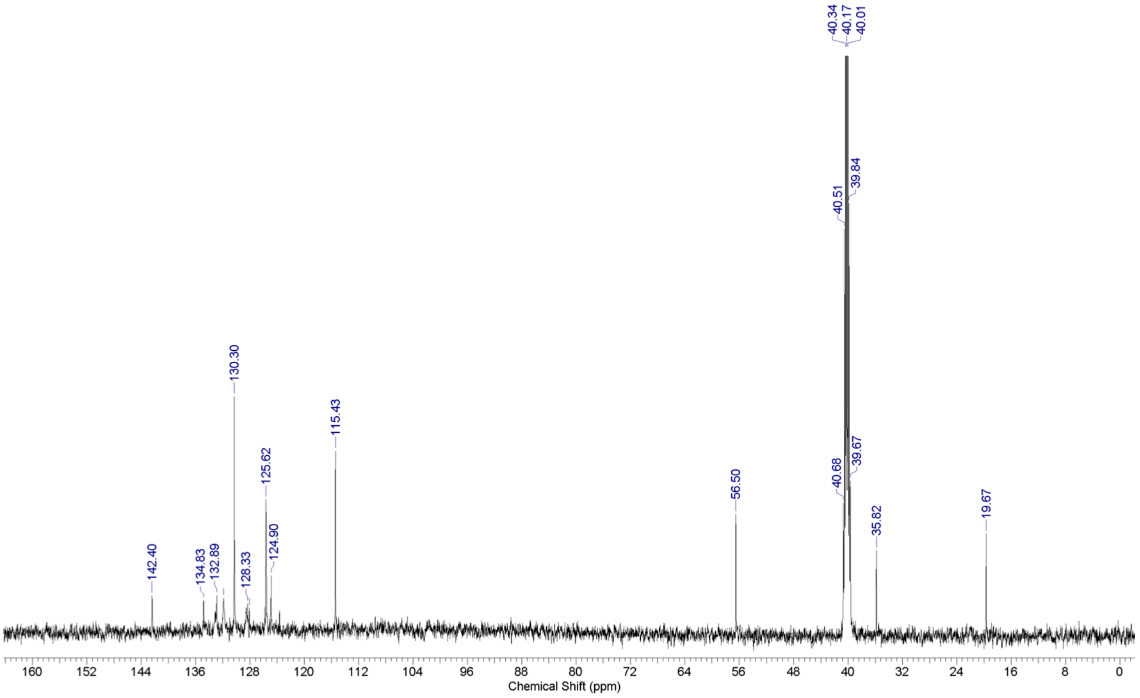

Figure S12. ^{13}C-NMR of compd **35** (125 MHz, DMSO-*d*$_6$).

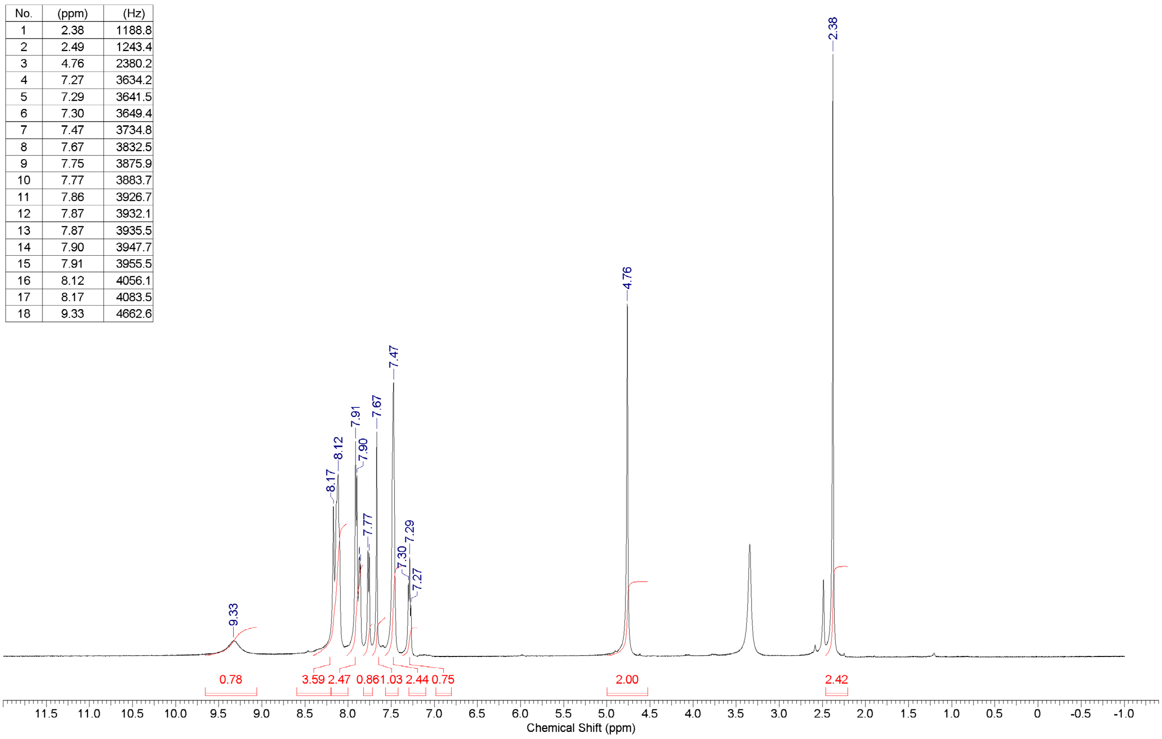

No.	(ppm)	(Hz)
1	2.38	1188.8
2	2.49	1243.4
3	4.76	2380.2
4	7.27	3634.2
5	7.29	3641.5
6	7.30	3649.4
7	7.47	3734.8
8	7.67	3832.5
9	7.75	3875.9
10	7.77	3883.7
11	7.86	3926.7
12	7.87	3932.1
13	7.87	3935.5
14	7.90	3947.7
15	7.91	3955.5
16	8.12	4056.1
17	8.17	4083.5
18	9.33	4662.6

Figure S13. ^{1}H-NMR of compd **37** (500 MHz, DMSO-*d*$_6$).

No.	(ppm)	(Hz)
1	19.57	2460.0
2	34.87	4383.3
3	39.68	4987.0
4	39.84	5007.7
5	40.01	5028.7
6	40.18	5049.8
7	40.34	5070.9
8	40.51	5092.0
9	40.68	5112.6
10	124.85	15692.1
11	125.98	15835.2
12	126.62	15914.7
13	126.95	15956.4
14	128.63	16167.6
15	128.84	16194.0
16	129.14	16231.3
17	130.00	16340.2
18	131.92	16581.3
19	132.22	16619.5
20	132.71	16680.5
21	133.52	16781.9
22	133.97	16839.0
23	137.01	17220.6

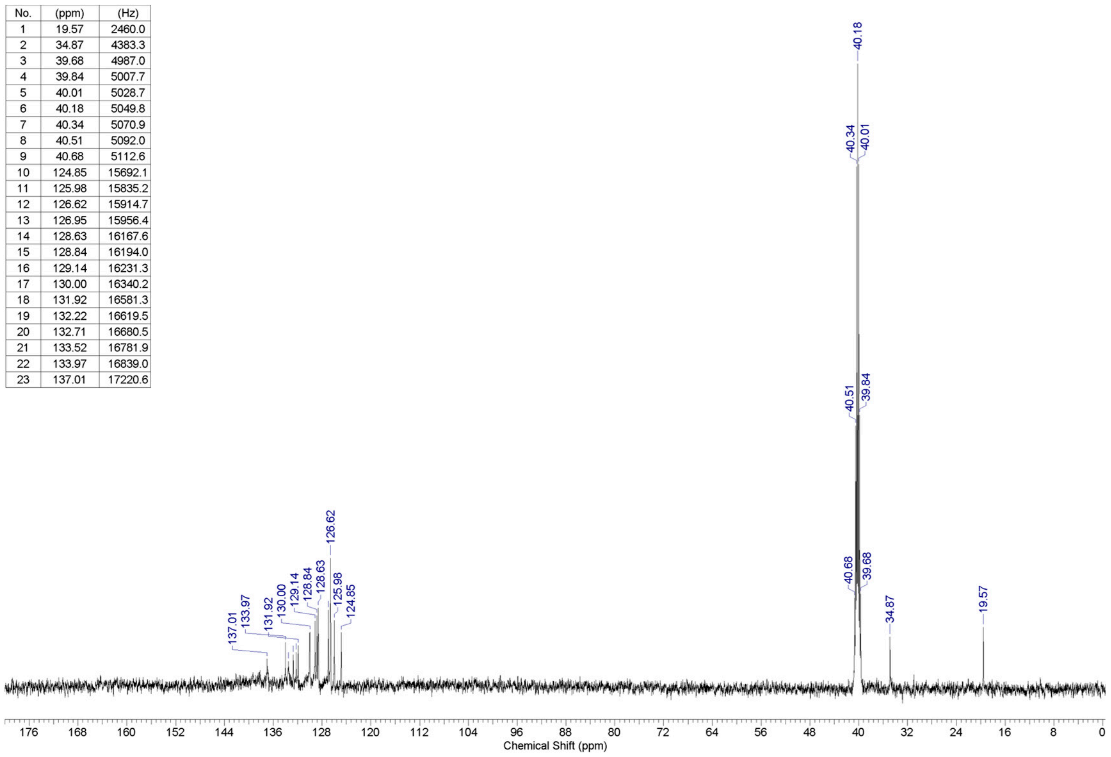

Figure S14. ^{13}C-NMR of compd **37** (125 MHz, DMSO-d_6).

No.	(ppm)	(Hz)
1	2.42	1211.2
2	3.88	1938.3
3	4.73	2361.7
4	7.05	3525.8
5	7.07	3534.6
6	7.28	3637.6
7	7.29	3644.5
8	7.31	3652.8
9	7.46	3729.4
10	7.47	3735.3
11	7.49	3742.1
12	7.50	3748.5
13	7.51	3755.3
14	7.60	3799.7
15	7.77	3884.2
16	7.79	3892.5
17	7.88	3937.9
18	7.89	3945.8
19	7.93	3963.3
20	7.94	3970.7
21	8.08	4040.0
22	8.10	4047.8
23	8.13	4065.4
24	9.00	4498.5
25	14.39	7191.6

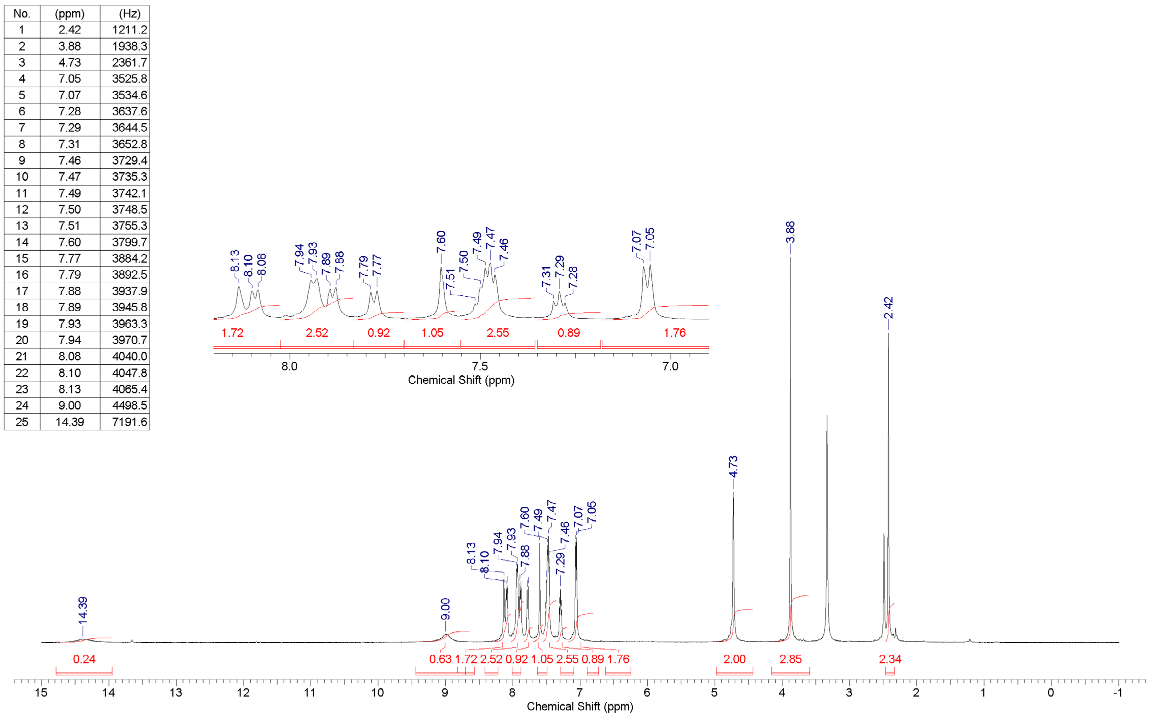

Figure S15. ^{1}H-NMR of compd **38** (500 MHz, DMSO-d_6).

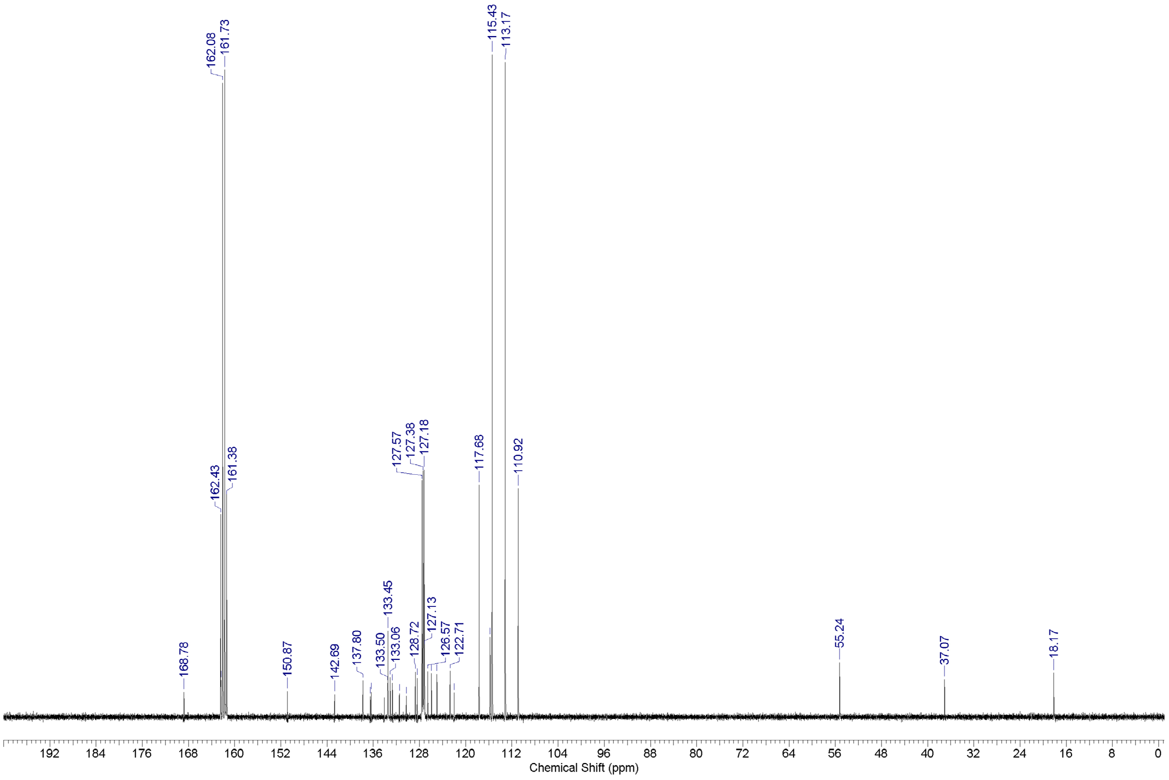

Figure S16. ^{13}C-NMR of compd **38** (125 MHz, TFA).

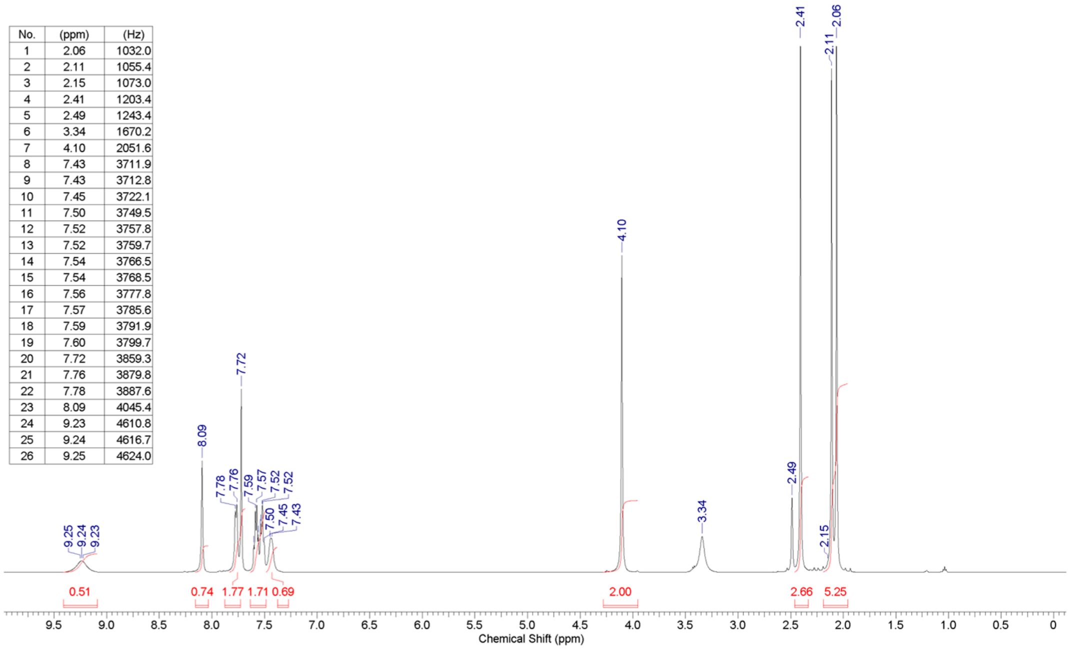

No.	(ppm)	(Hz)
1	2.06	1032.0
2	2.11	1055.4
3	2.15	1073.0
4	2.41	1203.4
5	2.49	1243.4
6	3.34	1670.2
7	4.10	2051.6
8	7.43	3711.9
9	7.43	3712.8
10	7.45	3722.1
11	7.50	3749.5
12	7.52	3757.8
13	7.52	3759.7
14	7.54	3766.5
15	7.54	3768.5
16	7.56	3777.8
17	7.57	3785.6
18	7.59	3791.9
19	7.60	3799.7
20	7.72	3859.3
21	7.76	3879.8
22	7.78	3887.6
23	8.09	4045.4
24	9.23	4610.8
25	9.24	4616.7
26	9.25	4624.0

Figure S17. ^{1}H-NMR of compd **39** (500 MHz, DMSO-d_6).

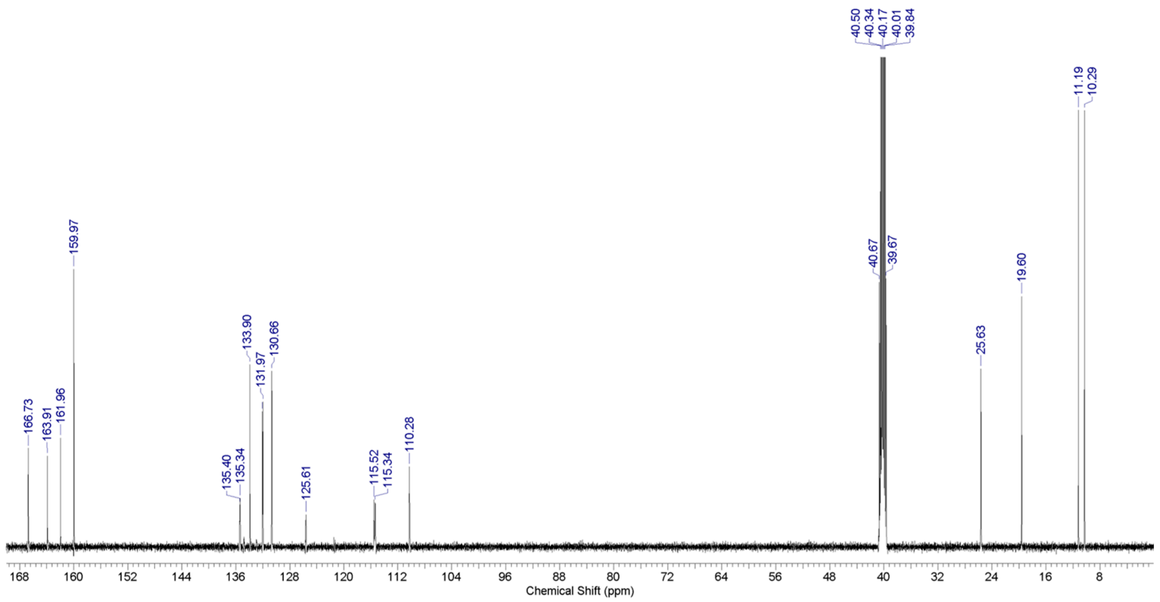

Figure S18. ^{13}C-NMR of compd **39** (125 MHz, DMSO-d_6).

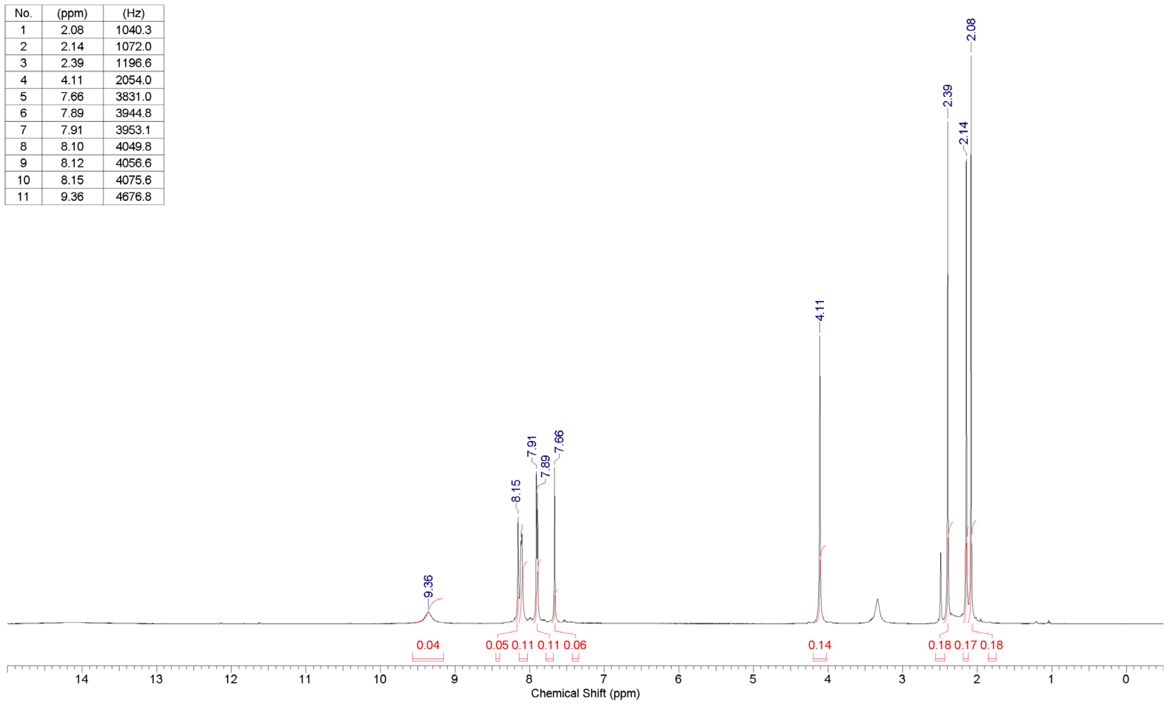

No.	(ppm)	(Hz)
1	2.08	1040.3
2	2.14	1072.0
3	2.39	1196.6
4	4.11	2054.0
5	7.66	3831.0
6	7.89	3944.8
7	7.91	3953.1
8	8.10	4049.8
9	8.12	4056.6
10	8.15	4075.6
11	9.36	4676.8

Figure S19. ^{1}H-NMR of compd **40** (500 MHz, DMSO-d_6).

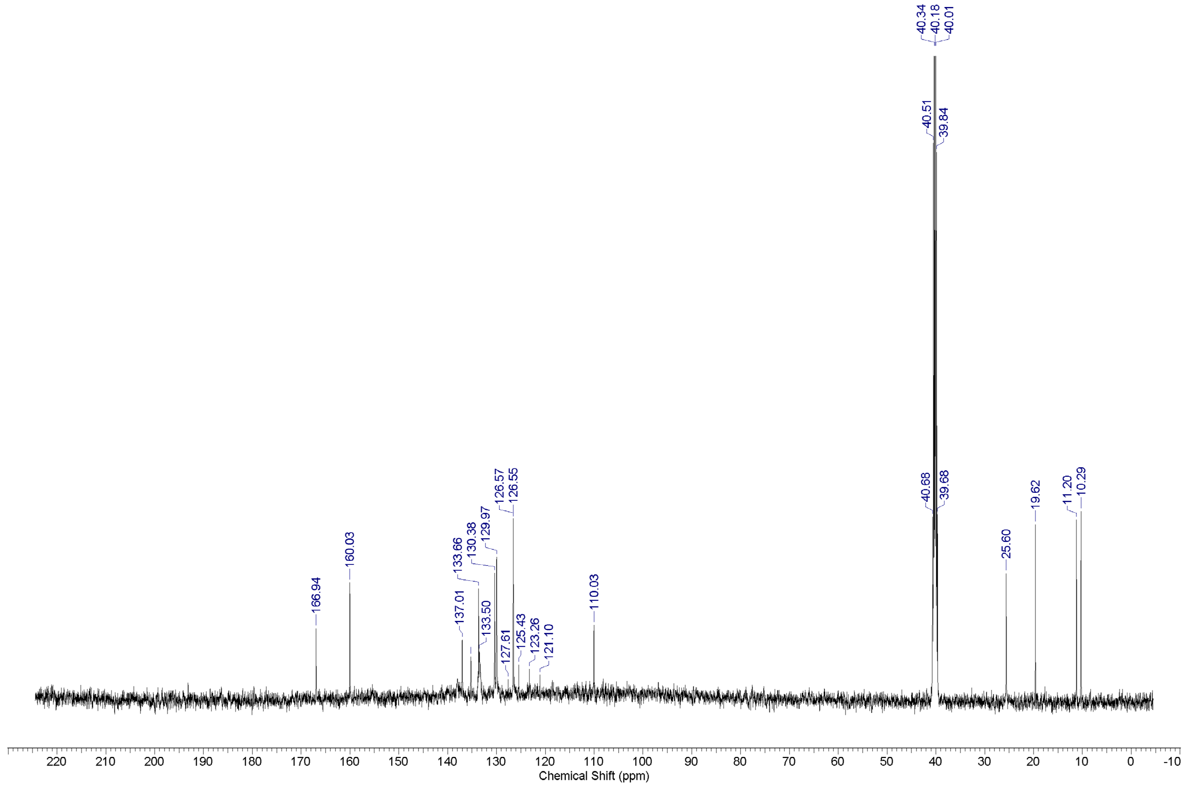

Figure S20. ^{13}C-NMR of compd **40** (125 MHz, DMSO-*d*$_6$).

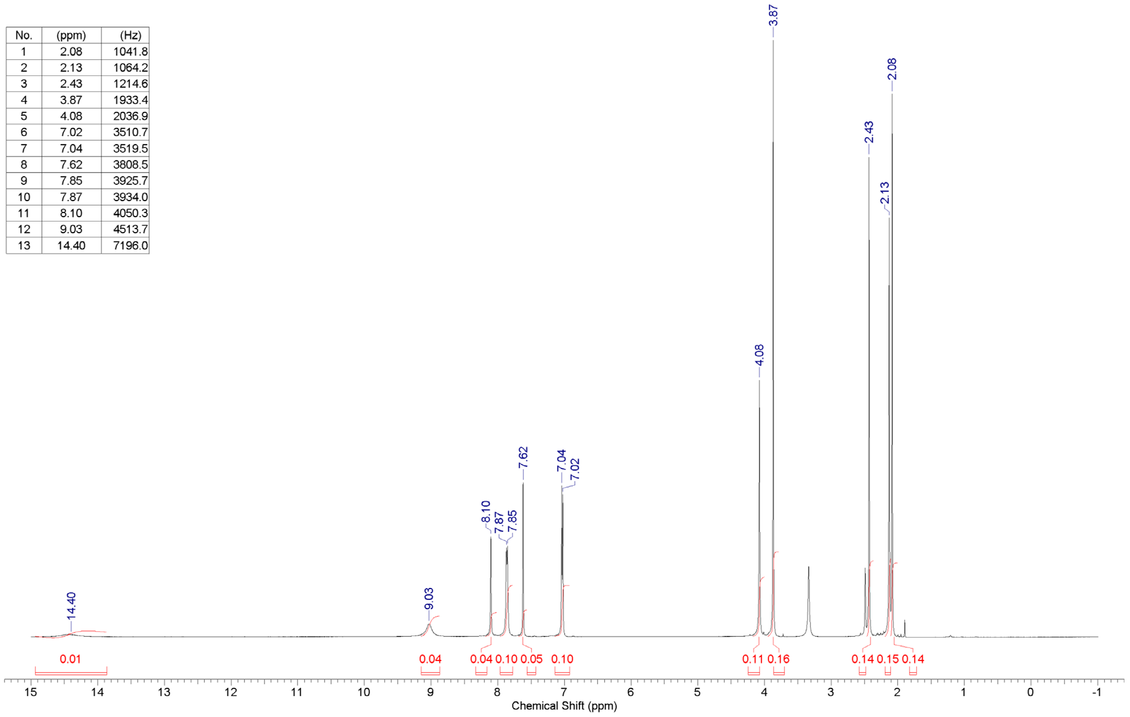

No.	(ppm)	(Hz)
1	2.08	1041.8
2	2.13	1064.2
3	2.43	1214.6
4	3.87	1933.4
5	4.08	2036.9
6	7.02	3510.7
7	7.04	3519.5
8	7.62	3808.5
9	7.85	3925.7
10	7.87	3934.0
11	8.10	4050.3
12	9.03	4513.7
13	14.40	7196.0

Figure S21. ^{1}H-NMR of compd **41** (500 MHz, DMSO-*d*$_6$).

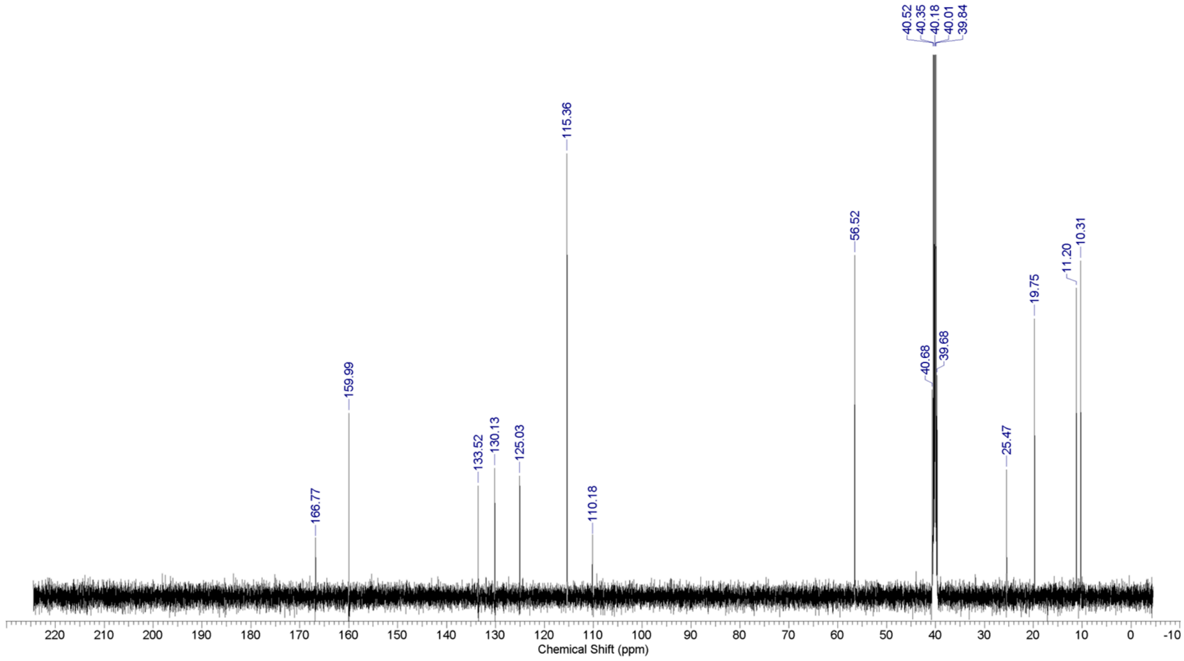

Figure S22. ^{13}C-NMR of compd **41** (125 MHz, DMSO-*d*$_6$).

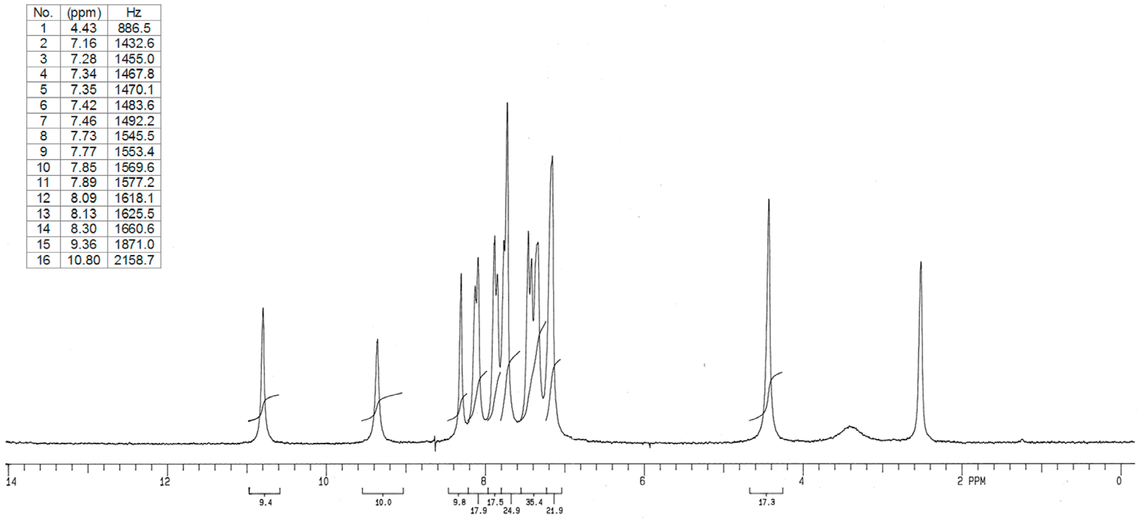

No.	(ppm)	Hz
1	4.43	886.5
2	7.16	1432.6
3	7.28	1455.0
4	7.34	1467.8
5	7.35	1470.1
6	7.42	1483.6
7	7.46	1492.2
8	7.73	1545.5
9	7.77	1553.4
10	7.85	1569.6
11	7.89	1577.2
12	8.09	1618.1
13	8.13	1625.5
14	8.30	1660.6
15	9.36	1871.0
16	10.80	2158.7

Figure S23. ^{1}H-NMR of compd **46** (200 MHz, DMSO-*d*$_6$).

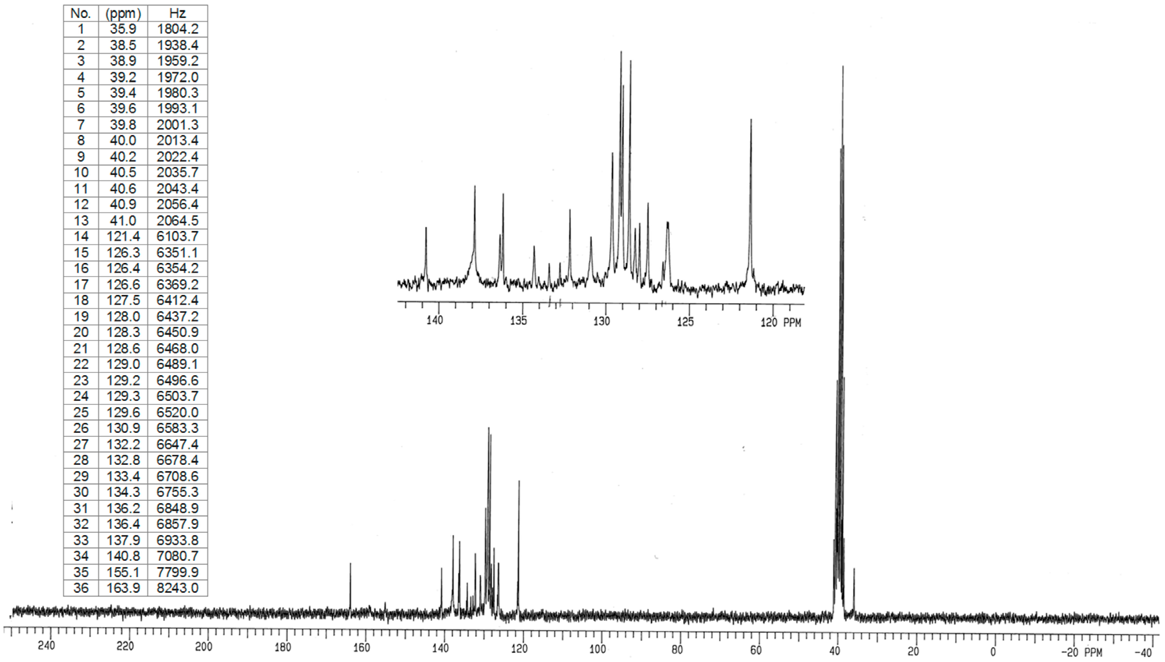

No.	(ppm)	Hz
1	35.9	1804.2
2	38.5	1938.4
3	38.9	1959.2
4	39.2	1972.0
5	39.4	1980.3
6	39.6	1993.1
7	39.8	2001.3
8	40.0	2013.4
9	40.2	2022.4
10	40.5	2035.7
11	40.6	2043.4
12	40.9	2056.4
13	41.0	2064.5
14	121.4	6103.7
15	126.3	6351.1
16	126.4	6354.2
17	126.6	6369.2
18	127.5	6412.4
19	128.0	6437.2
20	128.3	6450.9
21	128.6	6468.0
22	129.0	6489.1
23	129.2	6496.6
24	129.3	6503.7
25	129.6	6520.0
26	130.9	6583.3
27	132.2	6647.4
28	132.8	6678.4
29	133.4	6708.6
30	134.3	6755.3
31	136.2	6848.9
32	136.4	6857.9
33	137.9	6933.8
34	140.8	7080.7
35	155.1	7799.9
36	163.9	8243.0

Figure S24. ^{13}C-NMR of compd **46** (50 MHz, DMSO-d_6).

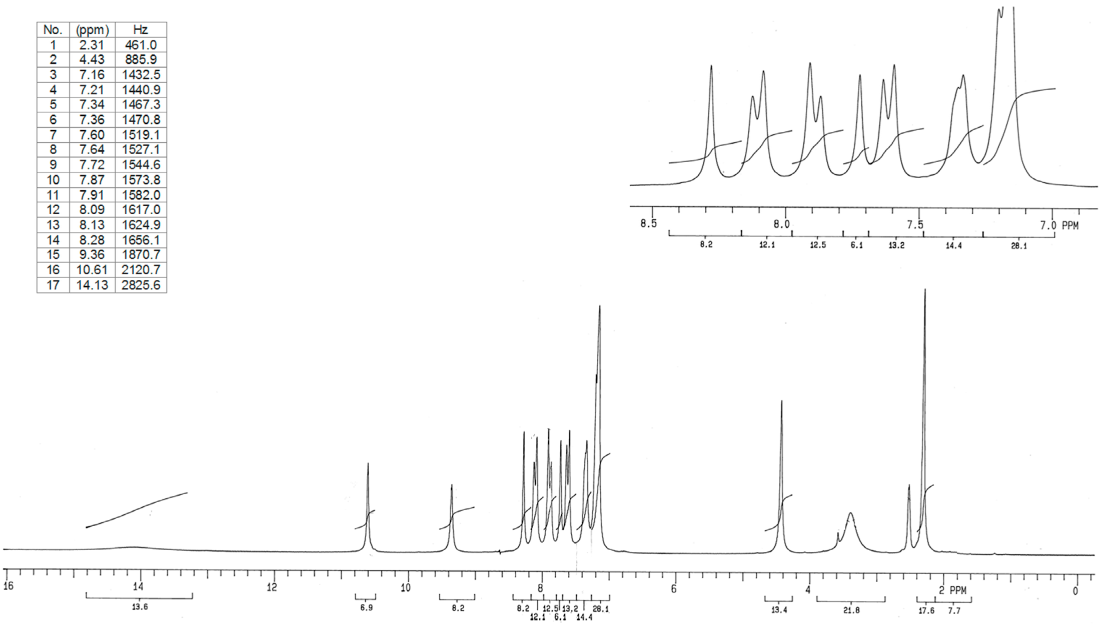

No.	(ppm)	Hz
1	2.31	461.0
2	4.43	885.9
3	7.16	1432.5
4	7.21	1440.9
5	7.34	1467.3
6	7.36	1470.8
7	7.60	1519.1
8	7.64	1527.1
9	7.72	1544.6
10	7.87	1573.8
11	7.91	1582.0
12	8.09	1617.0
13	8.13	1624.9
14	8.28	1656.1
15	9.36	1870.7
16	10.61	2120.7
17	14.13	2825.6

Figure S25. ^{1}H-NMR of compd **52** (200 MHz, DMSO-d_6).

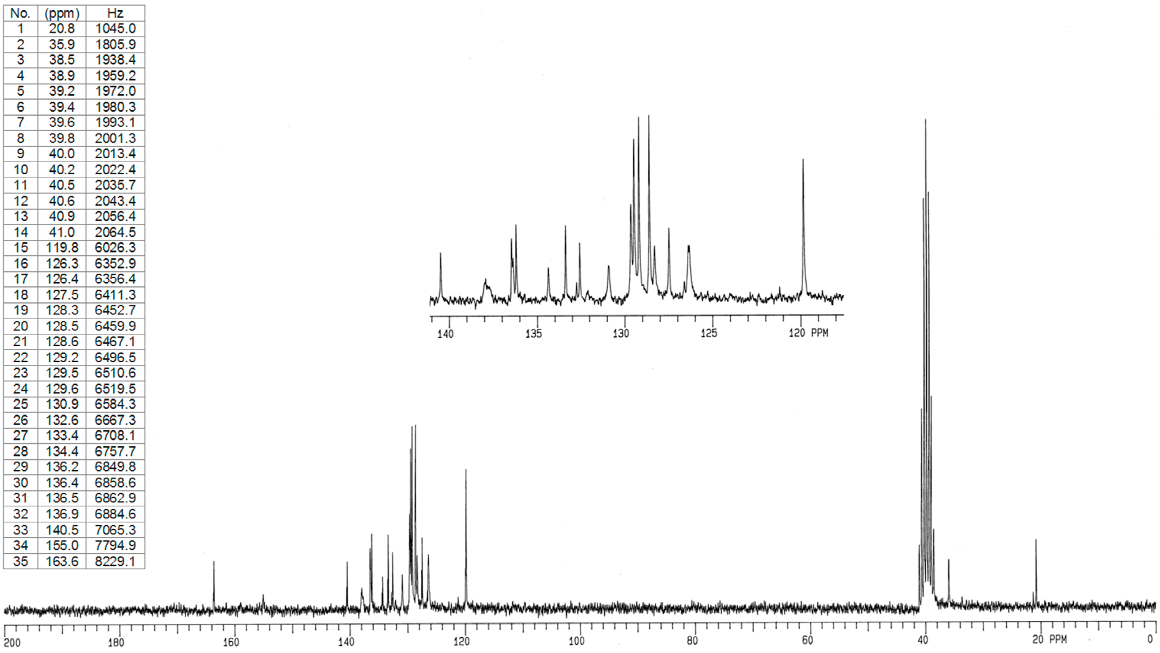

No.	(ppm)	Hz
1	20.8	1045.0
2	35.9	1805.9
3	38.5	1938.4
4	38.9	1959.2
5	39.2	1972.0
6	39.4	1980.3
7	39.6	1993.1
8	39.8	2001.3
9	40.0	2013.4
10	40.2	2022.4
11	40.5	2035.7
12	40.6	2043.4
13	40.9	2056.4
14	41.0	2064.5
15	119.8	6026.3
16	126.3	6352.9
17	126.4	6356.4
18	127.5	6411.3
19	128.3	6452.7
20	128.5	6459.9
21	128.6	6467.1
22	129.2	6496.5
23	129.5	6510.6
24	129.6	6519.5
25	130.9	6584.3
26	132.6	6667.3
27	133.4	6708.1
28	134.4	6757.7
29	136.2	6849.8
30	136.4	6858.6
31	136.5	6862.9
32	136.9	6884.6
33	140.5	7065.3
34	155.0	7794.9
35	163.6	8229.1

Figure S26. ^{13}C-NMR of compd **52** (50 MHz, DMSO-*d*$_6$).

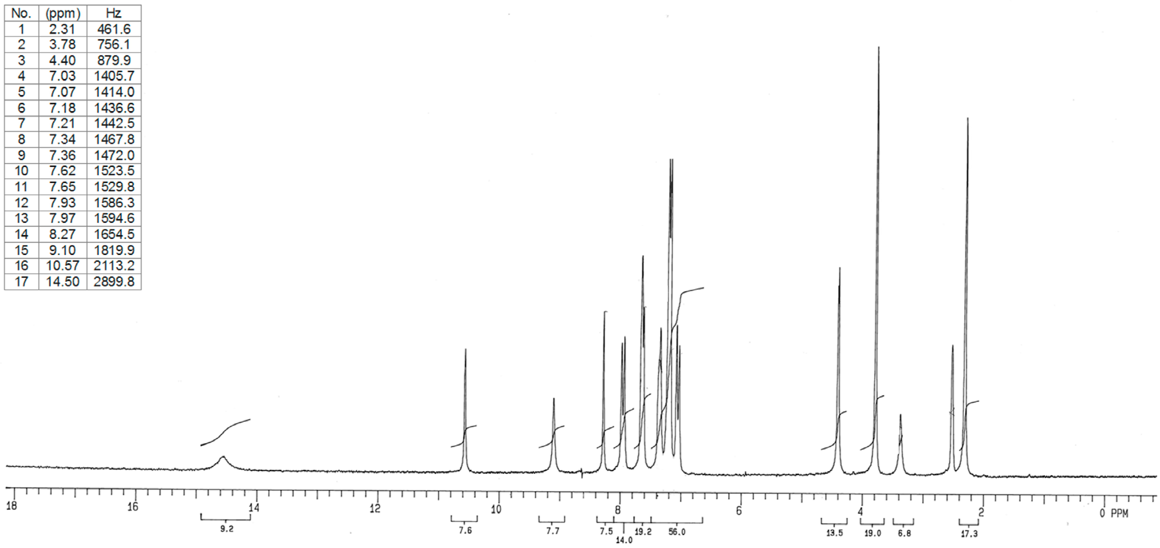

No.	(ppm)	Hz
1	2.31	461.6
2	3.78	756.1
3	4.40	879.9
4	7.03	1405.7
5	7.07	1414.0
6	7.18	1436.6
7	7.21	1442.5
8	7.34	1467.8
9	7.36	1472.0
10	7.62	1523.5
11	7.65	1529.8
12	7.93	1586.3
13	7.97	1594.6
14	8.27	1654.5
15	9.10	1819.9
16	10.57	2113.2
17	14.50	2899.8

Figure S27. ^{1}H-NMR of compd **53** (200 MHz, DMSO-*d*$_6$).

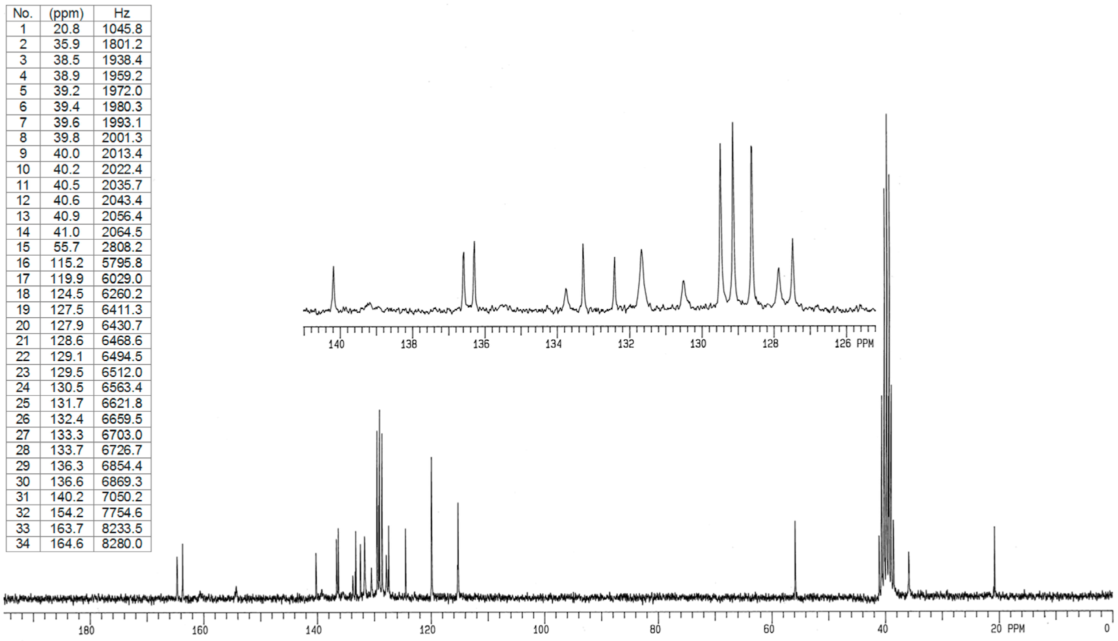

No.	(ppm)	Hz
1	20.8	1045.8
2	35.9	1801.2
3	38.5	1938.4
4	38.9	1959.2
5	39.2	1972.0
6	39.4	1980.3
7	39.6	1993.1
8	39.8	2001.3
9	40.0	2013.4
10	40.2	2022.4
11	40.5	2035.7
12	40.6	2043.4
13	40.9	2056.4
14	41.0	2064.5
15	55.7	2808.2
16	115.2	5795.8
17	119.9	6029.0
18	124.5	6260.2
19	127.5	6411.3
20	127.9	6430.7
21	128.6	6468.6
22	129.1	6494.5
23	129.5	6512.0
24	130.5	6563.4
25	131.7	6621.8
26	132.4	6659.5
27	133.3	6703.0
28	133.7	6726.7
29	136.3	6854.4
30	136.6	6869.3
31	140.2	7050.2
32	154.2	7754.6
33	163.7	8233.5
34	164.6	8280.0

Figure S28. ^{13}C-NMR of compd **53** (50 MHz, DMSO-d_6).

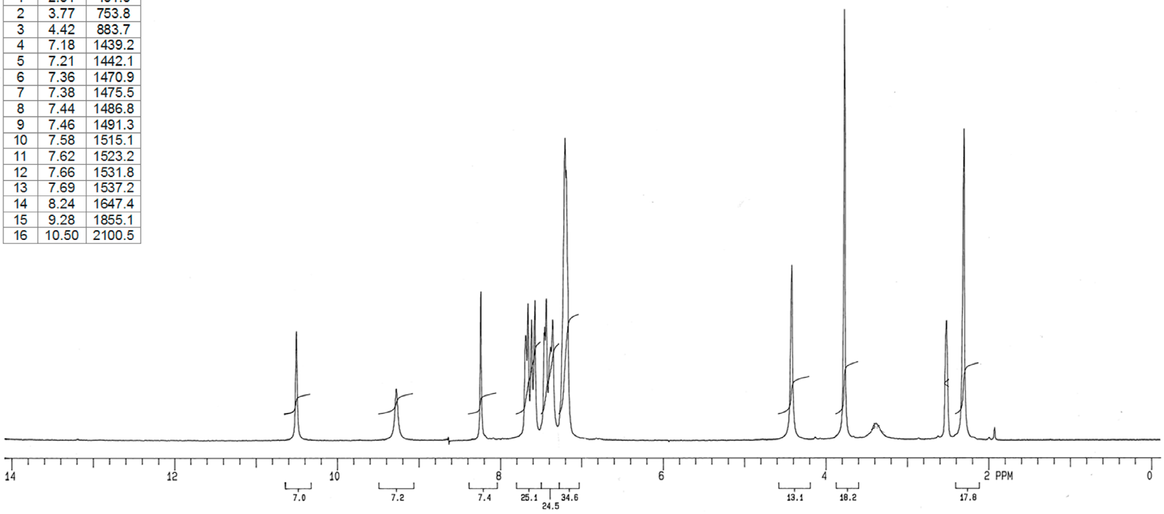

No.	(ppm)	Hz
1	2.31	461.6
2	3.77	753.8
3	4.42	883.7
4	7.18	1439.2
5	7.21	1442.1
6	7.36	1470.9
7	7.38	1475.5
8	7.44	1486.8
9	7.46	1491.3
10	7.58	1515.1
11	7.62	1523.2
12	7.66	1531.8
13	7.69	1537.2
14	8.24	1647.4
15	9.28	1855.1
16	10.50	2100.5

Figure S29. ^{1}H-NMR of compd **54** (200 MHz, DMSO-d_6).

No.	(ppm)	Hz
1	20.8	1046.7
2	35.9	1804.4
3	38.5	1938.4
4	38.9	1959.2
5	39.2	1972.0
6	39.4	1980.3
7	39.6	1993.1
8	39.8	2001.3
9	40.0	2013.4
10	40.2	2022.4
11	40.5	2035.7
12	40.6	2043.4
13	40.9	2056.4
14	41.0	2064.5
15	55.7	2799.1
16	114.2	5741.6
17	119.9	6029.7
18	120.1	6038.1
19	121.3	6101.7
20	127.5	6413.3
21	127.9	6465.1
22	128.6	6469.7
23	129.2	6496.8
24	129.4	6509.2
25	130.5	6561.4
26	130.9	6583.6
27	132.5	6663.5
28	133.2	6699.5
29	133.7	6721.6
30	136.2	6849.9
31	136.5	6867.1
32	140.3	7055.5
33	154.2	7754.7
34	159.9	8044.6
35	163.5	8221.2

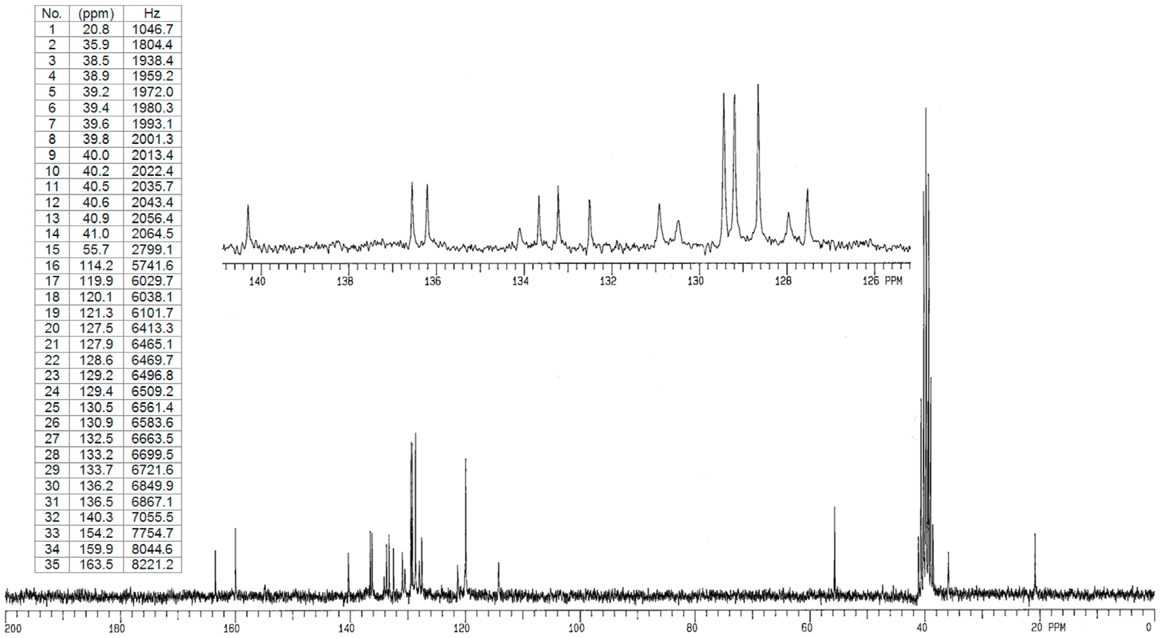

Figure S30. ^{13}C-NMR of compd **54** (50 MHz, DMSO-*d*$_6$).

No.	(ppm)	Hz
1	2.29	458.8
2	3.71	741.0
3	3.80	758.9
4	4.43	886.1
5	7.14	1426.8
6	7.18	1434.9
7	7.23	1446.7
8	7.26	1452.7
9	7.40	1480.5
10	7.47	1493.3
11	7.52	1502.8
12	7.56	1510.7
13	7.63	1525.3
14	8.20	1640.6
15	9.32	1864.0
16	10.50	2090.0
17	14.20	2839.7

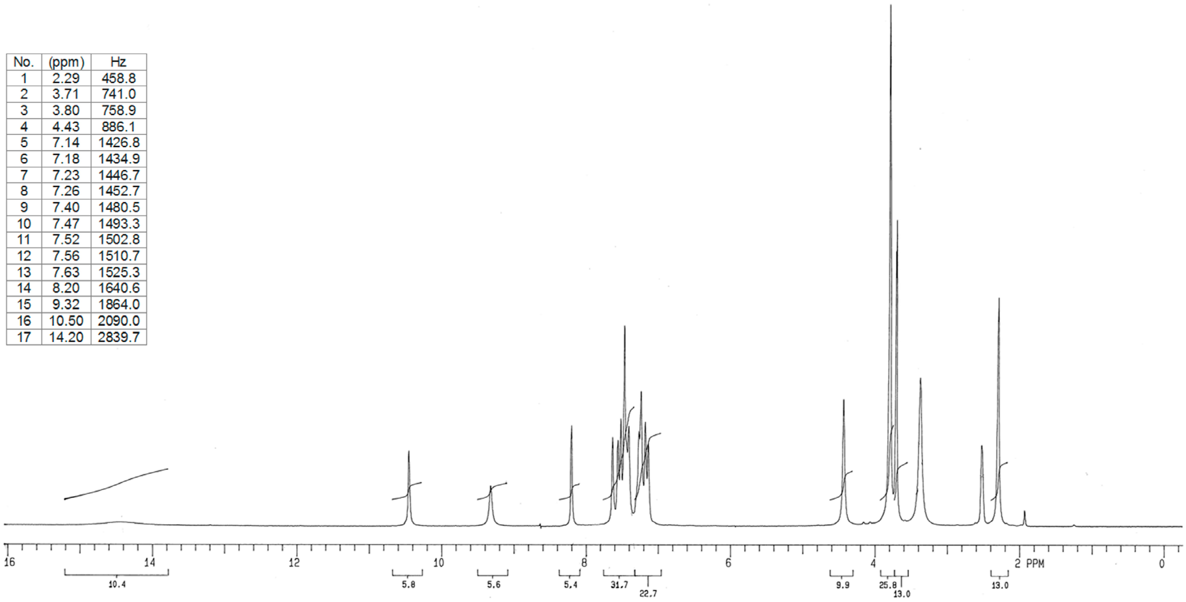

Figure S31. ^{1}H-NMR of compd **56** (200 MHz, DMSO-*d*$_6$).

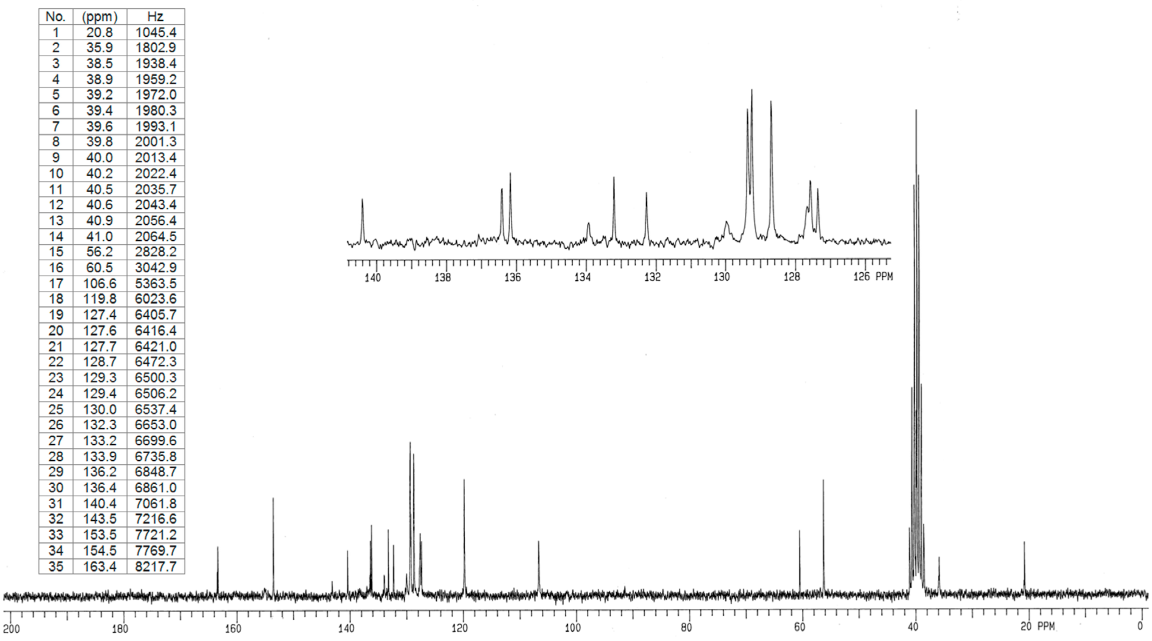

No.	(ppm)	Hz
1	20.8	1045.4
2	35.9	1802.9
3	38.5	1938.4
4	38.9	1959.2
5	39.2	1972.0
6	39.4	1980.3
7	39.6	1993.1
8	39.8	2001.3
9	40.0	2013.4
10	40.2	2022.4
11	40.5	2035.7
12	40.6	2043.4
13	40.9	2056.4
14	41.0	2064.5
15	56.2	2828.2
16	60.5	3042.9
17	106.6	5363.5
18	119.8	6023.6
19	127.4	6405.7
20	127.6	6416.4
21	127.7	6421.0
22	128.7	6472.3
23	129.3	6500.3
24	129.4	6506.2
25	130.0	6537.4
26	132.3	6653.0
27	133.2	6699.6
28	133.9	6735.8
29	136.2	6848.7
30	136.4	6861.0
31	140.4	7061.8
32	143.5	7216.6
33	153.5	7721.2
34	154.5	7769.7
35	163.4	8217.7

Figure S32. ^{13}C-NMR of compd **56** (50 MHz, DMSO-*d*$_6$).

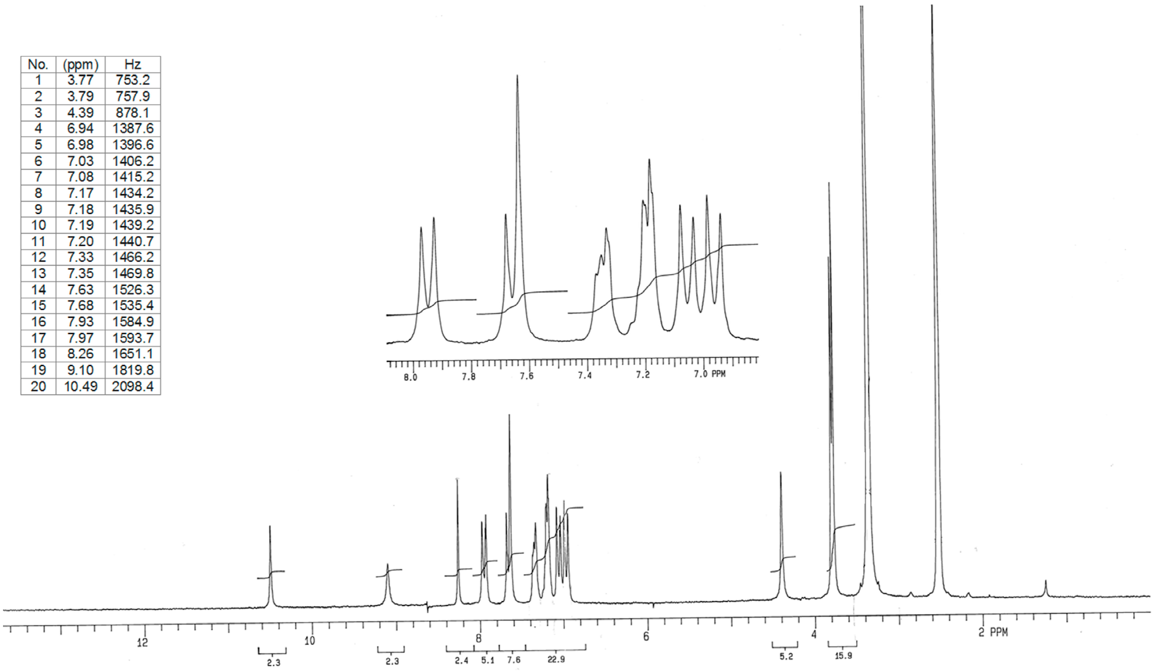

No.	(ppm)	Hz
1	3.77	753.2
2	3.79	757.9
3	4.39	878.1
4	6.94	1387.6
5	6.98	1396.6
6	7.03	1406.2
7	7.08	1415.2
8	7.17	1434.2
9	7.18	1435.9
10	7.19	1439.2
11	7.20	1440.7
12	7.33	1466.2
13	7.35	1469.8
14	7.63	1526.3
15	7.68	1535.4
16	7.93	1584.9
17	7.97	1593.7
18	8.26	1651.1
19	9.10	1819.8
20	10.49	2098.4

Figure S33. ^{1}H-NMR of compd **57** (200 MHz, DMSO-*d*$_6$).

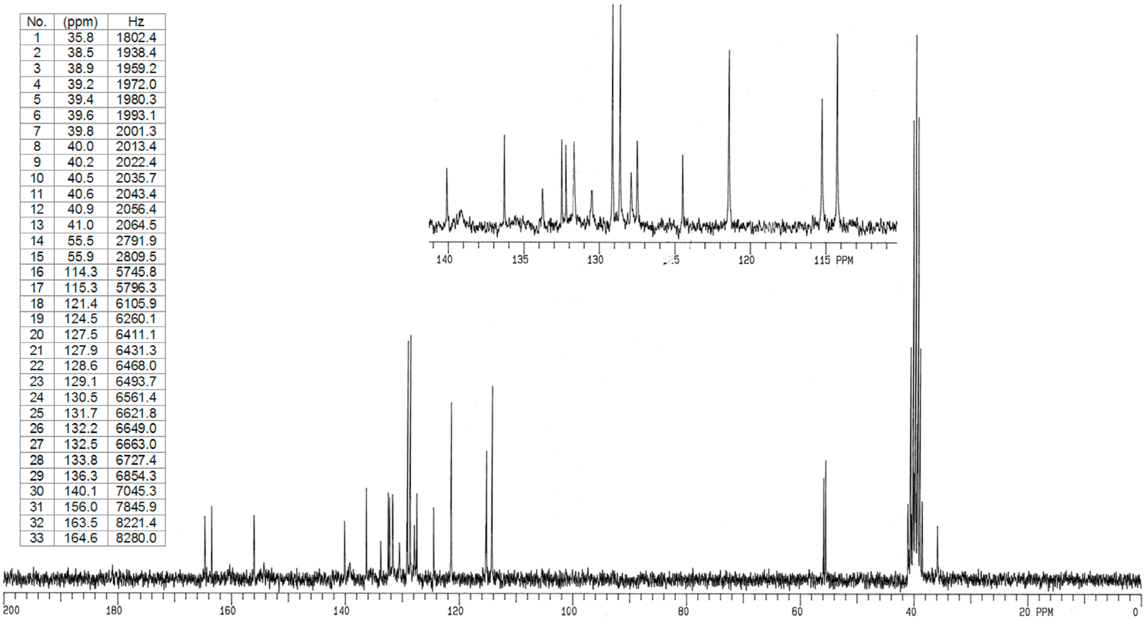

No.	(ppm)	Hz
1	35.8	1802.4
2	38.5	1938.4
3	38.9	1959.2
4	39.2	1972.0
5	39.4	1980.3
6	39.6	1993.1
7	39.8	2001.3
8	40.0	2013.4
9	40.2	2022.4
10	40.5	2035.7
11	40.6	2043.4
12	40.9	2056.4
13	41.0	2064.5
14	55.5	2791.9
15	55.9	2809.5
16	114.3	5745.8
17	115.3	5796.3
18	121.4	6105.9
19	124.5	6260.1
20	127.5	6411.1
21	127.9	6431.3
22	128.6	6468.0
23	129.1	6493.7
24	130.5	6561.4
25	131.7	6621.8
26	132.2	6649.0
27	132.5	6663.0
28	133.8	6727.4
29	136.3	6854.3
30	140.1	7045.3
31	156.0	7845.9
32	163.5	8221.4
33	164.6	8280.0

Figure S34. ^{13}C-NMR of compd **57** (50 MHz, DMSO-d_6).

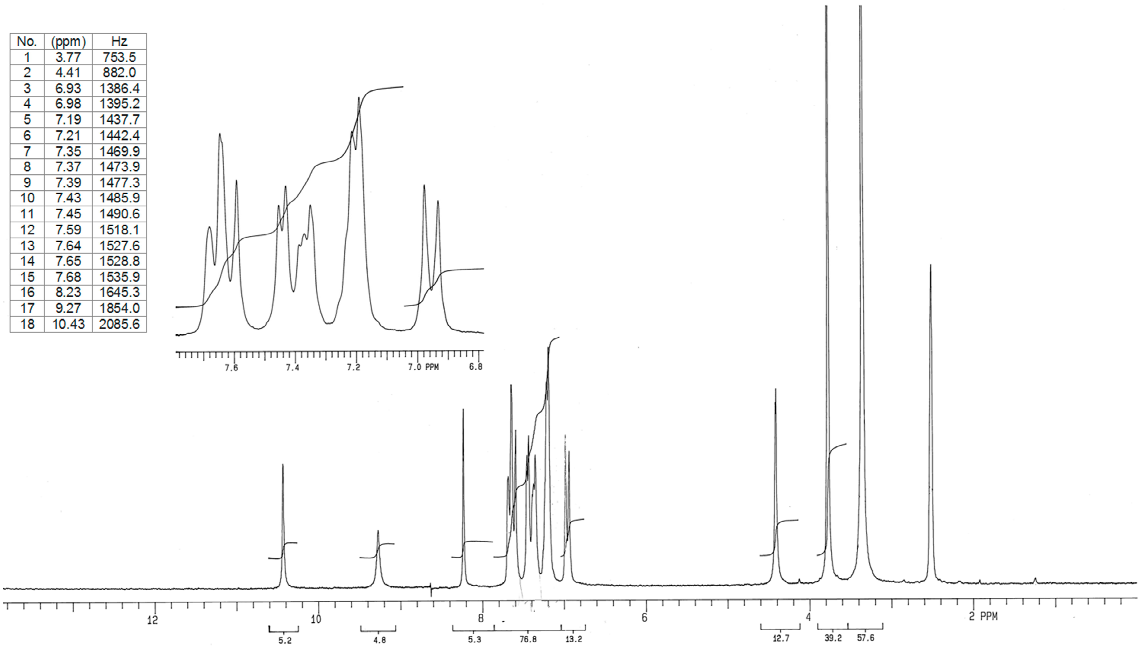

No.	(ppm)	Hz
1	3.77	753.5
2	4.41	882.0
3	6.93	1386.4
4	6.98	1395.2
5	7.19	1437.7
6	7.21	1442.4
7	7.35	1469.9
8	7.37	1473.9
9	7.39	1477.3
10	7.43	1485.9
11	7.45	1490.6
12	7.59	1518.1
13	7.64	1527.6
14	7.65	1528.8
15	7.68	1535.9
16	8.23	1645.3
17	9.27	1854.0
18	10.43	2085.6

Figure S35. ^{1}H-NMR of compd **58** (200 MHz, DMSO-d_6).

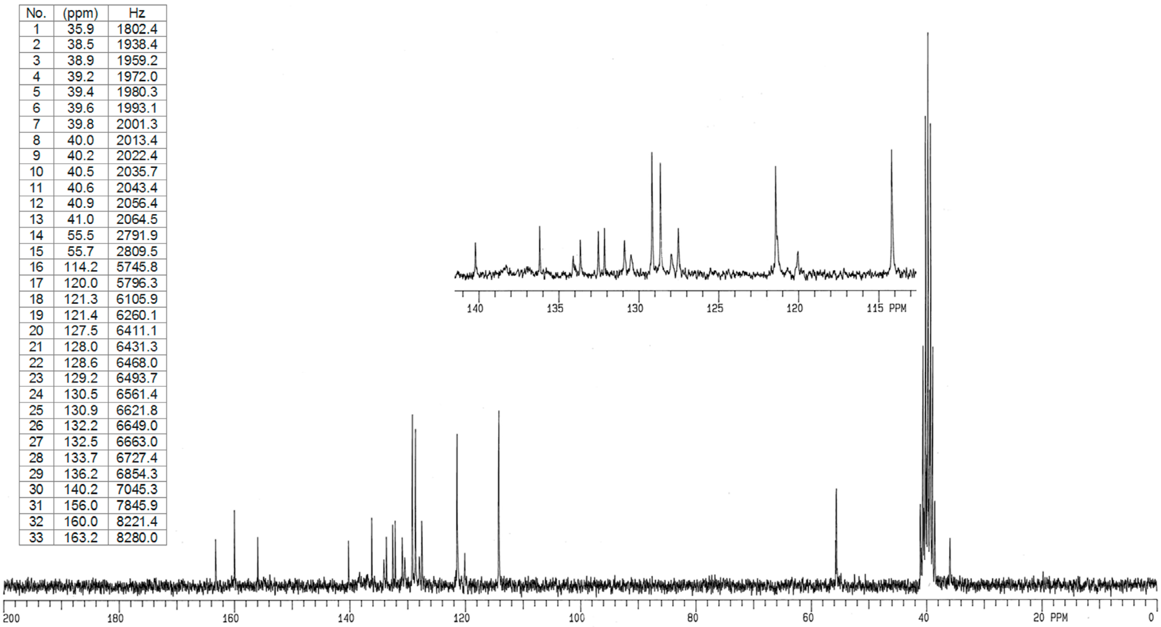

No.	(ppm)	Hz
1	35.9	1802.4
2	38.5	1938.4
3	38.9	1959.2
4	39.2	1972.0
5	39.4	1980.3
6	39.6	1993.1
7	39.8	2001.3
8	40.0	2013.4
9	40.2	2022.4
10	40.5	2035.7
11	40.6	2043.4
12	40.9	2056.4
13	41.0	2064.5
14	55.5	2791.9
15	55.7	2809.5
16	114.2	5745.8
17	120.0	5796.3
18	121.3	6105.9
19	121.4	6260.1
20	127.5	6411.1
21	128.0	6431.3
22	128.6	6468.0
23	129.2	6493.7
24	130.5	6561.4
25	130.9	6621.8
26	132.2	6649.0
27	132.5	6663.0
28	133.7	6727.4
29	136.2	6854.3
30	140.2	7045.3
31	156.0	7845.9
32	160.0	8221.4
33	163.2	8280.0

Figure S36. ^{13}C-NMR of compd **58** (50 MHz, DMSO-d_6).

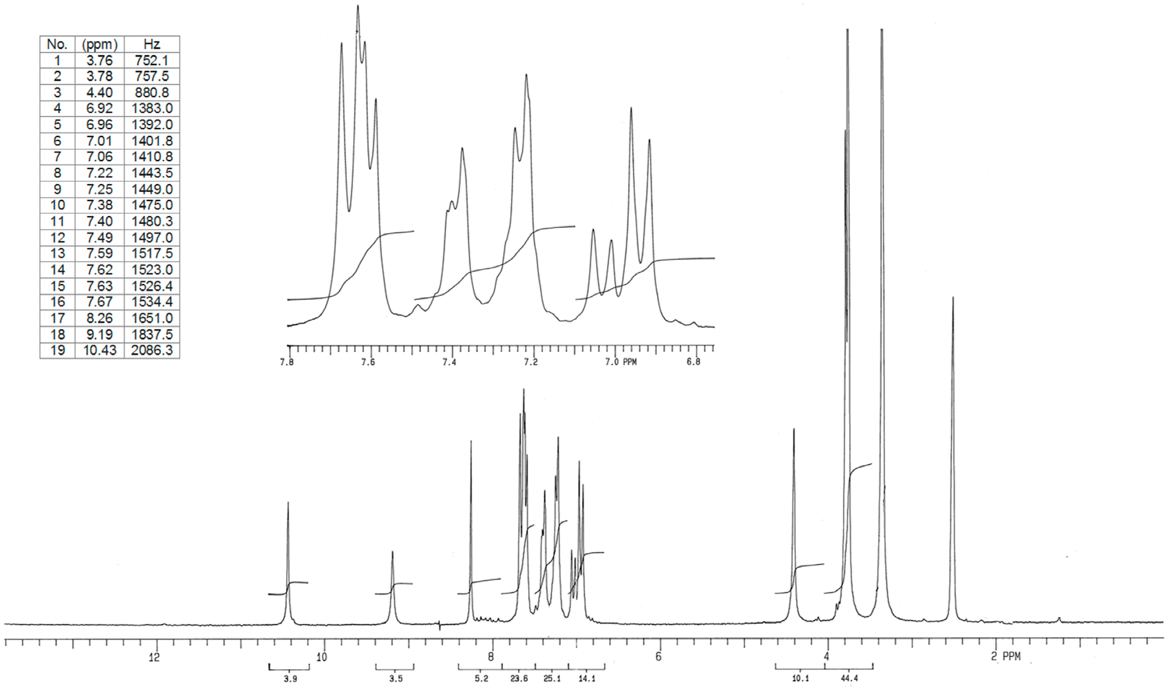

No.	(ppm)	Hz
1	3.76	752.1
2	3.78	757.5
3	4.40	880.8
4	6.92	1383.0
5	6.96	1392.0
6	7.01	1401.8
7	7.06	1410.8
8	7.22	1443.5
9	7.25	1449.0
10	7.38	1475.0
11	7.40	1480.3
12	7.49	1497.0
13	7.59	1517.5
14	7.62	1523.0
15	7.63	1526.4
16	7.67	1534.4
17	8.26	1651.0
18	9.19	1837.5
19	10.43	2086.3

Figure S37. ^{1}H-NMR of compd **59** (200 MHz, DMSO-d_6).

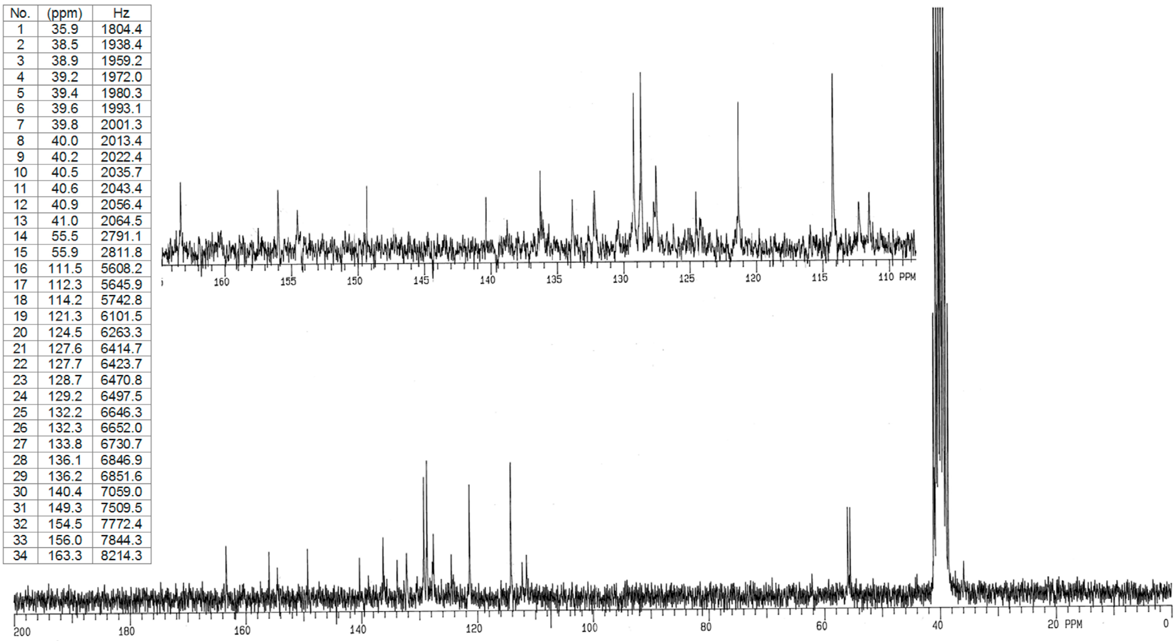

No.	(ppm)	Hz
1	35.9	1804.4
2	38.5	1938.4
3	38.9	1959.2
4	39.2	1972.0
5	39.4	1980.3
6	39.6	1993.1
7	39.8	2001.3
8	40.0	2013.4
9	40.2	2022.4
10	40.5	2035.7
11	40.6	2043.4
12	40.9	2056.4
13	41.0	2064.5
14	55.5	2791.1
15	55.9	2811.8
16	111.5	5608.2
17	112.3	5645.9
18	114.2	5742.8
19	121.3	6101.5
20	124.5	6263.3
21	127.6	6414.7
22	127.7	6423.7
23	128.7	6470.8
24	129.2	6497.5
25	132.2	6646.3
26	132.3	6652.0
27	133.8	6730.7
28	136.1	6846.9
29	136.2	6851.6
30	140.4	7059.0
31	149.3	7509.5
32	154.5	7772.4
33	156.0	7844.3
34	163.3	8214.3

Figure S38. ^{13}C-NMR of compd **59** (50 MHz, DMSO-d_6).
